# Galanin impairs tumor immunity in glioblastoma by promoting infiltration and ferroptosis resistance of myeloid-derived suppressor cells

**DOI:** 10.1038/s43018-026-01221-3

**Published:** 2026-08-25

**Authors:** Lizhi Pang, Yang Liu, Fei Zhou, Songlin Guo, Fatima Khan, Heba Ali, Hardik Shah, Sara Huber, Barbara Kofler, Craig M. Horbinski, Peiwen Chen

**Affiliations:** 1 Department of Cancer Sciences, Cleveland Clinic, Cleveland, OH USA; 2 Department of Neurological Surgery, Feinberg School of Medicine, Northwestern University, Chicago, IL USA; 3 Metabolomics Platform, Comprehensive Cancer Center, The University of Chicago, Chicago, IL USA; 4 Research Program for Receptor Biochemistry and Tumor Metabolism, Department of Pediatrics, University Hospital of the Paracelsus Medical University, Salzburg, Austria; 5 Cleveland Clinic Lerner College of Medicine of Case Western Reserve University, Cleveland, OH USA; 6 Case Comprehensive Cancer Center, Cleveland, OH USA

**Keywords:** CNS cancer, Tumour immunology

## Abstract

Glioblastoma (GBM) is a highly aggressive and lethal form of brain tumor that resists immunotherapy. Here we show that neuropeptide galanin (GAL) drives immunosuppression and immunotherapy resistance in GBM. Mechanistically, GBM cell-secreted GAL interacts with its receptor GALR3 on monocytic myeloid-derived suppressor cells (mMDSCs), triggering USP51-mediated deubiquitination of estrogen receptor-α, thereby engaging membrane-bound *O*-acyltransferase domain-containing 1 signaling. This signaling cascade, in turn, promotes the infiltration of immunosuppressive mMDSCs and confers ferroptosis resistance to them in the tumor microenvironment. Targeting mMDSCs through GALR3 inhibition and ferroptosis induction impairs tumor progression and activates antitumor immunity in GBM mouse models. Remarkably, combining this approach with anti-PD1 therapy achieves durable and complete tumor regression in approximately 60% of tumor-bearing mice. Our study elucidates the molecular mechanism underlying GBM immunosuppression and highlights a promising therapeutic strategy to enhance GBM response to immunotherapy.

## Abstract (web summary)

Chen and colleagues report that the neuropeptide galanin impairs antitumor immunity in glioblastoma by interacting with its receptor GALR3 on monocytic myeloid-derived suppressor cells, promoting their infiltration and ferroptosis resistance.

## Main

Glioblastoma (GBM) is the most common primary brain tumor in adults, with a median survival of 15–20 months^1^. GBM tumorigenesis is driven by somatic genomic alterations in key driver genes that regulate critical pathways involved in cell growth^2^. However, directly targeting these genetic alterations and their associated signaling pathways in GBM cells has not yielded notable clinical benefits^3,4^. Emerging evidence indicates that GBM cell-related signaling not only affects tumor biology but also shapes an immunosuppressive tumor microenvironment (TME), generating a tumor–TME symbiotic interaction promoting tumor progression and treatment resistance^5–8^.

Targeting the TME has revolutionized cancer treatment, particularly the development of immune checkpoint inhibitors (ICIs), including anti-PD1 (ref. ^9^). However, clinical trials using ICI therapies in GBM have shown limited antitumor efficiency^10,11^. Resistance to ICI therapy may be attributed to the presence of immunosuppressive myeloid cells, including myeloid-derived suppressor cells (MDSCs)^12^. MDSCs are a heterogeneous population of cells that include monocytic MDSCs (mMDSCs) and granulocytic MDSCs (gMDSCs)^13^. In GBM, MDSCs are recruited from the bone marrow (BM) in response to specific signals in a context-dependent manner^14,15^. Recent studies have highlighted the fundamental role of the central nervous system (CNS) in GBM progression^16^. It is generally accepted that GBM arises from a variety of CNS cells^17^, including glial precursor cells, neural stem cells (NSCs) and NSC-derived astrocytes, supporting a potential role of secreted CNS factors (for example, neuropeptides) in regulating the crosstalk between tumor cells and immune cells, such as MDSCs.

Tumor ferroptosis is a type of regulated cell death characterized by iron accumulation and elevated lipid peroxidation in response to factors in the TME^18^. Ferroptosis has been recognized as a potential therapeutic vulnerability that can be exploited to eliminate cancer cells across cancer types^19^. To evade eradication by ferroptosis, cancer cells develop several defense mechanisms, including a reduction in phospholipid peroxides mediated by glutathione peroxidase 4, the production of free radical-trapping antioxidants induced by ferroptosis suppressor protein 1 and sex-hormone-dependent lipid remodeling^18–20^. In addition to cancer cells, recent studies revealed that ferroptosis occurs in immune cells of the TME^21^. For example, infiltrating gMDSCs are highly susceptible to ferroptosis in response to stimuli from the TME of non-CNS tumors. As a result, these gMDSCs exhibit potent immunosuppressive activity, leading to T cell suppression and resistance to ICI therapy^18,19,22^. Despite these findings, the role of ferroptosis in GBM-associated MDSCs remains completely unknown.

In this study, we conducted an unbiased analysis aimed at identifying neuropeptides that may impact MDSC biology and the tumor–MDSC symbiosis. Our investigation revealed that neuropeptide galanin (GAL) is a key driver of mMDSC infiltration, ferroptosis resistance and immunosuppression in GBM. We demonstrated that GBM cell-derived GAL binds to its receptor GALR3 to promote mMDSC recruitment and ferroptosis resistance through USP51-mediated deubiquitination of estrogen receptor-α (ERα) signaling. Combining GALR3 inhibition with ferroptosis induction in GBM mouse models effectively impairs tumor progression and activates antitumor immunity. Lastly, the addition of anti-PD1 therapy to this combination leads to durable GBM remissions in mouse models.

## Results

### Tumor-cell-derived GAL triggers MDSC infiltration in GBM

**Fig. 1:**
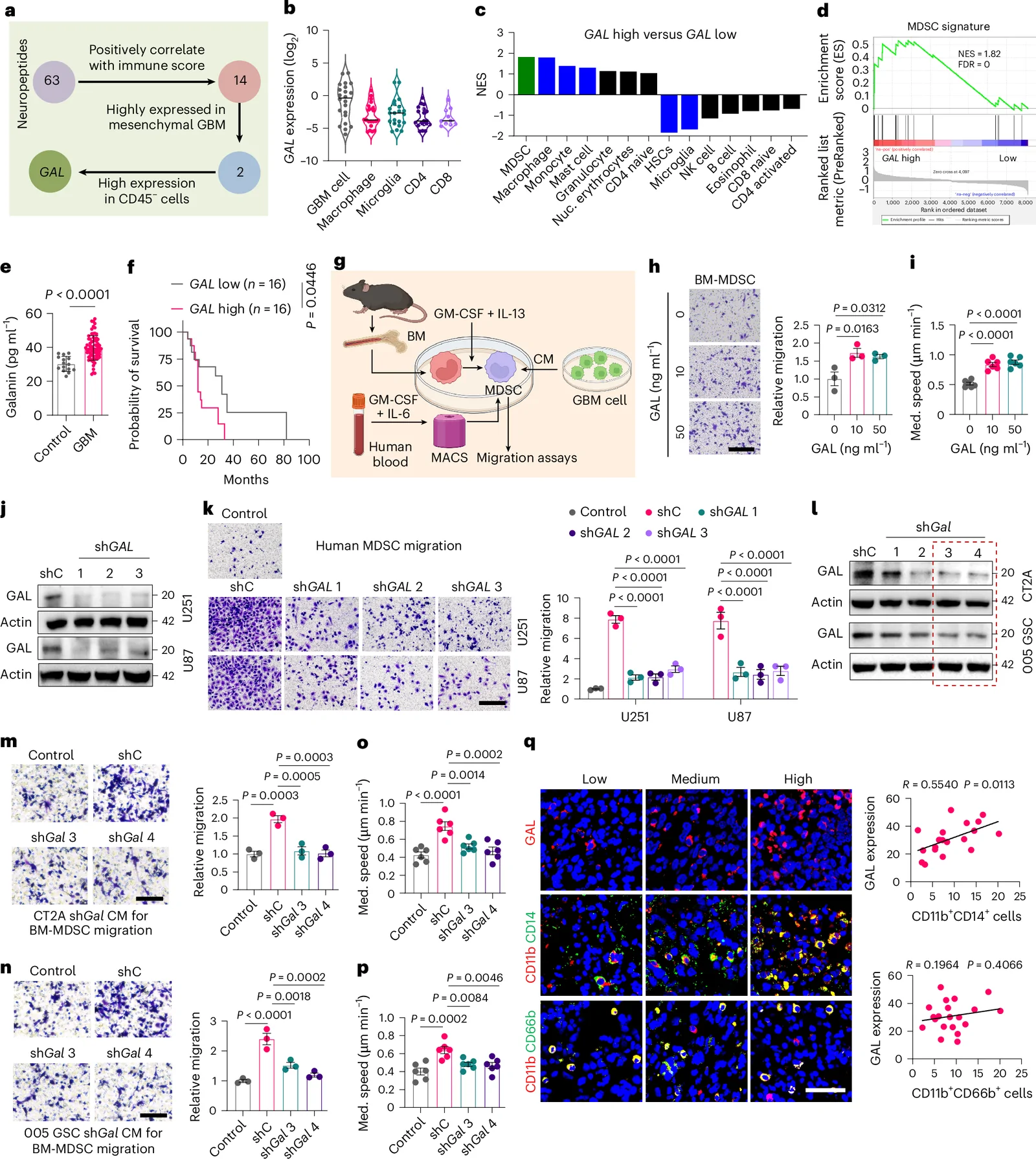
GBM cell-derived GAL facilitates MDSC infiltration. **a**, Strategy for the identification of *GAL* that encodes secreted protein, correlates positively with the immune score and is highly expressed in immune-score-enriched mesenchymal GBM tumors and in CD45^−^ cancer cells of tumors from individuals with GBM. **b**, Violin plot showing the expression pattern of *GAL* in CD45^−^GBM cells (*n* = 22) and tumor-associated immune cells including macrophages (*n* = 21), microglia (*n* = 21), CD4^+^ T cells (*n* = 19) and CD8^+^ T cells (*n* = 10) isolated from human GBM tumors based on the brain TIME dataset. The black lines at the center of each group indicate median expression level and dots represent individual samples. **c**, GSEA analysis for distinct types of immune cells in *GAL*-high (*n* = 122) and *GAL*-low tumors (*n* = 122) from TCGA GBM dataset. The highlighted green and blue bars indicate signatures that are significantly enriched (false discovery rate (FDR) < 0.25). **d**, GSEA for MDSC signature in *GAL*-high (*n* = 122) versus *GAL*-low tumors (*n* = 122) from TCGA GBM dataset. Normalized enrichment scores (NESs) and FDR values are shown. **e**, ELISA for GAL in the plasma from healthy controls (*n* = 15) and individuals with GBM (*n* = 70). **f**, Overall survival of individuals with GBM with high and low GAL plasma concentration. **g**, Diagram illustrating the workflow for MDSC migration assays. Mouse BM-MDSCs and human blood-derived MDSCs were treated with the CM from GBM cells and subjected to transwell migration assay or live-cell trajectory analysis. **h**, Representative and quantification of relative migration of BM-MDSCs following stimulation with GAL protein at indicated concentrations (*n* = 3 independent samples). **i**, Quantification of incucyte live-cell migration of BM-MDSCs following stimulation with GAL protein at indicated concentrations (*n* = 6 independent samples). **j**, Immunoblots for GAL in cell lysates of U87 and U251 cells expressing shRNA control (shC) and *GAL* shRNA (sh*GAL*) (*n* = 3 independent experiments). **k**, Representative and quantification of relative migration of human MDSCs following stimulation with the CM from U87 and U251 cells expressing shC or sh*GAL* (*n* = 3 independent samples). **l**, Immunoblots for GAL in cell lysates of CT2A and 005 GSCs expressing shC and sh*Gal* (*n* = 3 independent experiments). **m**,**n**, Representative and quantification of relative migration of BM-MDSCs following stimulation with the CM from CT2A cells (**m**) or 005 GSCs (**n**) expressing shC and sh*Gal* (*n* = 3 independent samples). **o**,**p**, Quantification of incucyte live-cell migration of BM-MDSCs following stimulation with the CM from CT2A cells (**o**) or 005 GSCs (**p**) expressing shC and sh*Gal* (*n* = 6 independent samples). **q**, Representative immunofluorescence images show the level of GAL, CD11b^+^CD14^+^ cells and CD11b^+^CD66b^+^ cells and quantifications show the correlations between GAL and CD11b^+^CD14^+^ cells and CD11b^+^CD66b^+^ cells in human GBM tumor samples (*n* = 20). *R* and *P* values were determined by Pearson correlation. Data from multiple replicates are presented as the mean ± s.e.m. Statistical analysis was conducted using a two-sided Student’s *t*-test (**e**), one-way ANOVA (**h**,**i**,**k**,**m**–**p**), two-sided Pearson correlation test (**q**) or log-rank test (**f**). nuc., nucleated; HSCs, hematopoietic stem cells; MACS, magnetic-activated cell sorting; Med., median. Scale bars, 200 μm (**h**,**k**), 50 μm (**m**,**n**,**q**). Source data

**Extended Data Fig. 1:**
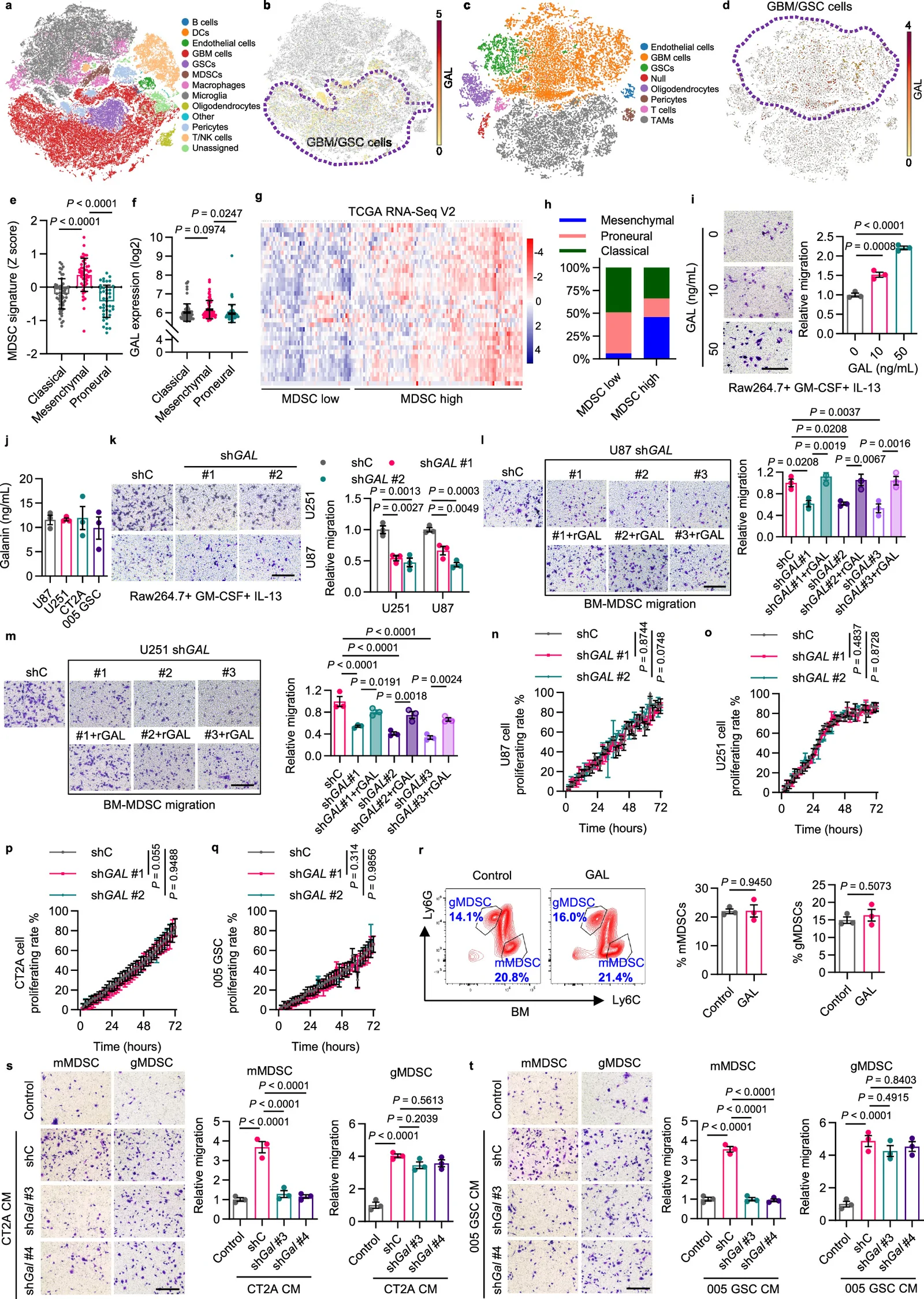
GBM cell-derived GAL promotes MDSC migration. **a**, T-distributed stochastic neighbor embedding (t-SNE) dimensional reduction of cancer cells and immune cells from GBM tumor samples (n = 16) based on single-cell RNA sequencing (scRNA-Seq) dataset (GSE182109). **b**, Pattern representing single-cell gene expression of *GAL* in GBM/GSC cells from GBM tumor samples (n = 16) based on scRNA-Seq dataset (GSE182109). **c**, T-SNE dimensional reduction of cancer cells and immune cells from GBM tumor samples (n = 7) based on scRNA-Seq dataset (GSE135045). **d**, Pattern representing single-cell gene expression of *GAL* in GBM/GSC cells from GBM tumor samples (n = 7) based on scRNA-Seq dataset (GSE135045). **e**, The expression of MDSC signature in classical (n = 54), mesenchymal (n = 46), and proneural GBM tumors (n = 41). **f**, GAL expressions in classical (n = 75), mesenchymal (n = 64), and proneural (n = 64) GBM tumors. **g**, Clustering of TCGA GBM tumors into MDSC-high (n = 51) and MDSC-low groups (n = 97) using a 38-gene MDSC signature. **h**, The percentages of patients with classical, mesenchymal, and proneural GBM tumors in the MDSC-high (n = 51) and MDSC-low (n = 97) groups. **i**, Representative and quantification of relative migration of Raw264.7-derived MDSCs following stimulation with GAL protein at indicated concentrations. Scale bar, 200 μm. n = 3 independent samples. **j**, GAL concentration in the conditioned media (CM) from U87, U251, CT2A, and 005 GSC cells. n = 3 independent samples. **k**, Representative and quantification of relative migration of Raw264.7-derived MDSCs following stimulation with the CM from U251 or U87 cells expressing shRNA control (shC) and *GAL* shRNAs (sh*GAL*). Scale bar, 200 μm. n = 3 independent samples. **l,m**, Representative and quantification of relative migration of BM-MDSCs following stimulation with the CM from control and GAL-depleted U87 cells (**l**) or U251 cells (**m**) in combination with or without GAL protein. Scale bars, 200 μm. n = 3 independent samples. **n-q**, Proliferation curves of U87 (**n**), U251(**o**), CT2A (**p**), and 005 GSC (**q**) cells expressing shC and sh*GAL*. GBM cell proliferation was recorded and analyzed using the Incucyte imaging system for 72 h. n = 6 independent samples. **r**, Representative images and quantification of flow cytometry for the percentage of mMDSCs and gMDSCs in bone marrow (BM) cells treated with MDSC differentiation inducers (GM-CSF and IL-13) combining with or without with GAL. n = 3 independent samples. **s,t**, Representative and quantification of relative migration of BM-mMDSCs and BM-gMDSCs following stimulation with the CM from CT2A cells (**s**) or 005 GSCs (**t**) expressing shC and sh*Gal*. Scale bars, 200 μm. n = 3 independent samples. Data from multiple replicates are presented as mean ± s.e.m. Student’s t-test, two-sided (**r**), one-way ANOVA test (**e, f, i, k-m, s, t**), two-way ANOVA test (**n-q**). Source data

Tumor–immune cell symbiosis is critical for tumor progression and immunosuppression in GBM^15,23^. To identify the specific CNS factors in the GBM TME governing this symbiosis, we performed unbiased analyses by (1) focusing on 63 neuropeptide genes encoding secreted proteins using the Human Gene Nomenclature (HGNC) database; (2) identifying the subset of genes whose expression positively correlates with the immune score^24^ in The Cancer Genome Atlas (TCGA) GBM tumors; (3) determining the subset of genes that are highly expressed in immune-enriched mesenchymal GBM tumors^25^; and (4) identifying the subset of genes highly expressed in CD45^−^ GBM cells using the tumor immune microenvironment (TIME) dataset^26^ and single-cell RNA sequencing (scRNA-seq) datasets^27,28^. Following these analyses, GAL was identified as the key factor (Fig. 1a,b and Extended Data Fig. 1a–d). To identify specific type of immune cells linked to GAL expression, we performed gene set enrichment analysis (GSEA) on validated immune cell signatures^25,29^ in TCGA GBM tumors, showing that high *GAL* expression correlated with significant enrichment of MDSCs and, to a lesser extent, macrophages, monocytes and mast cells (Fig. 1c,d), whereas hematopoietic stem cells and microglia were negatively corrected with *GAL* expression (Fig. 1c). The connection between MDSCs and GAL was reinforced by our analysis in tumors from individuals with GBM showing that higher MSDC signature^30^ and GAL expression in mesenchymal tumors versus proneural and classical tumors (Extended Data Fig. 1e,f). Next, we classified individuals with GBM into MDSC-low and MDSC-high groups on the basis of the MDSC signature^30^ (Extended Data Fig. 1g) and found higher mesenchymal tumors in the MDSC-high group compared to the MDSC-low group (Extended Data Fig. 1h). In addition, plasma GAL levels were higher in individuals with GBM than healthy controls and correlated with poorer overall survival (Fig. 1e,f). Together, these observations support a connection between cancer-cell-derived GAL and MDSCs in GBM.

To investigate the role of GAL in regulating MDSC biology experimentally, we generated MDSCs from mouse BM, Raw264.7 monocytes and human peripheral blood (Fig. 1g). Transwell migration and Incucyte live-cell analyses revealed that MDSC migration was significantly enhanced by the treatment with GAL protein (Fig. 1h,i and Extended Data Fig. 1i). As GAL is a secreted protein, we next investigated whether tumor-derived GAL might promote MDSC infiltration and first confirmed that GAL is abundantly present in the conditioned medium (CM) of GBM cell lines (Extended Data Fig. 1j). Then, we carried out short hairpin RNA (shRNA)-mediated depletion studies in human U87 and U251 GBM cells (Fig. 1j). The CM from *GAL-*depleted GBM cells significantly reduced migration of human MDSCs (Fig. 1k), Raw264.7-derived MDSCs (Extended Data Fig. 1k) and mouse BM-MDSCs (Extended Data Fig. 1l,m). Notably, these effects were rescued by adding GAL protein into the CM of *GAL*-depleted cells (Extended Data Fig. 1l,m). Next, we performed shRNA-mediated depletion of GAL in mouse CT2A cells and 005 GSCs (Fig. 1l) and confirmed that depletion of GAL reduced the migration of BM-MDSCs (Fig. 1m–p). However, depletion of GAL in human (for example, U87 and U251) and mouse (for example, CT2A and 005 GSC) GBM cells did not affect their proliferation (Extended Data Fig. 1n–q). Adding GAL protein into BM cells during their differentiation did not affect the relative proportion of mMDSCs and gMDSCs (Extended Data Fig. 1r). To explore the clinical importance of GAL in regulating MDSC infiltration, we performed immunofluorescence staining for GAL and MDSC markers in tumors from individuals with GBM, showing that GAL expression correlated positively with CD11b^+^CD14^+^ monocytic myeloid cells (for example, mMDSCs) but not with CD11b^+^CD66b^+^ granulocytic myeloid cells (for example, gMDSCs) (Fig. 1q). Consistent with these clinical findings, transwell migration assays showed greatly reduced migration of mMDSCs but not gMDSCs with CM from the *GAL*-depleted CT2A and 005 GSCs compared with CM from control cells (Extended Data Fig. 1r,s). Together, these findings demonstrate that GBM cells can secrete GAL to promote mMDSC infiltration and affect GBM biology.

### GAL inhibition suppresses mMDSC infiltration and tumor progression in immunocompetent GBM mouse models

**Fig. 2:**
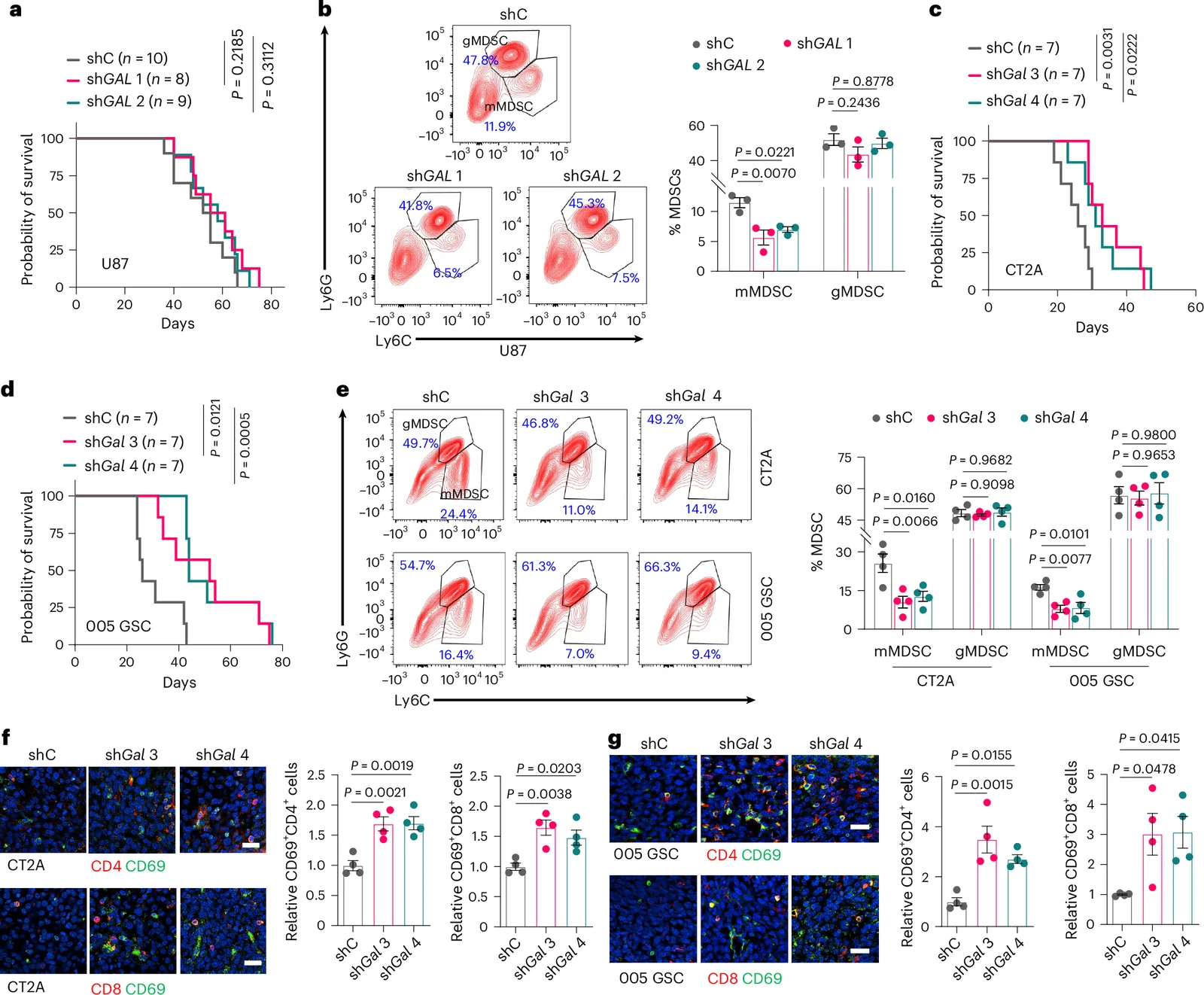
*GAL* depletion impairs tumor growth, reduces MDSC infiltration and activates T cells in immunocompetent GBM mouse models. **a**, Survival curves of nude mice implanted with 2 × 10^4^ U87 cells expressing shC and sh*GAL*. **b**, Representative images and quantification of flow cytometry for the percentage of mMDSCs and gMDSCs in size-matched U87 tumors expressing shC and sh*GAL* (*n* = 3 independent samples). **c**,**d**, Survival curves of C57BL/6 mice implanted with 2 × 10^4^ CT2A (**c**) or 2 × 10^5^ 005 GSC (**d**) cells expressing shC and sh*Gal*. **e**, Representative images and quantification of flow cytometry for the percentage of mMDSCs and gMDSCs in size-matched CT2A and 005 GSC tumors expressing shC and sh*Gal* (*n* = 3 independent samples). **f**,**g**, Coimmunofluorescence and quantification of relative CD4^+^CD69^+^ T cells and CD8^+^CD69^+^ T cells in CT2A (**f**) or 005 GSC (**g**) tumors expressing shC and sh*Gal* (*n* = 4 independent samples). Data from multiple replicates are presented as the mean ± s.e.m. Statistical analysis was conducted using a one-way ANOVA (**b**,**e**–**g**) or log-rank test (**a**,**c**,**d**). Scale bars, 25 μm (**f**,**g**). Source data

**Extended Data Fig. 2:**
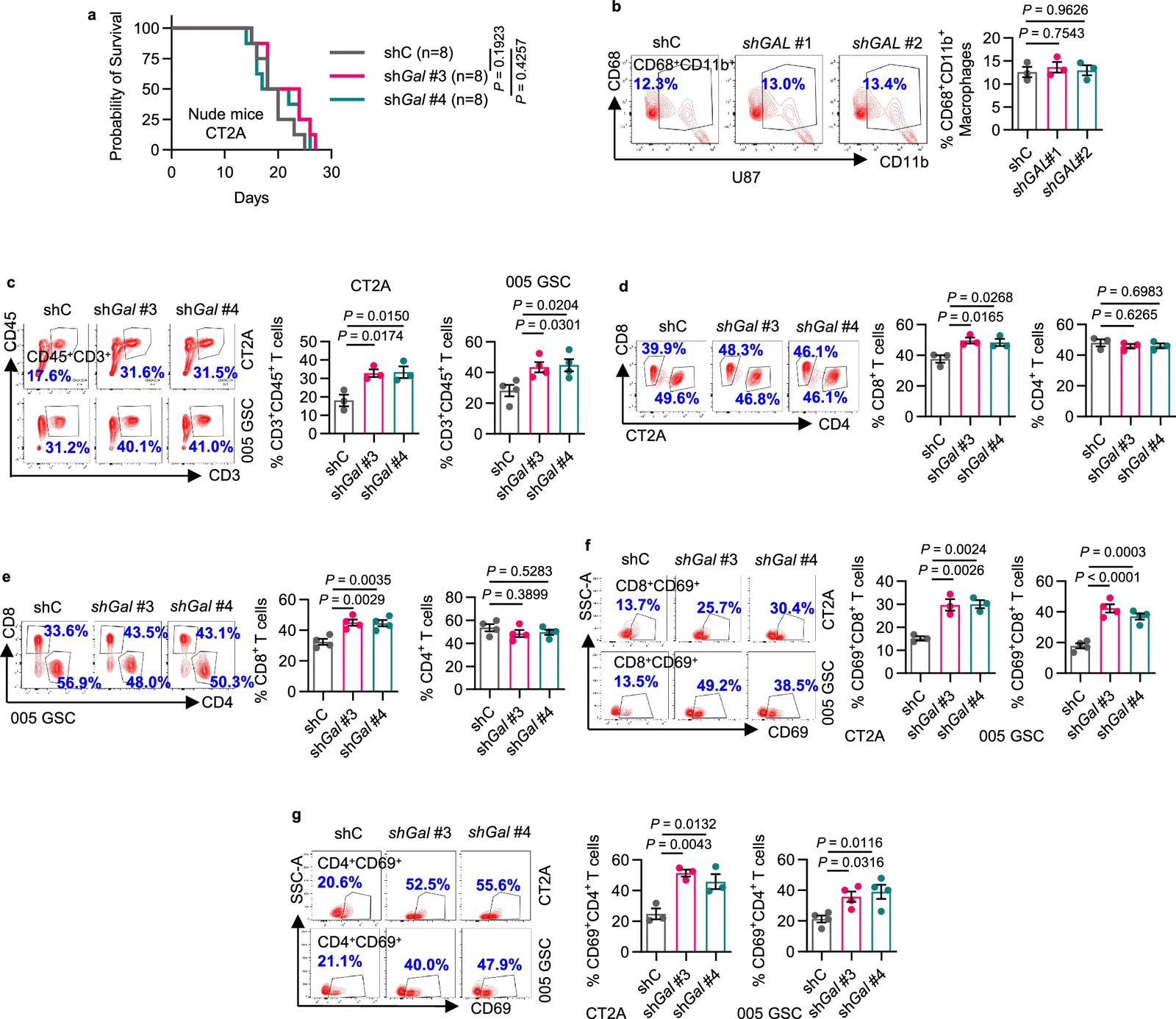
Inhibition of GAL activates T cells but does not affect macrophages in GBM mouse models. **a**, Survival curves of nude mice implanted with 2 × 10^4^ CT2A cells expressing shRNA control (shC) and *GAL* shRNA (sh*GAL*). **b**, Representative images and quantification of flow cytometry for the percentage of CD45^+^CD11b^+^CD68^+^ macrophages in size-matched U87 tumors expressing shC and sh*GAL*. n = 3 independent samples. **c**, Representative images and quantification of flow cytometry for the percentage of splenic CD45^+^CD3^+^ T cells in CT2A and 005 GSC tumors expressing shC and sh*Gal*. n = 3 and 4 independent samples for CT2A and 005 GSC tumors, respectively. **d,e**, Representative images and quantification of flow cytometry for the percentage of splenic CD45^+^CD3^+^CD8^+^ T cells and CD45^+^CD3^+^CD4^+^ T cells in CT2A (**d**, n = 3 independent samples) and 005 GSC (**e**, n = 4 independent samples) tumors expressing shC and sh*Gal*. **f,g**, Representative images and quantification of flow cytometry for the percentage of splenic CD45^+^CD3^+^CD8^+^CD69^+^ T cells (n = 3 independent samples) and CD45^+^CD3^+^CD4^+^CD69^+^ T cells (n = 4 independent samples) in CT2A (**f**) and 005 GSC (**g**) tumors expressing shC and sh*Gal*. Data from multiple replicates are presented as mean ± s.e.m. One-way ANOVA test (**b** and **c-g**), log-rank test (**a**). Source data

Given the role of mMDSCs in tumor progression, we evaluated the antitumor efficacy of GAL inhibition in GBM mouse models. Surprisingly, we found that *GAL* depletion did not extend the survival of nude mice implanted with U87 (Fig. 2a) or CT2A (Extended Data Fig. 2a) cells. Despite lacking survival benefit, *GAL* depletion significantly inhibited mMDSC infiltration but did not affect the infiltration of gMDSCs and macrophages (Fig. 2b and Extended Data Fig. 2b). These findings led us to hypothesize that the immune system is essential for GAL-induced GBM progression. To explore this, we implanted control and *GAL*-depleted CT2A and 005 GSC cells into the brains of immunocompetent C57BL/6 mice. In both models, *GAL* depletion significantly extended survival (Fig. 2c,d) and decreased mMDSC infiltration but did not affect gMDSCs (Fig. 2e). Flow cytometry analysis demonstrated that *GAL* depletion significantly upregulated the percentage of splenic T cells (CD45^+^CD3^+^), CD8 T cells (CD45^+^CD3^+^CD8^+^), activated CD8 T cells (CD45^+^CD3^+^CD8^+^CD69^+^) and activated CD4 T cells (CD45^+^CD3^+^CD4^+^CD69^+^) from CT2A and 005 GSC tumor-bearing mice (Extended Data Fig. 2c–g). However, CD4 T cells (CD45^+^CD3^+^CD4^+^) were not affected upon *GAL* depletion in both models (Extended Data Fig. 2d,e). Moreover, immunofluorescence analysis showed that depletion of GAL in CT2A and 005 GSC tumors significantly increased intratumoral activated CD4 (CD69^+^CD4^+^) and activated CD8 (CD69^+^CD8^+^) T cells (Fig. 2f,g). Together, these results demonstrate that inhibition of GAL could suppress GBM tumor progression in immunocompetent GBM mouse models.

### GALR3 mediates GAL-induced mMDSC infiltration in GBM

**Fig. 3:**
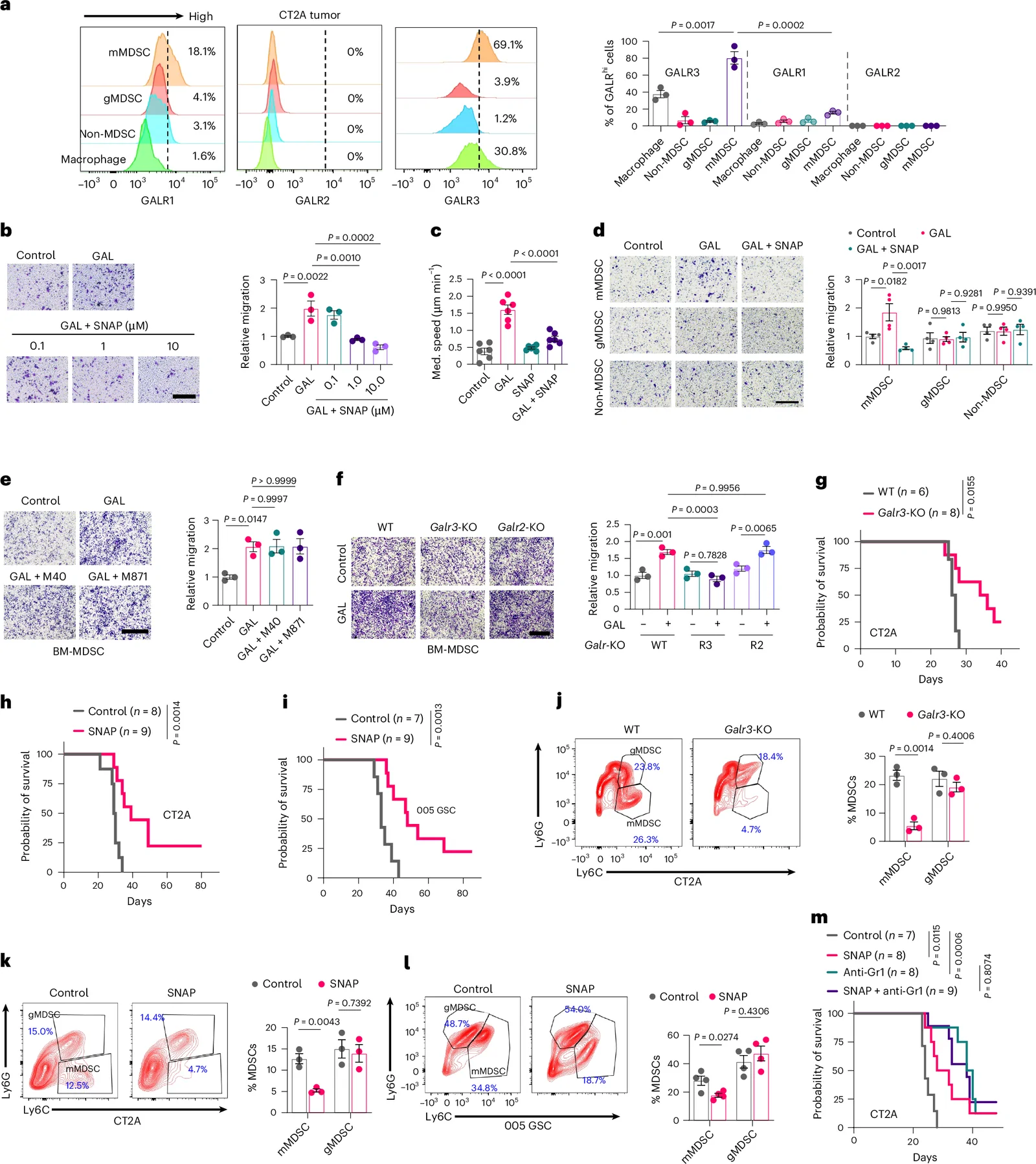
GALR3 is essential for GAL-induced MDSC infiltration and tumor progression in GBM. **a**, Representative images and quantification of flow cytometry for the percentage of GALR high expressing cells (GALR^hi^) in myeloid cell subpopulations, including mMDSCs (CD11b^+^CD68^−^Ly6C^high^Ly6G^low^), gMDSCs (CD11b^+^CD68^−^Ly6C^low^Ly6G^high^), macrophages (CD11b^+^CD68^+^) and non-MDSC myeloid cells (CD11b^+^CD68^−^Ly6C^−^Ly6G^−^), isolated from CT2A tumors (*n* = 3 independent samples). **b**, Representative and quantification of relative migration of BM-MDSCs following stimulation with GAL (10 ng ml^−1^) in the presence or absence of GALR3 antagonist SNAP at indicated concentrations (*n* = 3 independent samples). **c**, Quantification of incucyte live-cell migration of BM-MDSCs following stimulation with GAL in the presence or absence of SNAP (1 μM) (*n* = 6 independent samples). **d**, Representative and quantification of relative migration of FACS-isolated CD11b^+^CD68^−^Ly6C^high^Ly6G^low^ mMDSCs, CD11b^+^CD68^−^Ly6C^low^Ly6G^high^ gMDSCs and CD11b^+^CD68^−^Ly6C^−^Ly6G^−^ non-MDSCs following stimulation with GAL in the presence or absence of SNAP (*n* = 4 independent samples). **e**, Representative and quantification of relative migration of BM-MDSCs following stimulation with GAL in the presence or absence of GALR1/2 antagonist M40 (1 μM) and M871 (1 μM) (*n* = 3 independent samples). **f**, Representative and quantification of relative migration of BM-MDSCs (isolated from WT, *Galr3*-KO and *Galr2*-KO mice) following stimulation with GAL (*n* = 3 independent samples). **g**, Survival curves of WT and *Galr3*-KO mice implanted with 2 × 10^4^ CT2A cells. **h**, Survival curves of C57BL/6 mice implanted with 2 × 10^4^ CT2A cells and treated with SNAP (30 mg kg^−1^, i.p., daily) starting on day 7 for ten doses. **i**, Survival curves of C57BL/6 mice implanted with 2 × 10^5^ 005 GSCs and treated with SNAP (30 mg kg^−1^, i.p., daily) starting on day 7 for ten doses. **j**, Representative images and quantification of flow cytometry for the percentage of mMDSCs and gMDSCs in size-matched CT2A tumors from WT and *Galr3*-KO mice (*n* = 3 independent samples). **k**,**l**, Representative images and quantification of flow cytometry for the percentage of mMDSCs and gMDSCs in size-matched CT2A (**k**) or 005 GSC (**l**) tumors from C57BL/6 mice that received SNAP treatment (*n* = 3 (**k**) or 4 (**l**) independent samples). **m**, Survival curves of C57BL/6 mice implanted with 2 × 10^4^ CT2A cells. Mice received SNAP treatment in combination with or without anti-Gr1 (10 mg kg^−1^, i.p., every other day, starting on day 7 for eight doses). Data from multiple replicates are presented as the mean ± s.e.m. Statistical analysis was conducted using a two-sided Student’s *t*-test (**j**–**l**), one-way ANOVA (**a**–**f**) or log-rank test (**g**–**i**,**m**). Scale bars, 200 μm (**b**,**d**–**f**). Source data

**Extended Data Fig. 3:**
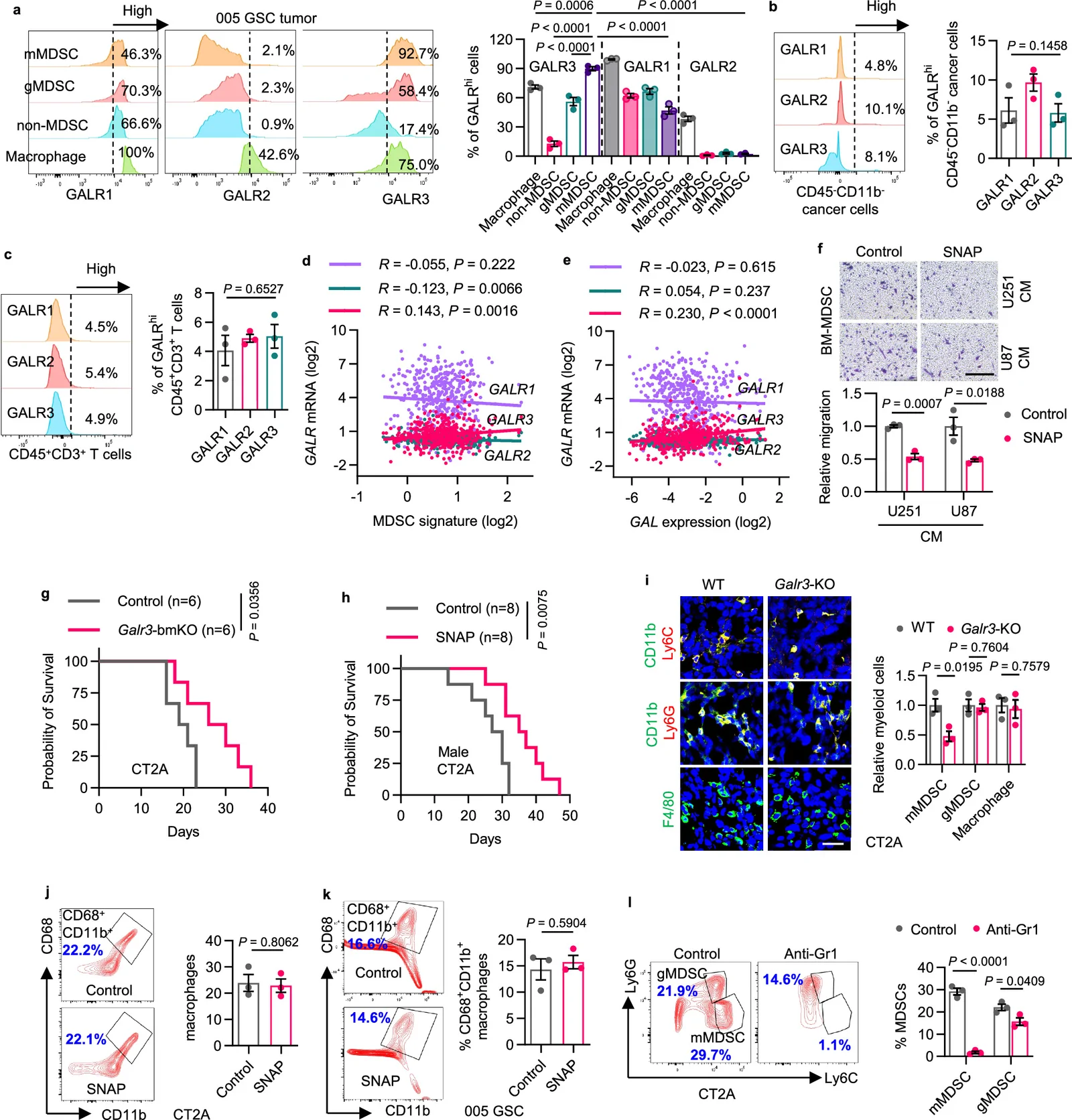
GALR3 inhibition reduces MDSCs but does not affect macrophages in GBM. **a**, Representative images and quantification of flow cytometry for the percentage of GALR high expressing cells (GALR^hi^) in myeloid cell subpopulations, including mMDSCs (CD11b^+^CD68^−^Ly6C^high^Ly6G^low^), gMDSCs (CD11b^+^CD68^−^Ly6C^low^Ly6G^high^), macrophages (CD11b^+^CD68^+^), and non-MDSC myeloid cells (CD11b^+^CD68^−^Ly6C^−^Ly6G^−^), isolated from 005 GSC tumors. n = 3 independent samples. **b**, Representative images and quantification of flow cytometry for the percentage of GALR^hi^ in CD45^−^CD11b^−^ cancer cells. n = 3 independent samples. **c**, Representative images and quantification of flow cytometry for the percentage of GALR^hi^ in CD45^+^CD3^+^ T cells. n = 3 independent samples. **d**, Correlation between MDSC signature and GALR (*GALR1*, *GALR2*, and *GALR3*) in TCGA GBM tumors (n = 489). *R* and *P* values are shown. **e**, Correlation between *GAL* and GALR (*GALR1*, *GALR2*, and *GALR3*) in TCGA GBM tumors (n = 489). *R* and *P* values are shown. **f**, Representative and quantification of relative migration of BM-MDSCs following stimulation with the CM from U251 and U87 cells in the presence or absence of SNAP (1 μM). Scale bar, 200 μm. n = 3 independent samples. **g**, Survival curves of CT2A tumor-bearing chimeric mice with adoptively transferred bone marrow from WT or *Galr3*-KO mice. **h**, Survival curves of male C57BL/6 mice implanted with 2 × 10^4^ CT2A cells and treated with SNAP (30 mg/kg, i.p., daily) starting on day 7 for 10 doses. **i**, Representative immunofluorescence images and quantification for CD11b^+^Ly6C^+^ mMDSCs, CD11b^+^Ly6G^+^ gMDSCs, and F4/80^+^ macrophages in size-matched CT2A tumors from WT and *Galr3*-KO mice. Scale bar, 25 μm. n = 3 independent samples. **j,k**, Representative images and quantification of flow cytometry for the percentage of CD11b^+^CD68^+^ macrophages in size-matched CT2A (**j**) or 005 GSC (**k**) tumors from C57BL/6 mice treated with or without SNAP. n = 3 independent samples. **l**, Representative images and quantification of flow cytometry for the percentage of mMDSCs and gMDSCs in size-matched CT2A tumors. Mice were treated with Anti-Gr1 (10 mg/kg, i.p., every other day, starting on day 7 for 8 doses). n = 3 independent samples. Data from multiple replicates are presented as mean ± s.e.m. Student’s t-test, two-sided (**f, i-l**), one-way ANOVA test (**a-c**), Pearson correlation, two-sided (**d** and **e**), log-rank test (**g** and **h**). Source data

Previous studies have demonstrated that GAL influences various physiological and pathological processes through its interaction with GALR1, GALR2 and GALR3 (ref. ^31^). To determine which receptor is responsible for GAL-induced MDSC infiltration, we profiled the expression pattern of these GAL receptors in myeloid cell subpopulations, T cells and cancer cells of CT2A and 005 GSC tumors. We found that GALR3 was highly expressed in CD11b^+^CD68^−^Ly6C^high^Ly6G^low^ mMDSCs from both models (Fig. 3a and Extended Data Fig. 3a). In contrast, GALR1 and GALR2 exhibited variable expression levels between the two models (Fig. 3a and Extended Data Fig. 3a). Additionally, these GALRs were not expressed in cancer cells and T cells within CT2A tumors (Extended Data Fig. 3b,c). In line with the experimental approaches, we found that *GALR3* but not *GALR1* and *GALR2* correlated with the MDSC signature and *GAL* expression in GBM TCGA tumors (Extended Data Fig. 3d,e). Transwell migration and Incucyte live-cell analyses showed that nonpeptidergic GALR3 selective antagonist SNAP-37889 (SNAP) suppressed GAL-mediated MDSC migration (Fig. 3b,c). To assess the MDSC subtype selectivity, we performed fluorescence-activated cell sorting (FACS) on mouse BM-MDSCs before conducting the migration assay. GAL significantly increased the migration of mMDSCs (CD11b^+^CD68^−^Ly6C^high^Ly6G^low^) but not gMDSCs (CD11b^+^CD68^−^Ly6C^low^Ly6G^high^) and non-MDSCs (CD11b^+^CD68^−^Ly6C^−^Ly6G^−^) (Fig. 3d). Moreover, the effect of GAL on mMDSC migration was negated by SNAP (Fig. 3d). Conversely, GALR1/2 antagonists M40 and M871 were ineffective in reversing GAL-induced MDSC migration (Fig. 3e). In addition, inhibition of GALR3 with SNAP abolished the upregulation of MDSC migration induced by the CM from U251 and U87 cells (Extended Data Fig. 3f). Genetically, MDSCs isolated from *Galr3*-KO (knockout) but not *Galr2*-KO mice showed impaired migration ability in response to CT2A CM (Fig. 3f). Together, these findings suggest that GAL triggers mMDSC migration specifically through GALR3 in vitro.

**Extended Data Fig. 4:**
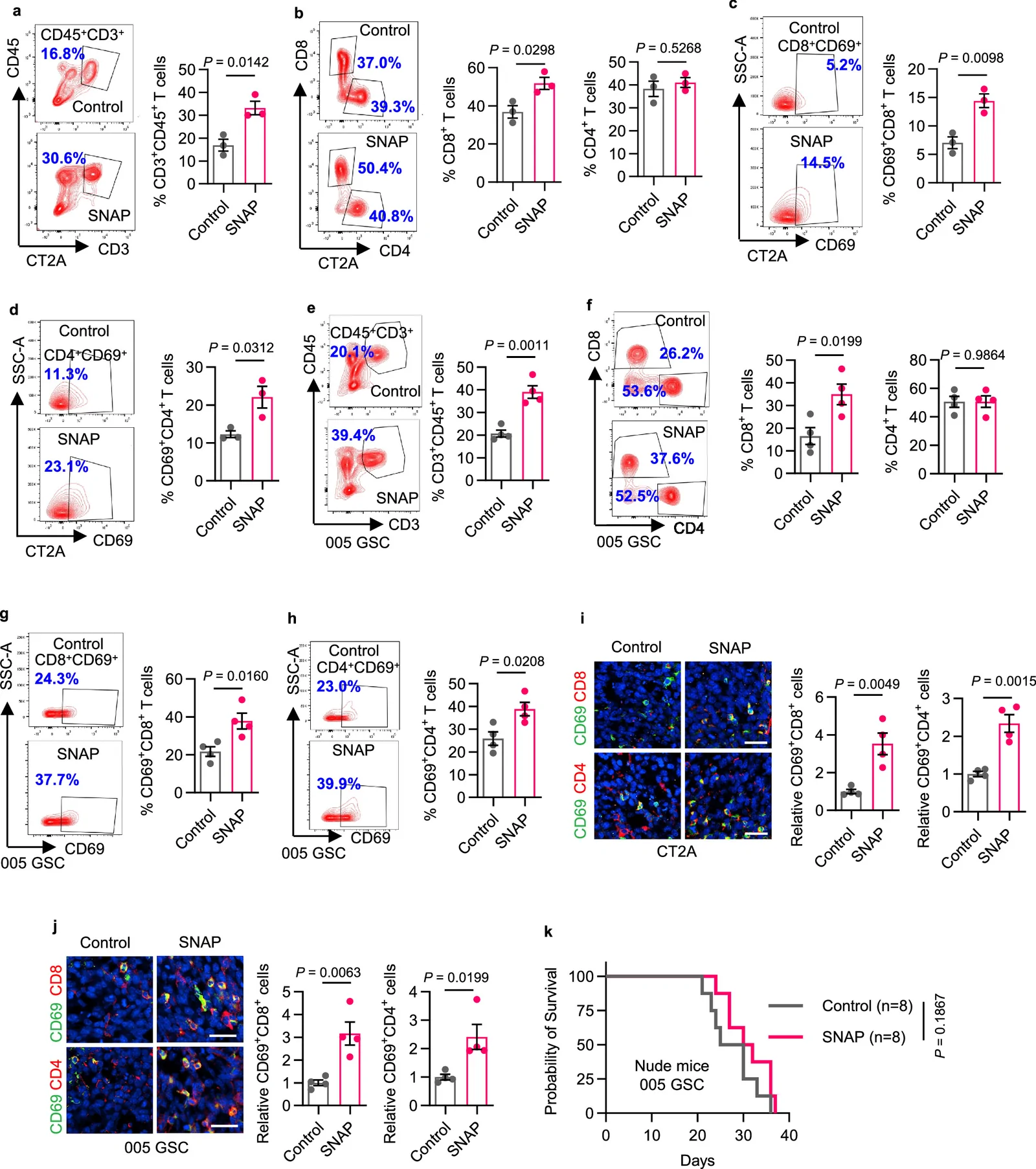
GALR3 inhibition activates T cells in GBM. **a-d**, Representative images and quantification of flow cytometry for the percentage of splenic CD45^+^CD3^+^ T cells (**a**), CD45^+^CD3^+^CD8^+^/CD4^+^ T cells (**b**), CD45^+^CD3^+^CD8^+^CD69^+^ T cells (**c**), and CD45^+^CD3^+^CD4^+^CD69^+^ T cells (**d**) in CT2A tumor-bearing C57BL/6 mice treated with or without SNAP (30 mg/kg, i.p., daily, starting on day 7 for 10 doses). n = 3 independent samples. **e-h**, Representative images and quantification of flow cytometry for the percentage of splenic CD45^+^CD3^+^ T cells (**e**), CD45^+^CD3^+^CD8^+^/CD4^+^ T cells (**f**), CD45^+^CD3^+^CD8^+^CD69^+^ T cells (**g**), and CD45^+^CD3^+^CD4^+^CD69^+^ T cells (**h**) in 005 GSC tumor-bearing C57BL/6 mice treated with or without SNAP. n = 4 independent samples. **i,j**, Co-immunofluorescence and quantification of relative CD8^+^CD69^+^ T cells and CD4^+^CD69^+^ T cells in tumors from CT2A (**i**) and 005 GSC (**j**)-bearing C57BL/6 mice treated with or without SNAP. Scale bars, 25 μm. n = 4 independent samples. **k**, Survival curves of nude mice implanted with 2 × 10^5^ 005 GSCs and treated with SNAP (30 mg/kg, i.p., daily) starting on day 7 for 10 doses. Data from multiple replicates are presented as mean ± s.e.m. Student’s t-test, two-sided (**a-j**), log-rank test (**k**). Source data

To confirm the role of GALR3 in vivo, we intracranially implanted CT2A cells into wild-type (WT) and *Galr3*-KO mice and found a significant survival extension in *Galr3*-KO mice compared to WT mice (Fig. 3g). Next, we performed BM transplantation experiments by transferring *Galr3*-KO or WT BM cells into irradiated WT mice to obtain *Galr3* BM-specific knockdown (*Galr3*-bmKO) and control mice. As expected, CT2A tumor-bearing *Galr3*-bmKO mice exhibited prolonged survival relative to tumor-bearing control mice (Extended Data Fig. 3g). Similarly, pharmacologic treatment with SNAP significantly extended the survival of CT2A and 005 GSC tumor-bearing female and male mice (Fig. 3h,i and Extended Data Fig. 3h), suggesting that this therapeutic effect is sex independent. Flow cytometry and immunofluorescence analyses demonstrated that genetic and/or pharmacologic inhibition of GALR3 significantly inhibited the infiltration of mMDSCs but not gMDSCs and macrophages (Fig. 3j–l and Extended Data Fig. 3i–k). To confirm that MDSCs were the target of GALR3 inhibition in reducing tumor progression, we compared the antitumor effect of SNAP (inhibiting GALR3) and anti-Gr1 (depleting mMDSCs as shown in Extended Data Fig. 3l) in CT2A tumor-bearing mice. Both agents extended the survival equally and their combination did not offer additional antitumor effect (Fig. 3m). To confirm whether the immune system is involved in this process, we performed flow cytometry analysis showing that SNAP treatment significantly increased splenic total T cells (CD45^+^CD3^+^), CD8 T cells (CD45^+^CD3^+^CD8^+^), activated CD8 T cells (CD45^+^CD3^+^CD8^+^CD69^+^) and activated CD4 T cells (CD45^+^CD3^+^CD4^+^CD69^+^) T cells, whereas it barely affected CD4 T cells (CD45^+^CD3^+^CD4^+^) in CT2A and 005 GSC-bearing mice (Extended Data Fig. 4a–h). Immunofluorescence analysis showed that SNAP-treated CT2A and 005 GSC tumors exhibited significantly more activated CD8 T cells (CD69^+^CD8^+^) and activated CD4 T cells (CD69^+^CD4^+^) compared to control tumors (Extended Data Fig. 4i,j). The immune regulatory effect was further supported by intracranial implantation of 005 GSCs into immunocompromised nude mice, showing that GALR3 inhibition with SNAP did not extend survival (Extended Data Fig. 4k). Together, these findings suggest that the GAL–GALR3 axis is essential for mMDSC infiltration and immunosuppression and support GALR3 as a promising therapeutic target for GBM.

### GAL–GALR3 axis promotes mMDSC migration by upregulating the estrogen signaling

**Fig. 4:**
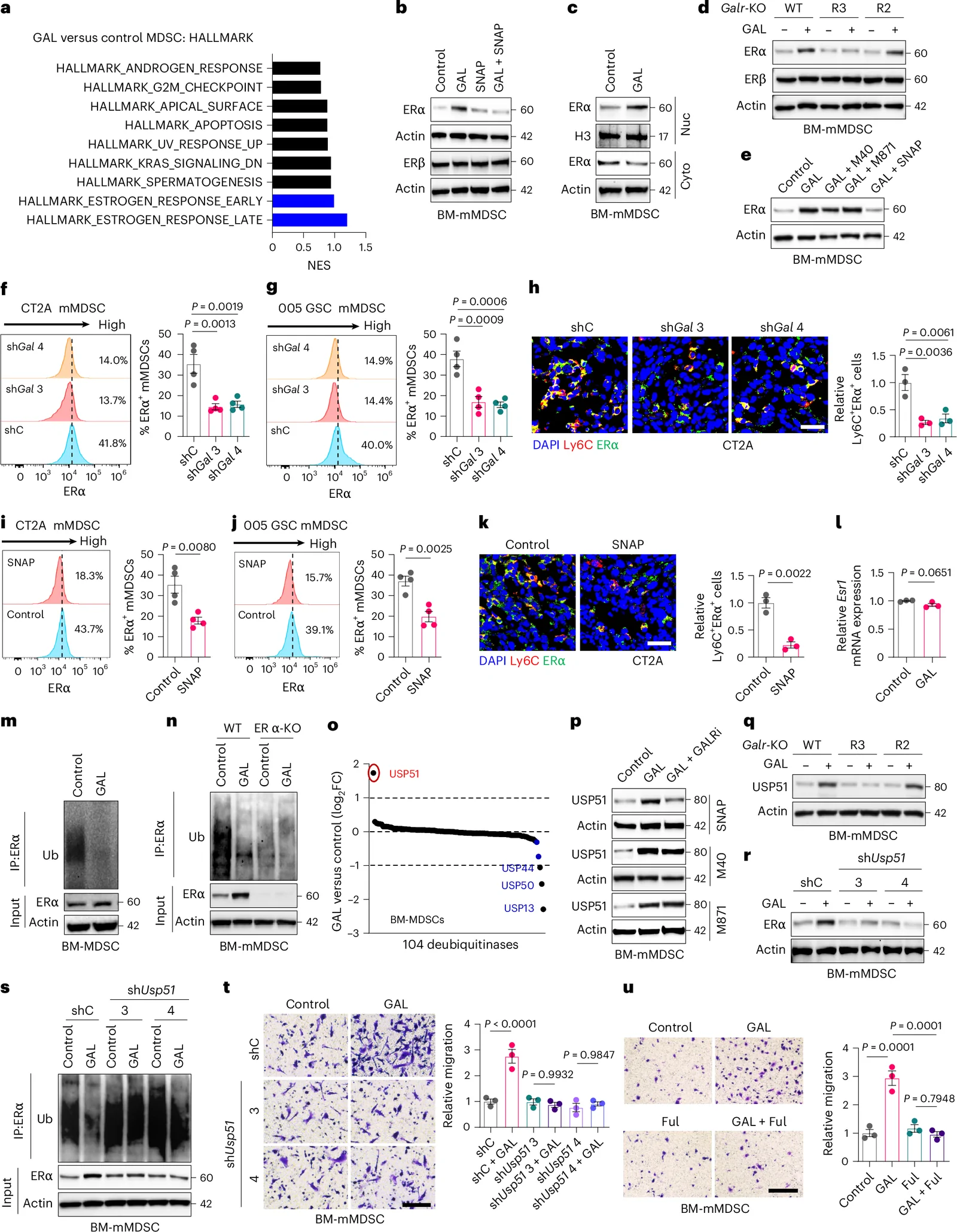
GAL–GALR3 axis promotes MDSC migration through the USP51–ERα signaling pathway. **a**, GSEA analysis shows the top enriched hallmark pathways in GAL (10 ng ml^−1^)-treated BM-MDSCs. The blue-highlighted top two enriched pathways are related to estrogen signaling (*n* = 3 independent samples). **b**, Immunoblots for ERα and ERβ in cell lysates of BM-mMDSCs following stimulation with GAL in the presence or absence of SNAP (1 μM) (*n* = 3 independent experiments). **c**, Immunoblots for ERα in nuclear (Nuc) and cytoplasm (Cyto) of GAL-treated BM-mMDSCs (*n* = 2 independent experiments). **d**, Immunoblots for ERα and ERβ in cell lysates of BM-mMDSC (isolated from WT, *Galr3*-KO and *Galr2*-KO mice) following stimulation with GAL (*n* = 2 independent experiments). **e**, Immunoblots for ERα in cell lysates of BM-mMDSCs following stimulation with GAL in the presence or absence of M40 (1 μM), M871 (1 μM) and SNAP (*n* = 3 independent experiments). **f**,**g**, Representative images and quantification of flow cytometry for the percentage of ERα^+^ mMDSCs in CT2A (**f**) and 005 GSC (**g**) tumors from C57BL/6 mice expressing shC and sh*Gal* (*n* = 4 independent samples). **h**, Representative immunofluorescence images and quantification for Ly6C^+^ERα^+^ mMDSCs in size-matched CT2A tumors expressing shC and sh*Gal* (*n* = 3 independent samples). **i**,**j**, Representative images and quantification of flow cytometry for the percentage of ERα^+^ mMDSCs in CT2A (**i**) and 005 GSC (**j**) tumors from C57BL/6 mice treated with or without SNAP (30 mg kg^−1^, i.p., daily) starting on day 7 for ten doses (*n* = 4 independent samples). **k**, Representative immunofluorescence images and quantification for Ly6C^+^ERα^+^ mMDSCs in size-matched CT2A tumors treated with or without SNAP (*n* = 3 independent samples). **l**, mRNA levels of *Esr1* in BM-MDSCs treated with GAL (*n* = 3 independent samples). **m**, Immunoblot for ERα from total lysates and polymer-ubiquitin from purified ERα protein of BM-MDSCs treated with or without GAL (*n* = 2 independent experiments). **n**, Immunoblot for ERα from total lysates and polymer-ubiquitin from purified ERα protein of BM-mMDSCs isolated from WT or ERα-KO mice treated with or without GAL (*n* = 2 independent experiments). **o**, Fold change (FC) of genes encoding deubiquitinases in GAL-treated BM-MDSCs compared to control. **p**, Immunoblot for USP51 in cell lysates of BM-mMDSCs following stimulation with GAL in the presence or absence of GALR antagonists (SNAP, M40 and M871) (*n* = 3 independent experiments). **q**, Immunoblot for USP51 in cell lysates of BM-mMDSCs (isolated from WT, *Galr3*-KO and *Galr2*-KO mice) treated with GAL (*n* = 2 independent experiments). **r**, Immunoblot for ERα in cell lysates of GAL-treated BM-mMDSCs expressing shC and sh*Usp51* (*n* = 3 independent experiments). **s**, Immunoblot for ERα from total lysates and polymer-ubiquitin from purified ERα protein of BM-mMDSCs expressing shC and sh*Usp51* (*n* = 2 independent experiments). **t**, Representative and quantification of relative migration of shC and sh*Usp51*-tansfected BM-mMDSCs following stimulation with GAL (*n* = 3 independent samples). **u**, Representative and quantification of relative migration of BM-mMDSCs following stimulation with GAL in the presence or absence of Ful (10 μM), where cells were pretreated with Ful for 1 h before the transwell assay, GAL was added to the bottom chamber and Ful was maintained in the upper chamber to ensure continuous ERα inhibition (*n* = 3 independent samples). Data from multiple replicates are presented as the mean ± s.e.m. Statistical analysis was conducted using a two-sided Student’s *t*-test (**i**–**l**) or one-way ANOVA (**f**–**h**,**t**,**u**). Scale bars, 25 μm (**h**,**k**), 100 μm (**t**), 200 μm (**u**). Source data

**Extended Data Fig. 5:**
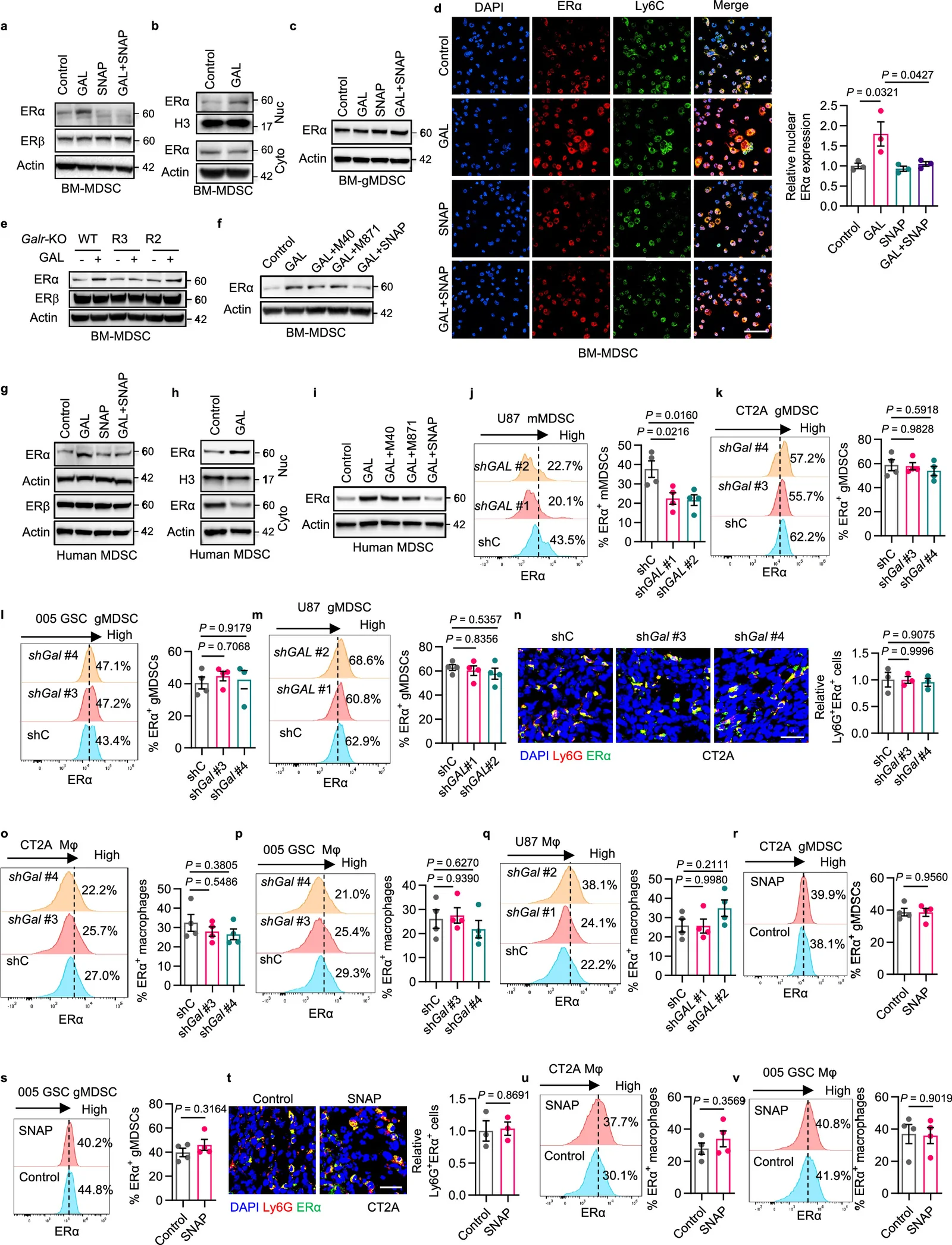
GAL-GALR3 axis regulates ERα expression in mMDSCs, but not in gMDSCs and macrophages, in GBM mouse models. **a**, Immunoblots for ERα and ERβ in cell lysates of BM-MDSCs following stimulation with GAL in the presence or absence of SNAP (1 μM). n = 3 independent experiments. **b**, Immunoblots for ERα in nuclear and cytoplasm of GAL-treated BM-MDSCs. n = 2 independent experiments. **c**, Immunoblots for ERα in cell lysates of BM-gMDSCs following stimulation with GAL (10 ng/mL) in the presence or absence of SNAP (1 μM). n = 3 independent experiments. **d**, Co-immunofluorescence and quantification of relative ERα expression in Ly6C^+^ BM-MDSCs treated with SNAP, GAL, or their combination. Scale bar, 20 μm. n = 3 independent samples. **e**, Immunoblots for ERα and ERβ in cell lysates of BM-MDSC (isolated from WT, *Galr3*-KO, and *Galr2*-KO mice) following stimulation with GAL. n = 2 independent experiments. **f**, Immunoblots for ERα in cell lysates of BM-MDSCs following stimulation with GAL in the presence or absence of M40 (1 μM), M871 (1 μM), and SNAP. n = 3 independent experiments. **g**, Immunoblots for ERα and ERβ in cell lysates of human MDSCs following stimulation with GAL in the presence or absence of SNAP. n = 3 independent experiments. **h**, Immunoblots for ERα in nuclear and cytoplasm of GAL-treated human MDSCs. n = 2 independent experiments. **i**, Immunoblots for ERα in cell lysates of human MDSCs following stimulation with GAL in the presence or absence of M40, M871, and SNAP. n = 3 independent experiments. **j**, Representative images and quantification of flow cytometry for the percentage of ERα^+^ mMDSCs in U87 tumors expressing shRNA control (shC) and *GAL* shRNA (sh*GAL*) from nude mice. n = 4 independent samples. **k,l**, Representative images and quantification of flow cytometry for the percentage of ERα^+^ mMDSCs in CT2A (**k**) and 005 GSC (**l**) tumors expressing shC and sh*Gal* from C57BL/6 mice. n = 4 independent samples. **m**, Representative images and quantification of flow cytometry for the percentage of ERα^+^ gMDSCs in U87 tumors expressing shC and sh*GAL* from nude mice. n = 4 independent samples. **n**, Representative immunofluorescence images and quantification for Ly6G^+^ERα^+^ gMDSCs in size-matched CT2A tumors expressing shC and sh*Gal*. Scale bar, 25 μm. n = 3 independent samples. **o,p**, Representative images and quantification of flow cytometry for the percentage of ERα^+^ macrophages (CD11b^+^CD68^+^) in CT2A (**o**) and 005 GSC (**p**) tumors expressing shC and sh*Gal* from C57BL/6 mice. n = 4 independent samples. **q**, Representative images and quantification of flow cytometry for the percentage of ERα^+^ macrophages in U87 tumors shC and sh*GAL* from nude mice. n = 4 independent samples. **r,s**, Representative images and quantification of flow cytometry for the percentage of ERα^+^ gMDSCs in CT2A (**r**) and 005 GSC (**s**) tumors from C57BL/6 mice treated with or without SNAP (30 mg/kg, i.p., daily) starting on day 7 for 10 doses. n = 4 independent samples. **t**, Representative immunofluorescence images and quantification for Ly6G^+^ERα^+^ gMDSCs in size-matched CT2A tumors treated with or without SNAP. Scale bar, 25 μm. n = 3 independent samples. **u,v**, Representative images and quantification of flow cytometry for the percentage of ERα^+^ macrophages in CT2A (**u**) and 005 GSC (**v**) tumors from C57BL/6 mice treated with or without SNAP. n = 4 independent samples. Data from multiple replicates are presented as mean ± s.e.m. Student’s t-test, two-sided (**r-v**), one-way ANOVA test (**d**, and **j-q**). Source data

To understand how the GAL–GALR3 axis promotes MDSC migration, we conducted RNA-seq profiling on BM-MDSCs treated with and without GAL. The GSEA on hallmark pathways revealed that estrogen signaling signatures emerged as the top pathways influenced by GAL stimulation (Fig. 4a). GAL increased the expression and nuclear translocation of ERα but not ERβ in BM-MDSCs, an effect that was reversed by SNAP treatment (Extended Data Fig. 5a,b). This effect was even more significant in BM-mMDSCs (Fig. 4b,c) but not in BM-gMDSCs (Extended Data Fig. 5c). GAL-induced ERα nuclear translocation was further confirmed by immunofluorescence analysis (Extended Data Fig. 5d). GAL-induced ERα expression was negated in BM-MDSCs, especially BM-mMDSCs, isolated from *Galr3*-KO mice but not *Galr2*-KO mice (Fig. 4d and Extended Data Fig. 5e) and by the treatment with SNAP but not GALR1/2 antagonists M40 and M871 (Fig. 4e and Extended Data Fig. 5f). In addition, the role of the GAL–GALR3 axis in mediating ERα expression and nuclear translocation was validated in human peripheral blood mononuclear cell (PBMC)-derived MDSCs (Extended Data Fig. 5g–i). These findings suggest that ERα is a potential downstream target of the GAL–GALR3 axis in mMDSCs. To confirm it in vivo, we analyzed ERα expression in intratumoral MDSCs and found that genetic depletion of GAL significantly inhibited ERα in mMDSCs (Fig. 4f–h and Extended Data Fig. 5j) but not gMDSCs (Extended Data Fig. 5k–n) and macrophages (Extended Data Fig. 5o–q) from CT2A, 005 GSC and U87 models. Similarly, pharmacologic inhibition of GALR3 with SNAP inhibited ERα expression in mMDSCs (Fig. 4i–k) but not gMDSCs (Extended Data Fig. 5r–t) and macrophages (Extended Data Fig. 5u,v) from CT2A and 005 GSC models. These findings suggest that the GAL–GALR3 axis upregulates ERα signaling specifically in GBM tumor-infiltrating mMDSCs.

**Extended Data Fig. 6:**
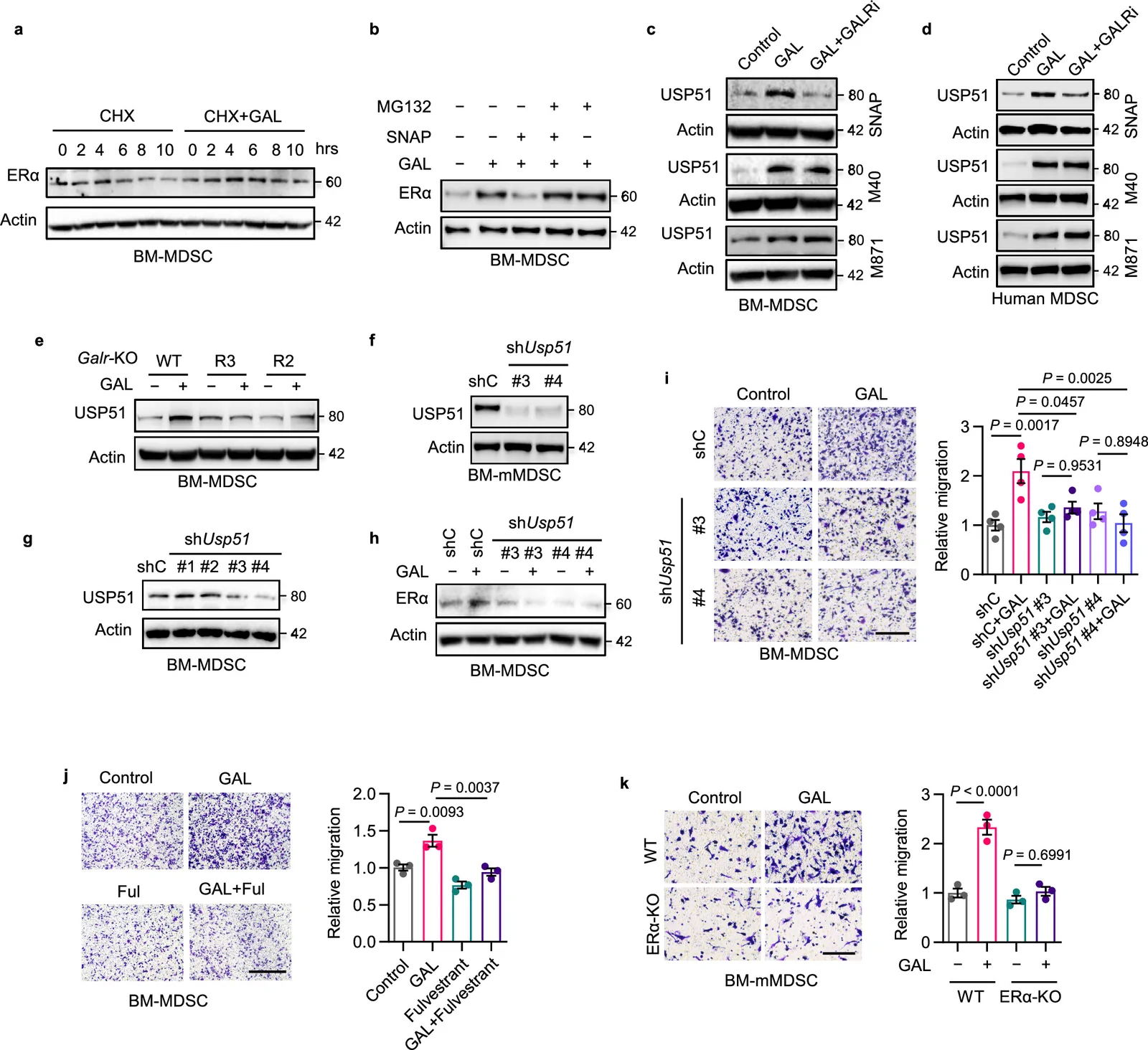
USP51 mediates GAL-induced ERa expression and MDSC migration. **a**, Immunoblots for ERα in cell lysates of GAL-treated BM-MDSCs following incubation of cycloheximide (CHX, 50 μg/mL) at indicated timepoints. n = 3 independent experiments. **b**, Immunoblots for ERα in cell lysates of BM-MDSCs treated with GAL (10 ng/mL), SNAP (1 μM), proteasome inhibitor MG132 (1 μM), or their combinations. n = 2 independent experiments. **c**, Immunoblot for USP51 in cell lysates of BM-MDSCs following stimulation with GAL in the presence or absence of M40 (1 μM), M871 (1 μM), and SNAP. n = 3 independent experiments. **d**, Immunoblot for USP51 in cell lysates of human MDSCs following stimulation with GAL in the presence or absence of GALR antagonists (SNAP, M40, and M871). n = 3 independent experiments. **e**, Immunoblot for USP51 in cell lysates of BM-MDSCs (isolated from WT, *Galr3*-KO, and *Galr2*-KO mice) treated with GAL. n = 2 independent experiments. **f,g**, Immunoblot for USP51 in cell lysates of BM-mMDSCs (**f**) and BM-MDSCs (**g**) expressing shRNA control (shC) and *Usp51* shRNA (sh*Usp51*). n = 3 independent experiments. **h**, Immunoblot for ERα in cell lysates of GAL-treated BM-MDSCs expressing shC and sh*Usp51*. n = 3 independent experiments. **i**, Representative and quantification of relative migration of shC and sh*Usp51* BM-MDSCs following stimulation with GAL. Scale bar, 100 μm. n = 4 independent samples. **j**, Representative and quantification of relative migration of BM-MDSCs following stimulation with GAL in the presence or absence of Fulvestrant (Ful, 10 μM). Scale bar, 200 μm. n = 3 independent samples. **k**, Representative and quantification of relative migration of BM-mMDSCs isolated from WT or ERα-KO mice following stimulation with GAL. Scale bar, 100 μm. n = 3 independent samples. Data from multiple replicates are presented as mean ± s.e.m. One-way ANOVA test (**i-k**). Source data

Next, we performed a reverse transcription (RT)–qPCR experiment in MDSCs showing that GAL did not affect the expression of *Esr1*, the gene encoding ERα (Fig. 4l), suggesting that GAL may regulate ERα degradation rather than transcription. Cycloheximide (CHX) chase and western blotting assays in BM-MDSCs and BM-mMDSCs demonstrated that GAL maintained ERα protein stability (Extended Data Fig. 6a) and suppressed ERα ubiquitination (Fig. 4m,n). Notably, this inhibitory effect of GAL on ERα ubiquitination was abolished in mMDSCs isolated from ERα-KO mice (Fig. 4n). To validate the crucial role of the proteasome system in this process, we treated MDSCs with GAL in the presence or absence of proteasome inhibitor MG132. SNAP abolished GAL-induced ERα upregulation, an effect that was negated by MG132 (Extended Data Fig. 6b). Together, these findings suggest that GAL stabilizes ERα protein by inhibiting ERα ubiquitination. To identify the mediator of GAL-induces deubiquitination of ERα, we performed the profiling of deubiquitinases in MDSCs, with results showing that GAL treatment upregulated *USP51* (Fig. 4o). Western blotting assays confirmed that GAL treatment upregulated USP51 in mouse BM-mMDSCs and BM-MDSCs, as well as human MDSCs, which was abolished by inhibition (using GALR inhibitors) and depletion (isolating BMDMs from *Garl3*-KO and *Garl2*-KO mice) of GALR3 but not GALR1 and GALR2 (Fig. 4p,q and Extended Data Fig. 6c–e). To understand the role of USP51 in mediating GAL-induced ERα expression and MDSC migration, we conducted shRNA-mediated knockdown of *Usp51* in mouse BM-mMDSCs and BM-MDSCs (Extended Data Fig. 6f,g) and found that USP51 depletion abolished the effect of GAL on ERα expression, deubiquitination and migration (Fig. 4r–t and Extended Data Fig. 6h, i). Similarly, inhibition of ERα either through treatment with the ERα inhibitor fulvestrant (Ful), a selective ER degrader that binds, blocks and degrades the ER, leading to complete inhibition of estrogen signaling through the ER^32^, or through genetic deletion of ERα abolished GAL-induced MDSC migration (Fig. 4u and Extended Data Fig. 6j, k). Together, these findings suggest that the GAL–GALR3 axis-induced mMDSC migration is regulated by USP51-directed ERα deubiquitination.

### The GAL–GALR3 axis endows ferroptosis resistance in mMDSCs

**Fig. 5:**
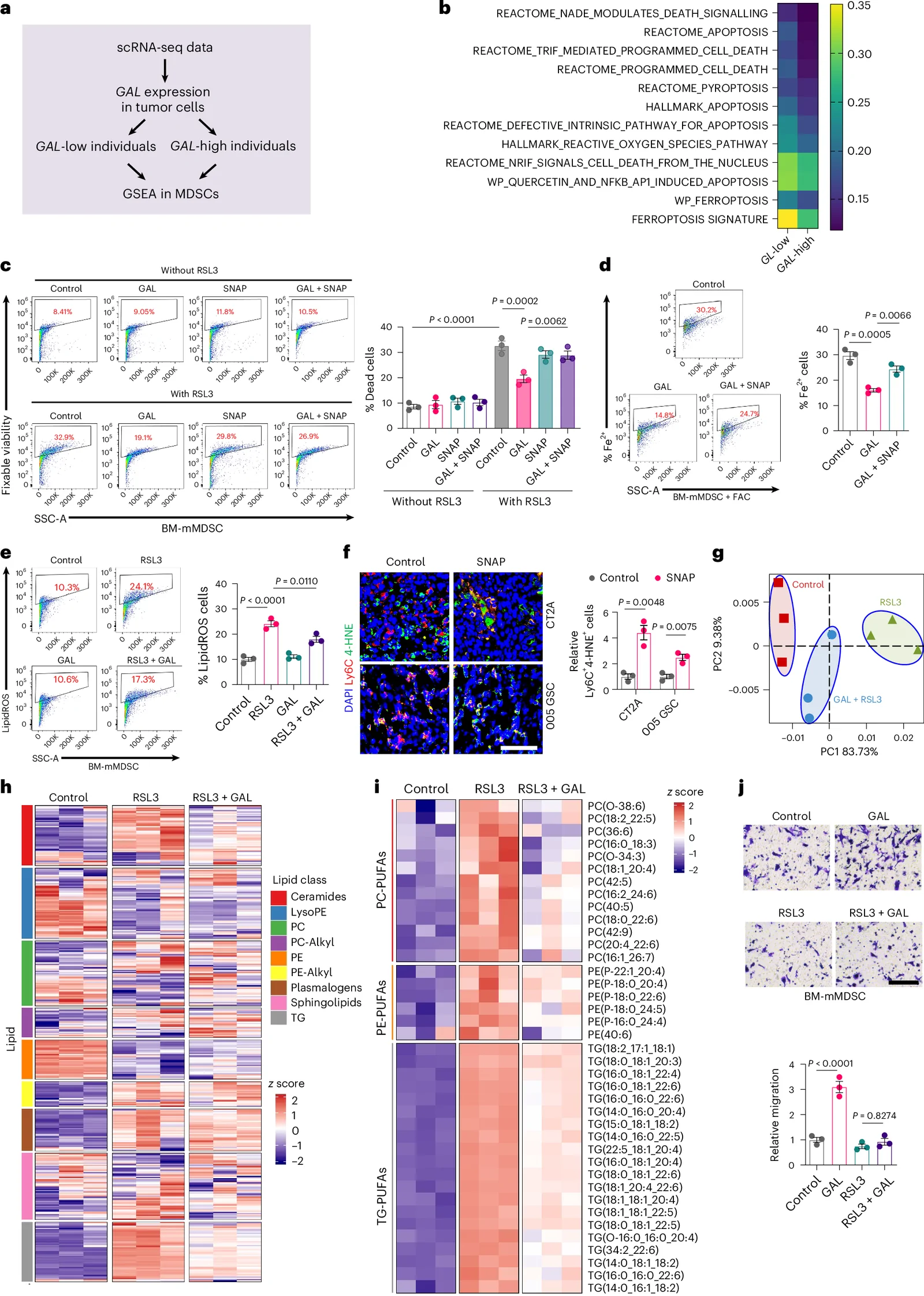
The GAL–GALR3 axis protects MDSCs from ferroptosis. **a**, Strategy for clustering tumors from individuals with GBM into *GAL*-low and *GAL*-high subgroups (*n* = 8 per group) and performing GSEA in MDSCs from these two groups using scRNA-seq data (GSE182109). **b**, Heat map of GSEA for indicated signatures in MDSCs from *GAL*-high and *GAL*-low groups (*n* = 8 per group). The mean AUCell scores are shown. **c**, Representative and quantification of the viability of BM-mMDSC treatment with RSL3 (20 μM) for 16 h in the presence or absence of GAL (10 ng ml^−1^) and SNAP (1 μM) (*n* = 3 independent samples). **d**, Representative and quantification of labile iron in BM-mMDSCs treated with GAL and SNAP in the presence of FAC (100 μM) (*n* = 3 independent samples). **e**, Representative and quantification of C11-BODIPY 581/591 lipid peroxidation in BM-mMDSCs treated with RSL3, GAL or their combination for 4 h (*n* = 3 independent samples). **f**, Representative immunofluorescence images and quantification for Ly6C^+^4-HNE^+^ mMDSCs in size-matched CT2A and 005 GSC tumors treated with or without SNAP (30 mg kg^−1^, i.p., daily) starting on day 7 for ten doses (*n* = 3 independent samples). **g**, Principal component analysis of lipid distribution in RSL3-treated BM-MDSCs in the presence or absence of GAL for 4 h. Lipids were analyzed by ultrahigh-performance liquid chromatography–tandem mass spectrometry (*n* = 3 independent samples). **h**, Heat maps of lipid fold changes in BM-MDSCs treated with RSL3 in the presence or absence of GAL (*n* = 3 independent samples). Red indicates high values; blue indicates low values. **i**, Lipidomics profiling of BM-MDSCs shows altered PC, TG and PE PUFAs after stimulation of RSL3 in the presence or absence of GAL (*n* = 3 independent samples). Red indicates high values; blue indicates low values. **j**, Quantification of relative migration of BM-mMDSCs following stimulation with RSL3 for 4 h in the presence or absence of GAL (*n* = 3 independent samples). Data from multiple replicates are presented as the mean ± s.e.m. Statistical analysis was conducted using a two-sided Student’s *t*-test (**f**) or one-way ANOVA (**c**–**e**,**j**). Scale bars, 50 μm (**f**), 200 μm (**j**). Source data

**Extended Data Fig. 7:**
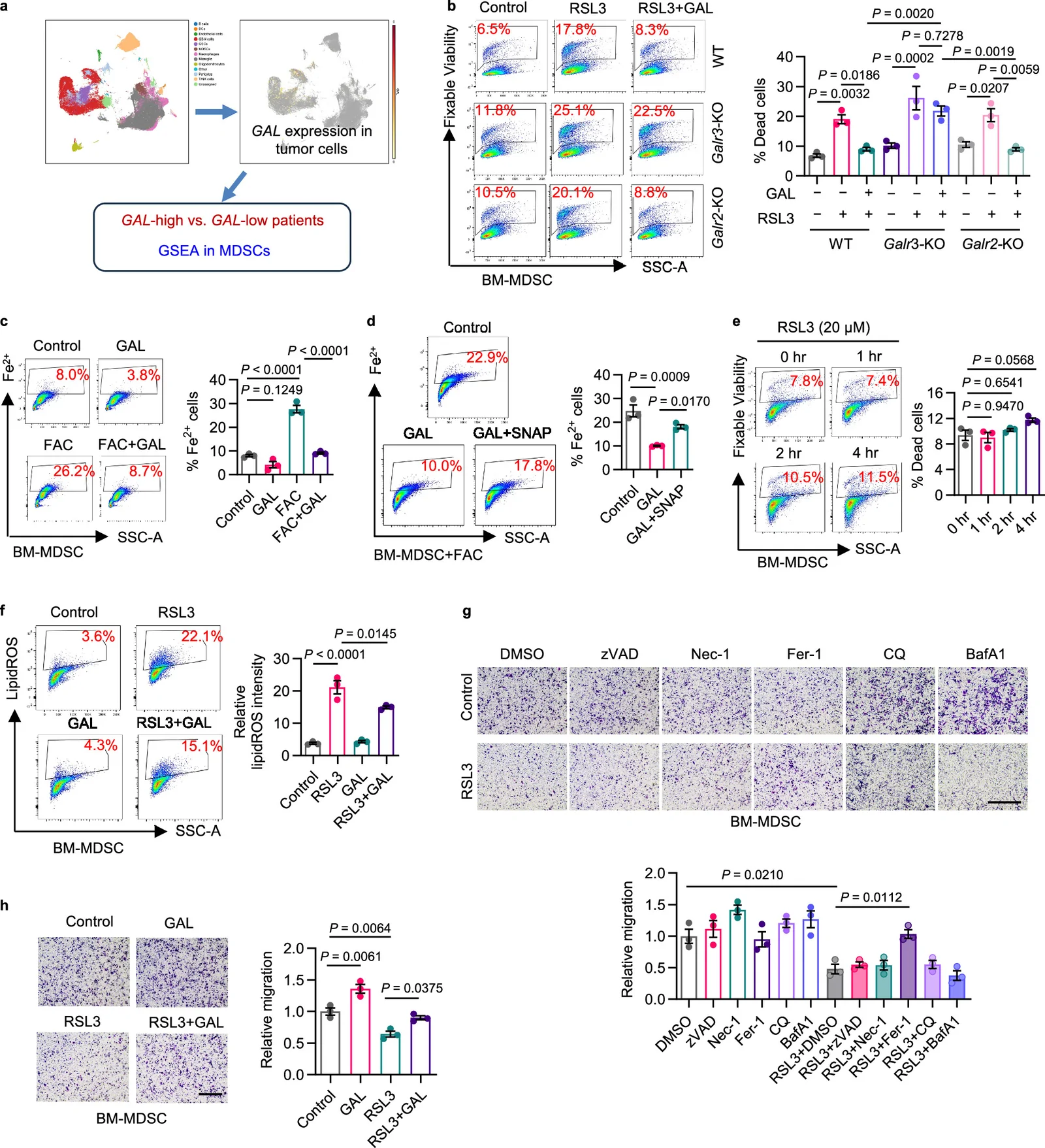
GAL protects MDSCs from ferroptosis. **a**, The strategy involved analyzing scRNA-seq data from patient GBM tumors by stratifying them into *GAL*-high and *GAL*-low groups (based on *GAL* expression in tumor cells, n = 8/group), followed by GSEA of differentially expressed genes within MDSC clusters between the two groups. **b**, Representative and quantification of the viability of BM-MDSCs (isolated from WT, *Galr3*-KO, and *Galr2*-KO mice) following stimulation with ferroptosis inducer RSL3 (20 μM) in the presence or absence of GAL (10 ng/mL). n = 3 independent samples. **c**, Representative and quantification of labile iron in BM-MDSCs following stimulation with GAL in the presence or absence of ferric ammonium citrate (FAC, 100 μM). n = 3 independent samples. **d**, Representative and quantification of labile iron in BM-MDSCs treated with GAL and SNAP (1 μM) in the FAC (100 μM). n = 3 independent samples. **e**, Representative and quantification of the viability of BM-MDSCs following stimulation inducer RSL3 at indicated concentration and timepoints. n = 3 independent samples. **f**, Representative and quantification of C11-BODIPY 581/591 lipid peroxidation in BM-MDSCs treated with RSL3, GAL, or their combination for 4 hrs. n = 3 independent samples. **g**, Representative and quantification of relative migration of BM-MDSCs following the stimulation of RSL3 for 4 hrs in the presence or absence of zVAD (10 μM), necrostatin-1 (Nec-1, 1 μM), ferrostatin-1 (Fer-1, 1 μM), chloroquine (CQ, 20 μM), and bafilomycin A1 (BafA1, 5 nM). Scale bar, 200 μm. n = 3 independent samples. **h**, Quantification of relative migration of BM-MDSCs following stimulation with RSL3 for 4 hrs in the presence or absence of GAL. Scale bar, 200 μm. n = 3 independent samples. Data from multiple replicates are presented as mean ± s.e.m. One-way ANOVA test (**b-h**). Source data

Given the crucial role of estrogen signaling in ferroptosis and cell death regulation^20,33^, we hypothesized that the GAL–GALR3–ERα axis may influence MDSC ferroptosis. We performed scRNA-seq analysis on tumors from individuals with GBM containing tumor cells and MDSCs^27^. Individuals were stratified into *GAL*-high and *GAL*-low groups on the basis of *GAL* expression in GBM cells and GSEA was then performed on differentially expressed genes within MDSC clusters (Fig. 5a and Extended Data Fig. 7a). We found that signatures related to ferroptosis, reactive oxygen species and cell death were impaired in MDSCs from the *GAL*-high group compared to the *GAL*-low group, with ferroptosis emerging as the most prominent signature (Fig. 5b). To further confirm the connection between the GAL–GALR3 axis and MDSC ferroptosis, we assessed the cell viability of GAL-treated MDSCs in the presence or absence of ferroptosis inducer RSL3. The treatment with GAL impaired RSL3-induced ferroptosis in BM-MDSCs, especially BM-mMDSCs, which was rescued by pharmacologic inhibition of GALR3 (Fig. 5c) or genetic depletion of GALR3 but not GALR2 (Extended Data Fig. 7b). Next, we assessed other ferroptosis hallmarks (for example, intracellular iron loading and lipid peroxidation) in BM-MDSCs and BM-mMDSCs, showing that GAL significantly inhibited ferric ammonium citrate (FAC)-induced MDSC intracellular iron loading, an effect that was abolished by GALR3 inhibition (Fig. 5d and Extended Data Fig. 7c,d). To evaluate lipid peroxidation, we subjected MDSCs to short-term RSL3 exposure to activate early ferroptosis signaling but without causing cell death (Extended Data Fig. 7e). C11-BODIPY assays demonstrated that GAL inhibited RSL3-induced lipid peroxidation in BM-MDSCs and BM-mMDSCs (Fig. 5e and Extended Data Fig. 7f). To confirm the role of the GAL–GALR3 axis in regulating mMDSC ferroptosis in vivo, we performed immunofluorescence analyses and found that SNAP treatment significantly increased the expression of ferroptosis marker 4-hydroxynonenal (4-HNE)^34^ in mMDSCs of CT2A and 005 GSC tumors (Fig. 5f).

Considering the critical role of lipid remodeling in ferroptosis, we conducted lipidome profiling of control and RSL3-treated MDSCs in the presence or absence of GAL. GAL treatment rescued RSL3-induced ferroptosis, as evidenced by the altered lipid composition, particularly phosphatidylcholine (PC), phosphatidylethanolamine (PE), sphingolipid and triglyceride (TG) but not lysoPE in MDSCs (Fig. 5g,h). More specifically, GAL negated RSL3-induced PC, TG and PE polyunsaturated fatty acids (PUFAs) (Fig. 5i), which are indicative of ferroptosis^35,36^, thereby suggesting a role of GAL in conferring ferroptosis resistance. To investigate the impact of ferroptosis on MDSC migration, we conducted a migration assay for RSL3-treated BM-MDSCs in the presence of various cell death inhibitors. Ferroptosis inhibitor ferrostatin 1 (Fer-1), apoptosis inhibitor Z-VAD-FMK (zVAD), necroptosis inhibitor necrostatin 1 (Nec-1) and autophagy inhibitors chloroquine (CQ) and bafilomycin A1 (BafA1) were used at low concentrations that were not sufficient to directly affect MDSC migration (Extended Data Fig. 7g). However, we found that RSL3-induced impairment of MDSC migration was rescued by Fer-1 but not by zVAD, Nec-1, CQ or BafA1 (Extended Data Fig. 7g). Accordingly, GAL treatment negated RSL3-induced downregulation of mMDSC migration (Fig. 5j and Extended Data Fig. 7h). Together, these findings indicate that GAL can endow ferroptosis resistance in mMDSCs.

### Membrane-bound *O*-acyltransferase domain-containing 1 regulates GAL–ERα axis-induced mMDSC ferroptosis resistance and migration

**Fig. 6:**
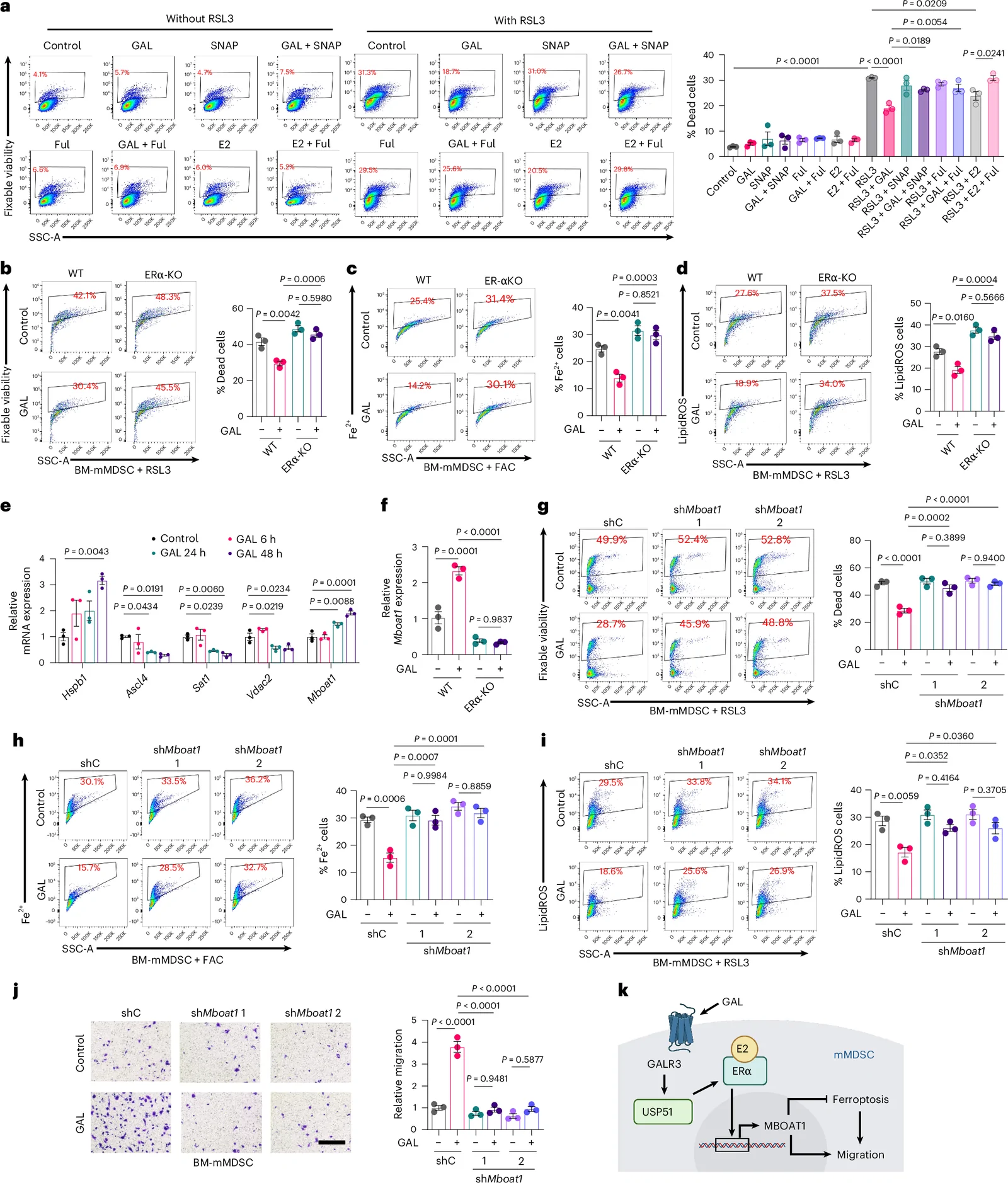
GAL endows MDSC ferroptosis resistance and migration through the ERα–MBOAT1 axis. **a**, Representative and quantification of the viability of BM-MDSC treatment with RSL3 (20 μM) for 16 h in the presence or absence of GAL (10 ng ml^−1^), SNAP (1 μM), estradiol (E2, 10 μM) and Ful (10 μM) (*n* = 3 independent samples). **b**, Representative and quantification of the viability of BM-mMDSCs (isolated from WT or ERα-KO mice) treated with RSL3 (20 μM) for 16 h in the presence or absence of GAL (10 ng ml^−1^) (*n* = 3 independent samples). **c**, Representative and quantification of labile iron in BM-mMDSCs (isolated from WT or ERα-KO mice) treated with GAL in the presence of FAC (100 μM) (*n* = 3 independent samples). **d**, Representative and quantification of C11-BODIPY 581/591 lipid peroxidation in BM-mMDSCs (isolated from WT or ERα-KO mice) treated with GAL and RSL3 for 4 h (*n* = 3 independent samples). **e**, mRNA levels of *Hspb1*, *Mboat1*, *Ascl4*, *Sat1* and *Vdac2* in BM-MDSCs treated with GAL at indicated time points (*n* = 3 independent samples). **f**, mRNA levels of *Mboat1* in BM-mMDSCs isolated from WT and ERα-KO mice and treated with GAL for 48 h (*n* = 3 independent samples). **g**, Representative and quantification of the viability of BM-mMDSCs expressing shC and sh*Mboat1* treated with RSL3 (20 μM) for 16 h in the presence or absence of GAL (10 ng ml^−1^) (*n* = 3 independent samples). **h**, Representative and quantification of labile iron in BM-mMDSCs expressing shC and sh*Mboat1* treated with GAL in the presence of FAC (100 μM) (*n* = 3 independent samples). **i**, Representative and quantification of C11-BODIPY 581/591 lipid peroxidation in BM-mMDSCs expressing shC and sh*Mboat1* treated with GAL and RSL3 for 4 h (*n* = 3 independent samples). **j**, Representative and quantification of relative migration of shC and sh*Mboat1* BM-mMDSCs following stimulation with GAL (*n* = 3 independent samples). **k**, The proposed model for GAL endowing mMDSC ferroptosis resistance and migration. GAL upregulates USP51 expression in mMDSCs by activating GALR3, increasing the stability of ERα and downstream MBOAT1 to prevent MDSC ferroptosis and promote migration. Data from multiple replicates are presented as the mean ± s.e.m. Statistical analysis was conducted using a one-way ANOVA (**a**–**j**). Scale bar, 200 μm (**j**). Source data

**Extended Data Fig. 8:**
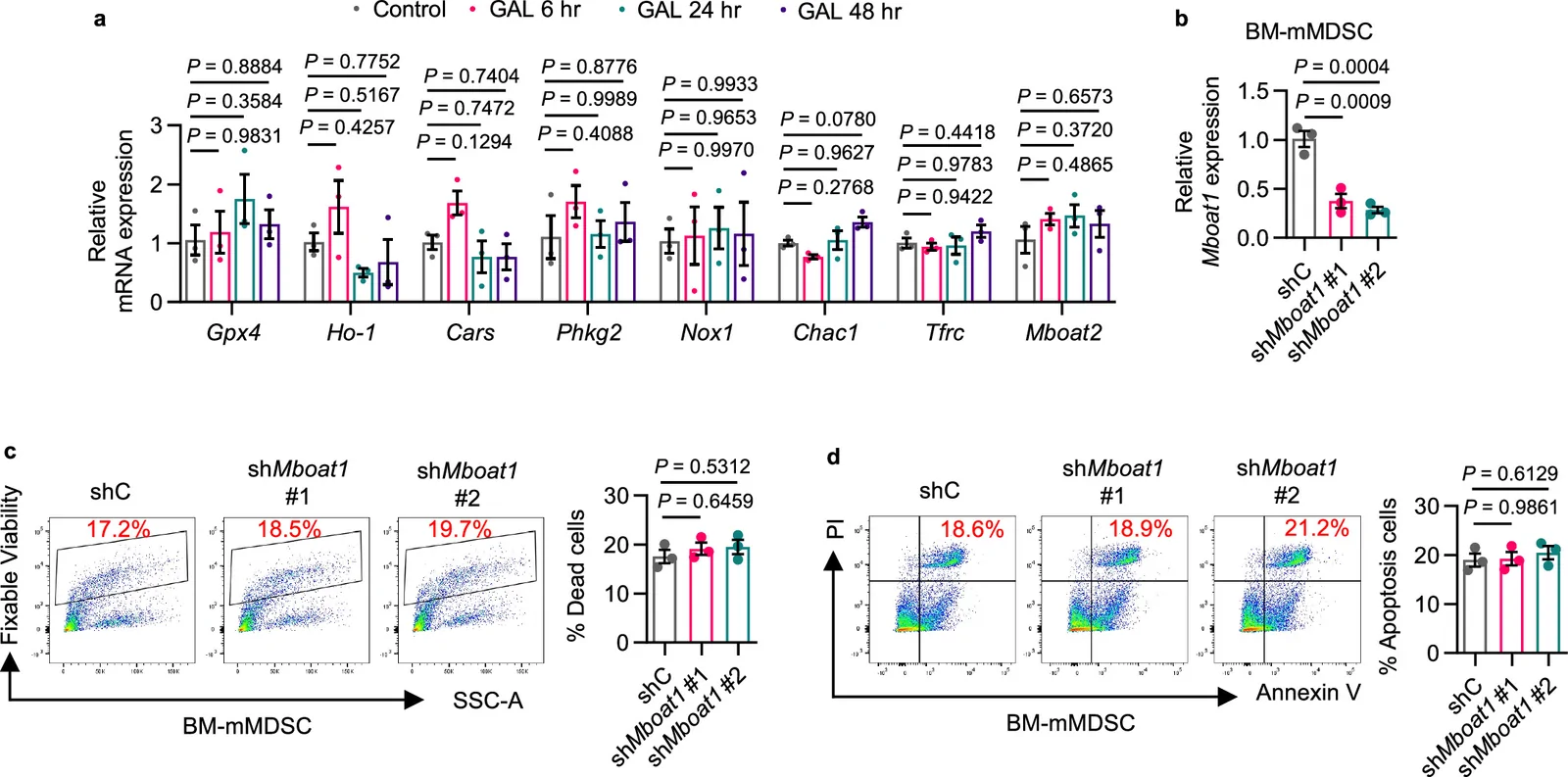
MBOAT1 does not affect some ferroptosis-related genes and cell death in mMDSCs. **a**, mRNA levels of *Gpx4*, *Ho-1*, *Cars*, *Phkg2*, *Nox1*, *Chac1*, *Tfrc*, and *Mboat2* in BM-MDSCs treated with GAL at indicated timepoints. n = 3 independent samples. **b**, mRNA levels of *Mboat1* in BM-mMDSCs expressing shRNA control (shC) and *Mboat1* shRNA (sh*Mboat1*). n = 3 independent samples. **c**, Representative and quantification of the viability of BM-mMDSCs expressing shC and sh*Mboat1*. n = 3 independent samples. **d**, Representative and quantification of the apoptosis of BM-mMDSCs expressing shC and sh*Mboat1*. n = 3 independent samples. Data from multiple replicates are presented as mean ± s.e.m. One-way ANOVA test (**a-d**). Source data

Considering the role of ERα in regulating GAL–GALR3-driven mMDSC migration, we next investigated whether ERα involves in GAL-induced ferroptosis resistance. Indeed, we found that GAL-mediated inhibition of ferroptosis was abolished by ERα inhibition, either through treatment with the ERα inhibitor Ful or through genetic deletion of ERα, indicating that ERα is required for GAL-induced ferroptosis resistance of mMDSCs (Fig. 6a–d). To further explore the downstream of ERα mediating GAL-induced ferroptosis resistance, we assessed the expression of well-established ferroptosis-related genes^22,37–39^ in control and GAL-treated BM-MDSCs. While several ferroptosis regulators (for example, *Gpx4*, *Ho-1*, *Cars*, *Phkg2*, *Nox1*, *Chac1*, *Mboat2* and *Tfrc*) were not affected (Extended Data Fig. 8a), prolonged GAL treatment (48 h) increased *Hspb1*, a suppressor of iron uptake (Fig. 6e). Conversely, genes (for example, *Acsl4*, *Sat* and *Vdac2*) involved in lipid remodeling and peroxidation during ferroptosis^38^ were significantly downregulated by GAL treatment (Fig. 6e). Given the role of membrane-bound *O*-acyltransferase domain-containing 1 (MBOAT1), a phospholipid-modifying enzyme, in regulating ER signaling-mediated ferroptosis and phospholipid remodeling^20^, we investigated whether it is required for GAL-endowed ferroptosis resistance. The results showed that GAL treatment upregulated *Mboat1* expression in BM-MDSCs (Fig. 6e) and BM-mMDSCs, an effect that was negated by ERα depletion (Fig. 6f), suggesting that MBOAT1 is a downstream of ERα in mMDSCs. Functionally, GAL-induced ferroptosis resistance and migration was abolished by the genetic knockdown of *Mboat1* in BM-mMDSCs (Fig. 6g–j and Extended Data Fig. 8b). However, *Mboat1* depletion showed no effect on cell death in BM-mMDSCs (Extended Data Fig. 8c,d). Together, these findings indicate that GAL endows mMDSC ferroptosis resistance and migration through the ERα–MBOAT1 axis (Fig. 6k).

### Combination of GALR3 antagonist and RSL3 sensitizes GBM to anti-PD1 therapy

**Fig. 7:**
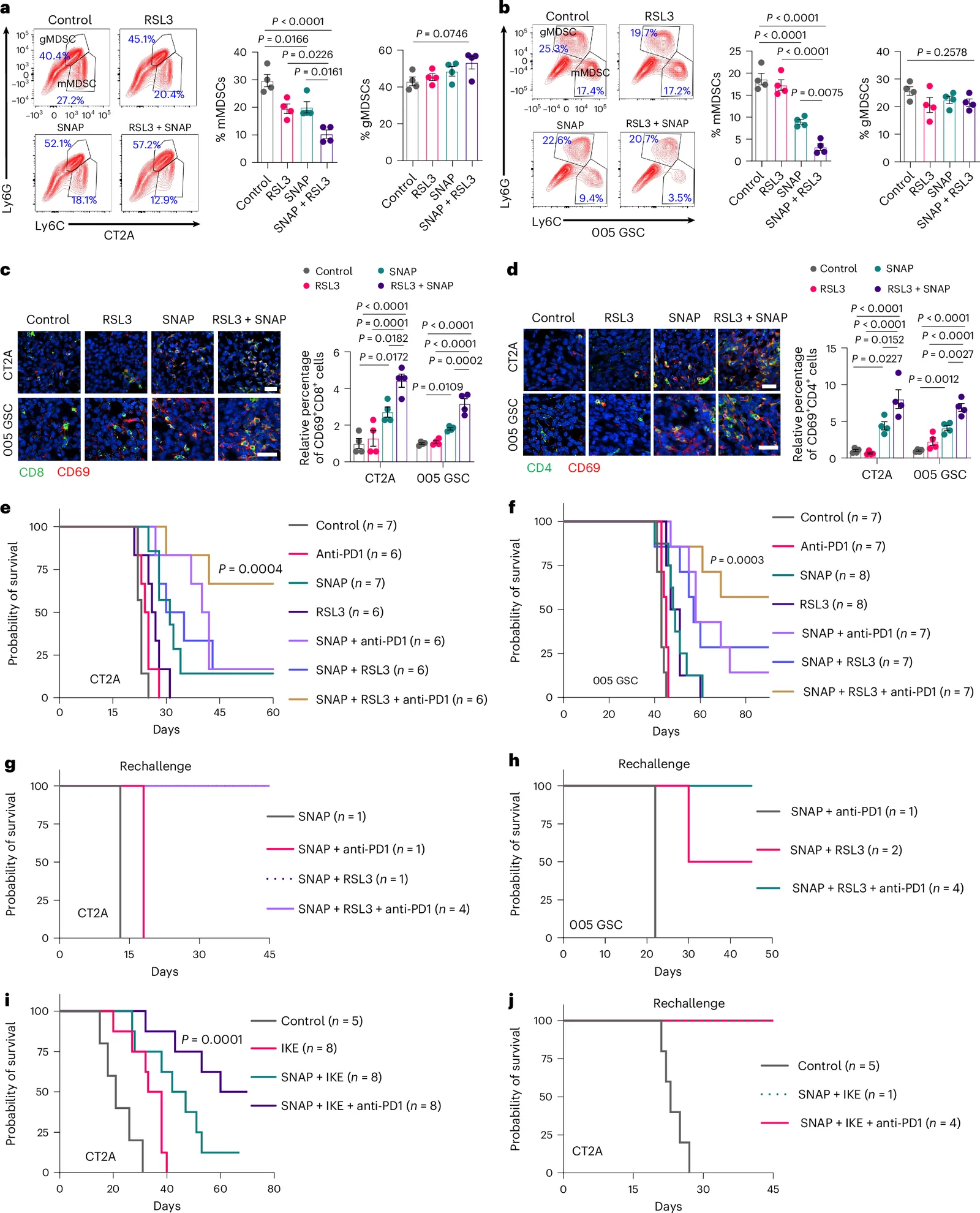
Combined SNAP and RSL3 treatment reduces mMDSCs, activates T cells and synergizes with anti-PD1 therapy in GBM. **a**, Representative images and quantification of flow cytometry for the percentage of CD11b^+^CD68^−^Ly6C^high^Ly6G^low^ mMDSCs and CD11b^+^CD68^−^Ly6C^low^Ly6G^high^ gMDSC in CT2A tumor-bearing C57BL/6 mice treated with SNAP (30 mg kg^−1^, i.p., daily, starting on day 7 for ten doses), RSL3 (50 mg kg^−1^, i.p., every other day, starting on day 7 for five doses) or their combination (*n* = 4 independent samples). **b**, Representative images and quantification of flow cytometry for the percentage of CD11b^+^CD68^−^Ly6C^high^Ly6G^low^ mMDSCs and CD11b^+^CD68^−^Ly6C^low^Ly6G^high^ gMDSCs in 005 GSC tumor-bearing C57BL/6 mice treated with SNAP, RSL3 or their combination (*n* = 4 independent samples). **c**,**d**, Coimmunofluorescence and quantification of relative CD8^+^CD69^+^ T cells (**c**) and CD4^+^CD69^+^ T cells (**d**) in CT2A and 005 GSC tumors from C57BL/6 mice treated with SNAP, RSL3 or their combination (*n* = 4 independent samples). **e**,**f**, Survival curves of C57BL/6 mice implanted with 2 × 10^4^ CT2A (**e**) or 2 × 10^5^ 005 GSC (**f**) cells. Mice were treated with anti-PD1 (10 mg kg^−1^, i.p., on days 11, 14 and 17), SNAP, RSL3 or their combinations. **g**,**h**, Survival curves of cured C57BL/6 mice that were rechallenged on day 60 with 2 × 10^4^ CT2A (**g**) and on day 90 with 2 × 10^5^ 005 GSCs (**h**). **i**, Survival curves of C57BL/6 mice implanted with 2 × 10^4^ CT2A cells. Mice were treated with anti-PD1, SNAP, IKE (40 mg kg^−1^, i.p., once daily for 2 weeks) or their combinations. **j**, Survival curves of cured C57BL/6 mice that were rechallenged on day 70 with 2 × 10^4^ CT2A cells. Control mice received either vehicle control reagents or InVivoMAb rat IgG2a isotype control (BioXCell, BE0089), as appropriate for each treatment. Data from multiple replicates are presented as the mean ± s.e.m. Statistical analysis was conducted using a one-way ANOVA (**a**–**d**) or log-rank test (**e**,**f**,**i**). Scale bars, 25 μm (**c**,**d**). Source data

**Extended Data Fig. 9:**
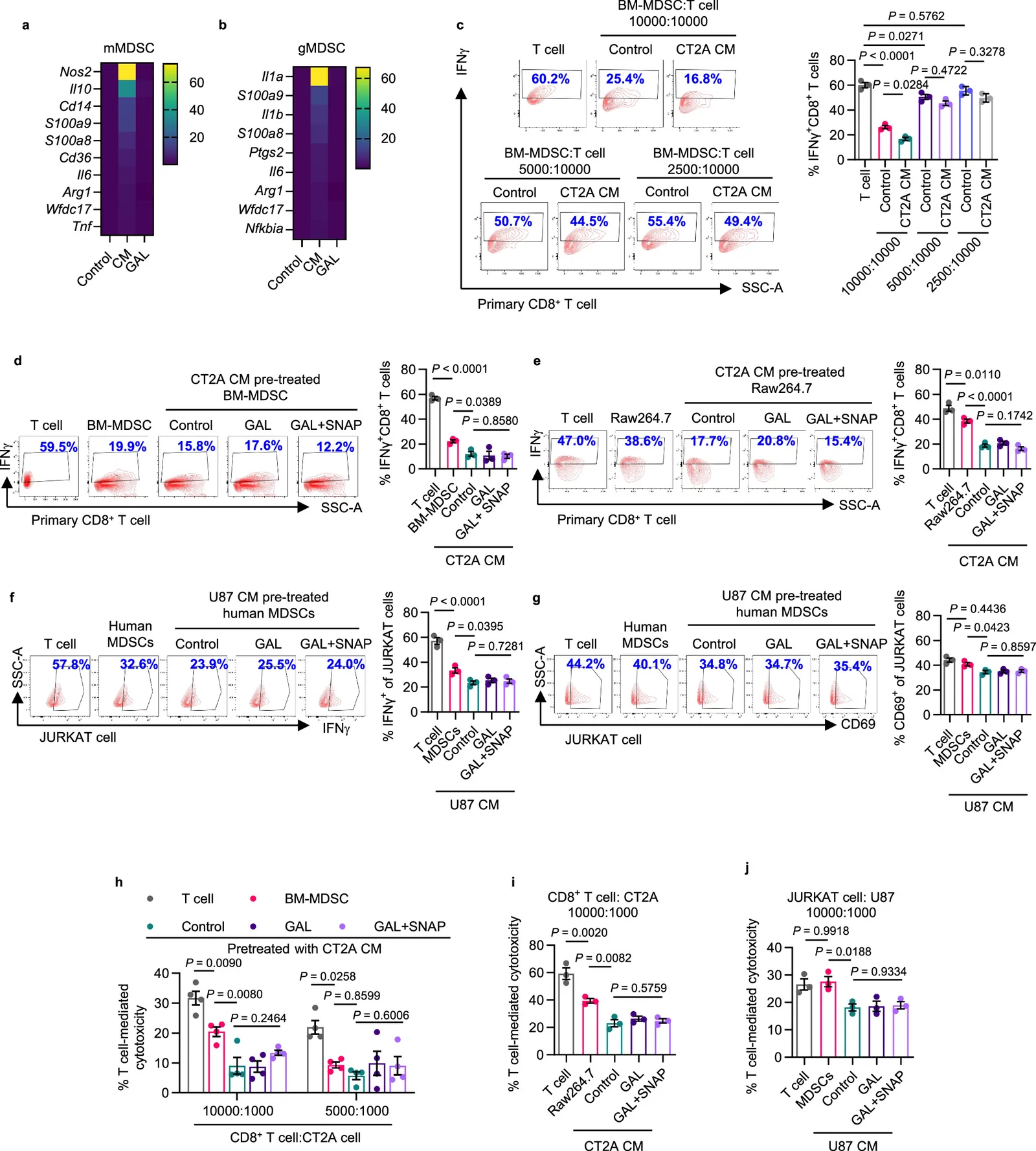
MDSC-induced T cell suppression is independent of GAL-GALR3 axis. **a,b**, Heatmap shows the relative expression levels of indicated genes in mMDSCs (**a**) and gMDSCs (**b**) incubated with the conditioned media (CM) from CT2A cells or GAL protein (10 ng/mL). n = 3 independent samples. **c**, IFNγ expression in mouse primary CD8^+^ T cells after coculturing with BM-MDSCs treated with or without CT2A CM at indicated cell-cell ratios. n = 3 independent samples. **d,e**, IFNγ expression in mouse primary CD8^+^ T cells after coculturing with BM-MDSCs (**d**) or Raw264.7-derived MDSCs (**e**) at indicated treatment conditions. n = 3 independent samples. **f**, IFNγ expression in JURKAT cells after coculturing with human MDSCs at indicated treatment conditions. n = 3 independent samples. **g**, CD69 expression in JURKAT cells after coculturing with human MDSCs at indicated treatment conditions. n = 3 independent samples. **h,i**, CT2A cancer cell cytotoxicity induced by activated mouse primary CD8^+^ T cells cocultured with GAL-incubated BM-MDSCs (**h**, n = 4 independent samples) or Raw264.7-derived MDSCs (**i**, n = 3 independent samples) in the presence or absence of SNAP (1 μM). The ratios between CD8^+^ T cells and CT2A cells are indicated. **j**, U87 cancer cell cytotoxicity induced by activated JURKAT cells cocultured with GAL-incubated human MDSCs in the presence or absence of SNAP (1 μM). The ratios between JURKAT cells and U87 cells are indicated. n = 3 independent samples. Data from multiple replicates are presented as mean ± s.e.m. One-way ANOVA test (**c-j**). Source data

Given the role of GAL in endowing MDSC ferroptosis resistance, we evaluated whether GALR3 inhibition could synergize with ferroptosis inducer for preventing MDSC infiltration into the GBM TME. As anticipated, the combination of SNAP and RSL3 exhibited a synergistic effect in blocking the infiltration of mMDSCs but not gMDSCs into CT2A and 005 GSC tumors (Fig. 7a,b). To evaluate the impact of GAL in regulating MDSC immunosuppression, we performed RNA-seq on BM-MDSCs treated with CT2A CM or GAL. We found that CT2A CM but not GAL increased the expression of immunosuppression-related genes (Extended Data Fig. 9a,b). In coculture experiments with MDSCs and T cells, we observed that CT2A CM-treated mouse BM-MDSCs and Raw264.7-derived MDSCs showed a stronger inhibitory effect on IFNγ expression in CD8 T cells at a 1:1 MDSC:T cell ratio (Extended Data Fig. 9c–e). This effect was not influenced by GAL or SNAP (Extended Data Fig. 9d,e). Similar results were obtained from human MDSC–T cell (1:1 ratio) cocultures showing that U87 CM-treated human MDSCs suppressed JURKAT T cell activation, which was not influenced by GAL or SNAP (Extended Data Fig. 9f,g). Functionally, tumor-associated MDSCs suppressed T cell-mediated tumor cell cytotoxicity independent of the GAL–GALR3 axis (Extended Data Fig. 9h–j). These findings suggest that tumor-associated MDSCs are immunosuppressive cells but their regulation is independent of the GAL–GALR3 axis.

**Extended Data Fig. 10:**
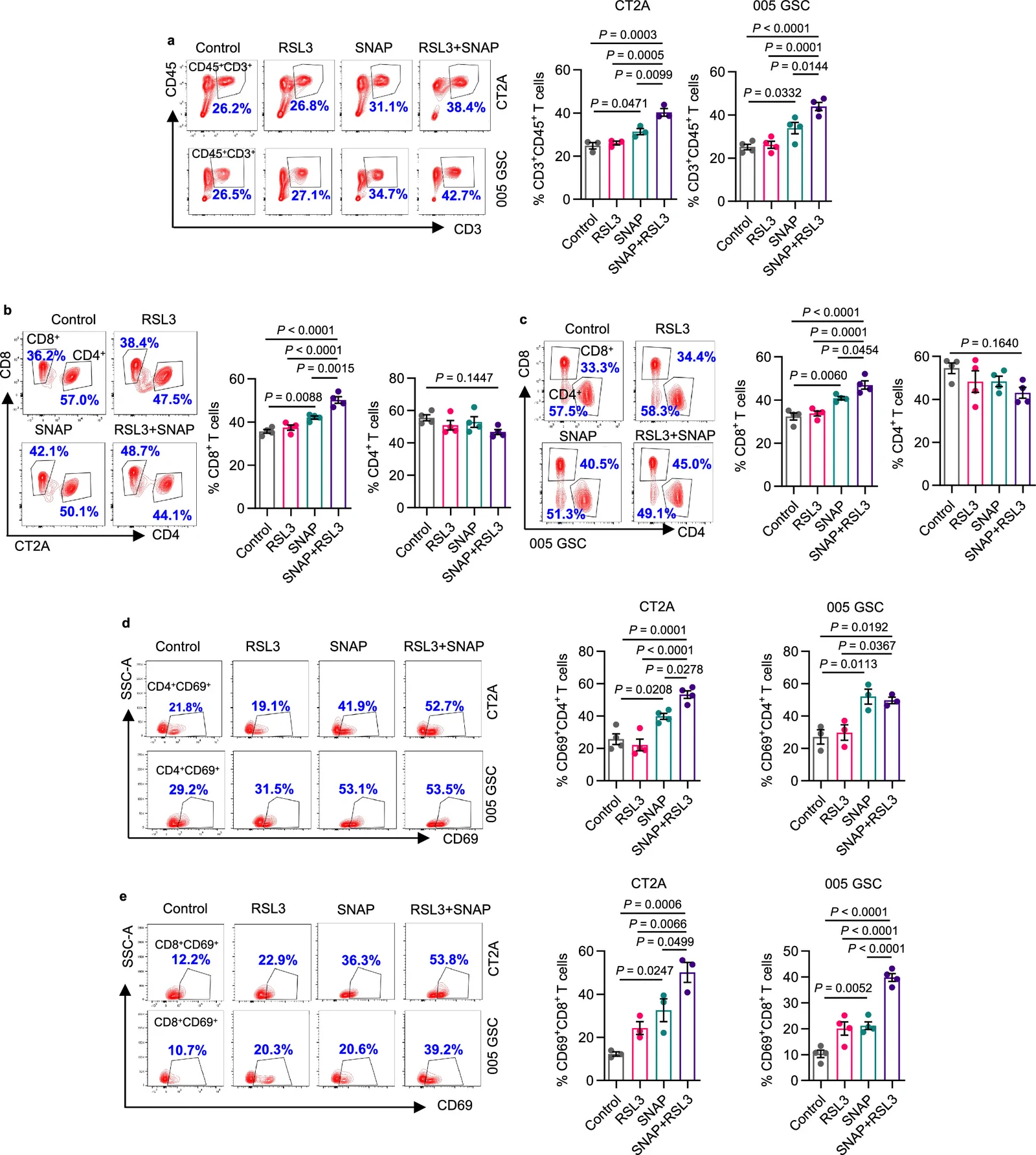
Tumor-associated MDSCs are immunosuppressive cells in GBM, and combined SNAP and RSL3 treatment activates T cells. **a**, Representative images and quantification of flow cytometry for the percentage of splenic CD45^+^CD3^+^ T cells in CT2A (n = 3 independent samples) and 005 GSC (n = 4 independent samples) tumor-bearing C57BL/6 mice. Animals were treated with SNAP (30 mg/kg, i.p., daily, starting on day 7 for 10 doses), RSL3 (50 mg/kg, i.p., every other day, starting on day 7 for 5 doses), or their combination. **b,c**, Representative images and quantification of flow cytometry for the percentage of splenic CD45^+^CD3^+^CD8^+^ and CD45^+^CD3^+^CD4^+^ T cells in CT2A (**b**) and 005 GSC (**c**) tumor-bearing C57BL/6 mice treated with SNAP, RSL3, or their combination. n = 4 independent samples. **d**, Representative images and quantification of flow cytometry for the percentage of splenic CD45^+^CD3^+^CD4^+^CD69^+^ T cells in CT2A (n = 4 independent samples) and 005 GSC (n = 3 independent samples) tumor-bearing C57BL/6 mice treated with SNAP, RSL3, or their combination. **e**, Representative images and quantification of flow cytometry for the percentage of splenic CD45^+^CD3^+^CD8^+^CD69^+^ T cells in CT2A (n = 3 independent samples) and 005 GSC (n = 4 independent samples) tumor-bearing C57BL/6 mice treated with SNAP, RSL3, or their combination. Data from multiple replicates are presented as mean ± s.e.m. One-way ANOVA test (**a-e**). Source data

Given the synergistic effect of reducing immunosuppressive mMDSCs by combining SNAP and RSL3 treatment, we hypothesized that this combination therapy strategy might affect T cell-mediated antitumor immunity. SNAP but not RSL3 increased the proportion of splenic T cells (CD45^+^CD3^+^), CD8 T cells (CD45^+^CD3^+^CD8^+^), activated CD4 T cells (CD45^+^CD3^+^CD4^+^CD69^+^) and activated CD8 T cells (CD45^+^CD3^+^CD4^+^CD69^+^), whereas CD4 T cells (CD45^+^CD3^+^CD4^+^) were unaffected. These upregulations were further heightened when SNAP was combined with RSL3 (Extended Data Fig. 10a–e). Immunofluorescence staining further confirmed the changes of activated CD8 T cells (CD69^+^CD8^+^) and activated CD4 T cells (CD69^+^CD4^+^) in CT2A and 005 GSC tumors (Fig. 7c,d). On the basis of these results, we investigated the antitumor impact of the triple therapy (SNAP + RSL3 + anti-PD1) in GBM mouse models. The triple therapy achieved a cure in 67% of CT2A-bearing mice and 57% of 005 GSC-bearing mice (Fig. 7e,f). Moreover, the combination therapy activated T cell memory, as all cured mice remained tumor free after tumor rechallenge (Fig. 7g,h). To validate the therapeutic potential of the strategy, we developed an additional triple therapy combining SNAP, anti-PD1 and imidazole ketone erastin (IKE, a ferroptosis inducer that inhibits the cystine–glutamate antiporter system Xc^−^)^20,22^ in the CT2A model. The results showed that the triple therapy cured 50% of tumor-baring mice and induced durable T cell memory (Fig. 7i,j). Together, these findings suggest that GALR3 inhibition and ferroptosis induction synergistically activate antitumor immunity, providing a therapeutic strategy to combine with anti-PD1 therapy for GBM.

## Discussion

The interplay between tumor cells and immune cells (for example, MDSCs) is essential for tumor progression and immunosuppression in GBM^15^. In this study, we investigated the molecular mechanisms for how tumor-secreted neuropeptide influences MDSC biology and contributes to the tumor–MDSC symbiosis. Specifically, we identified GAL as a key driver facilitating mMDSC infiltration into the GBM TME by activating the GALR3–USP51–ERα signaling pathway. Inconsistent with previous work in non-CNS tumors showing that ferroptosis drives immunosuppressive reprograming in neutrophils^22^, here, we discovered that GAL-triggered ERα signaling induces lipid remodeling in mMDSCs, protecting them from ferroptosis. Through this mechanism, GBM cells facilitate mMDSC infiltration into the TME, leading to resistance to immunotherapy, a phenomenon observed in individuals with GBM^40^. These findings reveal a paradigm-shifting concept that targeting MDSC infiltration and ferroptosis resistance creates a therapeutic vulnerability in GBM. The combination of GALR3 inhibition (using SNAP) and ferroptosis induction (using RSL3 or IKE) turns the TME from ‘cold’ to ‘hot’ and sensitizes GBM tumors to anti-PD1 therapy in mouse models.

MDSCs are immature myeloid cells originating from myeloid progenitors and granulocytic or monocytic precursors^41^. Most prior studies focused on how MDSCs acquire immunomodulatory functions and interact with T cells in the TME^42^. However, our understanding of how MDSCs are recruited into the TME remains unclear. Limited evidence shows that the accumulation of mMDSCs is driven by tumor-derived chemokines, such as CCL2 and macrophage migration inhibitory factor^43,44^, whereas the infiltration of gMDSCs is triggered by cancer cell-derived CXCL1/2 in mice and IL-8 in humans^45,46^, mediated through the chemokine receptor CXCR2 (ref. ^47^), which is highly expressed in both MDSCs and macrophages^48^. In this study, we revealed that neuropeptide GAL specifically triggers the infiltration of mMDSCs but does not affect gMDSCs. This selectivity is likely driven by the different expression levels of GALRs on these myeloid subpopulations, as we found that GALR3 is positively correlated with GAL and specifically highly expressed by mMDSCs in GBM tumors. Together with our recent findings showing that tumor-derived factors specifically recruit macrophages or microglia into the TME^6,25,29,49^, this study underscores the factor-dependent infiltration of mMDSCs in GBM. Given that previous reports support the differentiation of mMDSCs into macrophages in the TME^41^ and the current study indicates moderate expression of GALR3 in macrophages, it is plausible that the GAL–GALR3 axis might enhance macrophage abundance in GBM tumors. However, this hypothesis is not supported by our observations that the inhibition of GALR3 did not reduce macrophage recruitment. This phenomenon may be explained by several factors: (1) GAL is not essential for macrophage infiltration; (2) the GAL–GALR3 axis may inhibit the differentiation process; and (3) additional pathways may contribute to mMDSC-to-macrophage differentiation, acting in concert with the GAL–GALR3 axis. Further investigation is needed to test these possibilities.

GAL is widely distributed in the brain and has an important role in regulating various biological and physiological processes (for example, learning, reproduction, depression and anxiety) through the activation of GALRs^31^. Under the neuroinflammatory conditions, GAL can modulate microglia reprograming^50^. Among the GALRs, GALR3 exhibits limited expression in the healthy CNS^51^, whereas GALR1 and GALR2 have been implicated in several cancers, including colorectal and head and neck squamous cell carcinoma, where their expression correlates with poor prognosis^52,53^. Limited evidence shows that GALR3 can mediate retinal degeneration^54^; however, the role of GAL and GALR3 in cancer, especially in GBM, remains poorly understood. In this study, we revealed a role of the GAL–GALR3 axis in GBM progression by recruiting mMDSCs into the TME. Furthermore, GALR3 activation enhances USP51-mediated ERα deubiquitination, leading to sustained ERα expression in mMDSCs and facilitating their migration into the TME. This observation aligns with prior reports that estrogen signaling promotes MDSC mobilization in ovarian cancer^55^, providing insight into the molecular mechanisms linking GAL signaling and ER activity in shaping the immune landscape of GBM. In addition to the direct effect, GAL may interact with other chemokines to regulate MDSC infiltration. Indeed, previous studies have shown that GAL can influence the expression of chemokines, such as CCL2, CCL3, CCL5 and CXCL8, in macrophages^56^. Together, these findings indicate that GAL–GALR3 signaling is a critical axis for mMDSC recruitment by regulating ERα in GBM.

Estrogen signaling has a pivotal role in regulating various cell death pathways, including apoptosis, necroptosis, ferroptosis and autophagy^20,33,57^. Activation of these cell death pathways can affect MDSC metabolism, immunosuppression and survival^58,59^. In this study, we explored a link between ferroptosis and MDSC migration in GBM and found that the migratory capacity of MDSCs can be impaired by a ferroptosis inducer even after a brief exposure. Consistent with a recent study supporting a vital role of estrogen signaling in regulating ferroptosis surveillance^20^, we demonstrated that GAL-stabilized ERα protects MDSCs from ferroptosis. Mechanistically, GAL endows ferroptosis resistance through lipid remodeling in MDSCs by restraining the accumulation of PE, PC and TG PUFAs. This is in contrast to the findings observed in cancer cells, where TG PUFAs are negatively associated with the susceptibility of cancer cells to ferroptosis^35^. GBM cells sequester PUFAs into the TG pool^60^, limiting the availability of PUFAs for peroxidation during ferroptosis and thereby attenuating lipid remodeling^35^. In mMDSCs, ferroptosis simultaneously upregulates PUFA-containing PE, PC and TG, suggesting that TG does not necessarily compete with PE and PC for PUFAs. It is possible that accumulated PUFAs in the TG pool may potentiate MDSC ferroptosis, as lipolysis could be triggered to meet the high energy demands in a harsh TME. Further studies are needed to elucidate how metabolic pathways regulate the interplay between MDSC ferroptosis and mobility.

Ferroptosis in MDSCs may not always be advantageous, as a recent study offered evidence showing that it drives gMDSC immunosuppressive reprogramming in non-CNS tumors^22^. These findings may explain why ferroptosis inducers have shown limited effectiveness in tumor models^61^, which aligns with our results from in vivo GBM models showing that ferroptosis induction alone does not significantly extend the survival, excluding a major contribution from GBM cell ferroptosis. However, in the context of inhibiting GAL-induced mMDSC infiltration and ferroptosis resistance by SNAP treatment, ferroptosis inducers RSL3 and IKE impair GBM progression in mouse models. Consistent with previous reports^62,63^, our results confirm that tumor-associated MDSCs are immunosuppressive cells in GBM, supporting a combination therapy of targeting MDSC biology and T cell checkpoint blockade. Indeed, our preclinical trials demonstrated that combined SNAP and RSL3 or IKE treatment activates antitumor immunity and synergizes with anti-PD1 therapy in GBM models. Despite these promising findings, the potential off-target effects of combined SNAP and RSL3 or IKE treatment on the TME cannot be excluded. Future studies should evaluate the combined SNAP and RSL3 or IKE treatment in immunocompromised GBM mouse models to determine whether an intact immune system is required for this therapeutic strategy. Together, this study provides molecular insight into the mechanism driving MDSC infiltration and ferroptosis resistance in GBM and motivates the testing of combination regimens (SNAP + RSL3/IKE + anti-PD1) for individuals with GBM.

## Methods

### Ethical statement

We confirm that the research performed in the present study complies with all ethical regulations. All animal experiments were performed with the approval of the Institutional Animal Care and Use Committee (IACUC) at Cleveland Clinic (00003354) and Northwestern University (IS00017004 and IS00015772). Both male and female mice were included to account for sex as a biological variable but the therapeutic effects were observed to be not sex dependent.

### Cell culture

U87, U251, CT2A (gifted by R. DePinho, cell bank at MD Anderson Cancer Center) and 293 T (American Type Culture Collection, CRL-11268) cells were cultured in DMEM (Gibco, 11995-065) with 1:100 antibiotic–antimycotic (Gibco, 15140-122) and 10% FBS (Fisher Scientific, 16140071). The 005 GSCs (provided by S. D. Rabkin, Massachusetts General Hospital) were cultured in NSC proliferation medium (Millipore, SCM005) containing 20 ng ml^−1^ epidermal growth factor (PeproTech, AF-100-15) and basic fibroblast growth factor (PeproTech, 100-18B). Raw264.7 and JURKAT cells were cultured in RPMI 1640 medium, containing 10% FBS and 1:100 antibiotic–antimycotic. Raw264.7 cells were treated with 40 ng ml^−1^ mouse recombinant GM-CSF (315-03-100UG, PeproTech) and 80 ng ml^−1^ mouse recombinant IL-13 (130-094-070, Miltenyi Biotec) for 3 days before the experiment. All cells were confirmed to be free of *Mycoplasma* and were maintained at 37 °C and 5% CO_2_.

### Mice and intracranial xenograft tumor models

C57BL/6 (0000664), nude (007850) and ERα-KO (026176) mice were purchased from the Jackson Laboratory. *Galr2*-KO mice were generated through a targeted mutation in the intron of the *Galr2* gene, resulting in premature termination of translation. The original mouse line was obtained from Lexicon Genetics and provided by M. Picciotto’s lab at Yale University to the laboratory of B.K. *Galr3*-KO mice were originally obtained from the European Mouse Mutant Archive and generated by homologous recombination, targeting both coding exons of the *Galr3* gene. Both *Galr2*-KO and *Galr3*-KO lines were backcrossed to the C57BL/6N background. Mice were housed in a specific-pathogen-free animal facility under controlled environmental conditions with a 12-h light–dark cycle, an ambient temperature of 20–24 °C and a relative humidity of 30–70%. Animals had ad libitum access to standard chow and water and were monitored daily in accordance with institutional guidelines. The intracranial xenograft GBM models were generated using a previously described protocol^6^. As approved by the IACUC protocols, animals showing distress, neurological signs (for example, lethargy, hemiparesis, ataxia and seizures) or a moribund appearance were killed. At the end of the experiment, mouse brains were collected for cryosection or immune cell analysis using flow cytometry.

### Human samples

The plasma and brain tumor formalin-fixed paraffin-embedded sections of individuals with GBM (Supplementary Table 1) were obtained from the Northwestern CNS Tissue Bank. According to The George Washington University Institutional Review Board (IRB) and the guidelines from the Office of Human Research Protection, the conducted research met the criteria for exemption 4 (45 CFR 46.101(b) Categories of Exempt Human Subjects Research) and did not constitute human research.

### Isolation of mouse BM-MDSCs

Mouse BM cells were isolated using the previously described method^7^. To isolate different MDSC subpopulations, BM-MDSCs were stained with fixable viability dye (Invitrogen, 65-0865-14) and blocked using anti-CD16/CD32 cocktail (BioLegend, 101320) in 2% BSA. Cells were further stained with PE/Cy7 anti-mouse/human CD11b (BioLegend, 101216), AlexaFluor 700 anti-mouse Ly6C (BioLegend, 128024), PE anti-mouse CD68 (BD Bioscience, 566386) and FITC anti-mouse Ly6G (BioLegend, 127606) for 1 h. The following cell subsets were sorted using BD FACSAria (BD Biosciences): mMDSCs (CD11b^+^CD68^−^Ly6C^high^Ly6G^low^), gMDSCs (CD11b^+^CD68^−^Ly6C^low^Ly6G^high^), non-MDSCs (CD11b^+^CD68^−^Ly6C^−^Ly6G^−^) and macrophages (CD11b^+^CD68^+^).

### BM chimera generation

BM cells were isolated from WT and *Galr3*-KO C57BL/6 donor mice. Recipient mice (at least 6 weeks of age) were subjected to 1,100 cGy of total-body irradiation using an X-RAD320 irradiator (Precision). Following irradiation, mice were intravenously transplanted with BM cells from either WT or *Galr3*-KO donors. To prevent infections during BM reconstitution, Baytril was administered in the drinking water.

### Isolation of human MDSCs and mouse CD8^+^ T cells

Healthy human whole peripheral blood was purchased from StemCell Technologies (70508.1), which was obtained from healthy donors using IRB-approved consent forms and protocols. Samples were provided to the investigators in deidentified form and no donor-identifiable information was accessible to the research team. PBMCs were isolated using Lymphoprep (StemCell Technologies, 07801) density gradient medium and SepMate tube (StemCell Technologies, 85450). The isolated PBMCs were incubated with human IL-6 (Miltenyi Biotec, 130-093-929) and human recombinant GM-CSF (StemCell Technologies, 78140.1) for 6 days to derive MDSCs. Subsequently, the EasySep human CD33 positive selection kit II (StemCell Technologies, 17876) and EasySep magnet (StemCell Technologies, 18000) were used to enrich CD33^+^ MDSCs.

The mouse primary CD8^+^ T cells were isolated as we reported previously^6^. Briefly, CD8^+^ T cell isolation kit (Miltenyi Biotec, 130-104-075) or EasySep mouse CD8^+^ selection kit (StemCell Technologies, 18953) were used to sort CD8^+^ cells from the spleen of C57BL/6 mice. Then, 30 U per ml mouse IL-2 and Dynabeads mouse T-activator CD3/CD28 (Gibco, 11456D) were added to RPMI with 10% FBS for expansion and activation of T cells.

### Immunoblotting

Western blotting analysis was used to determine protein expression in cells as a standard protocol we described previously^6^. After running gels and transferring proteins to the blots, primary antibodies (1:1,000 dilution), including GAL (Invitrogen, PA5-95500), ERα (Invitrogen, MA1-80216), ERβ (Invitrogen, PA1-311), histone H3 (Cell Signaling Technology, 9715S), USP51 (Invitrogen, PA5-68358) and actin (Cell Signaling Technology, 4967S) were incubated with the membrane overnight at 4 °C. After washing, membrane was incubated with corresponding horseradish-peroxidase-linked secondary antibodies (for example, anti-mouse (Cell Signaling Technology, 7076) and anti-rabbit (Cell Signaling Technology, 7074S)). Membranes were developed with enhanced chemiluminescence or Femto maximum-sensitivity substrates (Thermo Scientific, 32209 and 34096). All experiments were repeated at least 2–3 times.

### Plasmids and viral transfections

To generate shRNA expression cells, shRNAs targeting mouse *Gal*, *Usp51* and human *GAL* in the pLKO.1 vector (Sigma, SHC001) were used in this study. Then, 293T cells were used to generate lentiviral particles, which were assembled by 8 μg of pLKO.1 vector and packaging plasmids, including 2 μg of pMD2.G (Addgene, 12259) and 4 μg of psPAX2 (Addgene, 12260). After 48 h of transfection, CM containing lentiviral particles was collected and added to the target cell for 48 h of incubation. Infected cells were selected by 2 μg ml^−1^ puromycin (Millipore, 540411). Immunoblots were used to determine the protein expression. The following shRNA sequences (from Sigma) were selected for further use after validation: *Gal* 3, TRCN0000240594; *Gal* 4, TRCN0000240595; *Usp51* 3, TRCN0000255143; *Usp51* 4, TRCN0000255145; *Mboat1* 1, TRCN0000262727; *Mboat1* 2, TRCN0000262729; *GAL* 1, TRCN0000083173; *GAL* 2, TRCN0000083174; *GAL* 3, TRCN0000083176.

### Migration assays

To monitor the MDSC migration in real-time, we used Incucyte live-cell analysis system and TrackMate trajectories analysis system to determine the movement speed of MDSCs^6,64^. Briefly, BM-MDSCs were cultured in a 96-well plate overnight and then transferred into the Incucyte after the treatment. The images were captured every 30 min and a LoG detector was used for segmentation. The linear assignment problem was used to link images on the basis of the respective distances between MDSCs over the time frames. Additionally, a transwell migration assay was performed on MDSCs as described previously^6,49^. Briefly, 1 × 10^4^ MDSCs were suspended in the medium with pretreatment, such as GALRs antagonists (SNAP, M40 and M871), Ful, RSL3 and different cell death pathway inhibitors. After the pretreatment, MDSCs were washed with PBS and seeded into 5.0-μm permeable polycarbonate membrane inserts (Corning, 07-200-149) with serum-free medium. The receiver wells contained GAL recombinant protein (OriGene, TP307053) in serum-free medium or GBM cell-derived CM. After 24 h, the migrated MDSCs were fixed in 10% PFA and stained with crystal violet (Sigma, C-3886). The number of migrated MDSCs was recorded using an EVOS microscope and analyzed by ImageJ (National Institutes of Health).

### Incucyte live-cell proliferating assay

Cells were seeded into 96-well plates (Corning, 3599) and cultured overnight. Cell growth was then monitored for 72 h using the IncuCyte Zoom live-cell analysis system (Sartorius). Cell proliferation rate was calculated using the following formula: (cell confluency at the indicated timepoint − cell confluency at 0 h)/cell confluency at 0 h.

### Viability assay

MDSCs were isolated from ERα-KO mice or from WT mice in which cells were either transfected with sh*Mboat1* or pretreated with GAL recombinant protein, SNAP, Ful and E2 for 1 h. RSL3 was added to the corresponding group to induce ferroptosis for 1–6 h. After treatment, MDSCs were collected and washed with PBS. Fixable viability dye (Invitrogen, 65-0865-14) was added to MDSCs for 10 min on ice. Following washing with PBS and fixing with fixation buffer (BioLegend, 420801), the signal of the ferroptotic MDSCs was read in a BD FACSymphony A5 flow cytometer. Cell viability was calculated using FlowJo version 10.10.

### Measurement of lipid peroxidation

MDSCs were seeded at appropriate density in six-well plates and subjected to RSL3 with or without the pretreatment of GAL and SNAP. After the treatment, MDSCs were collected and stained with BODIPY 581⁄591 C11 reagent (Fisher Scientific, D3861) for 30 min. The labeled MDSCs were collected and washed with PBS. A BD FACSymphony A5 flow cytometer and FlowJo version 10.10 were used to analyze fluorescence signals. The ratio of intensity in Texas red to the intensity in FITC represents the oxidation of BODIPY C11.

### Detection of labile iron(II) ions

MDSCs were seeded in six-well plates and left overnight. The MDSCs were then treated with or without SNAP and GAL, with or without supplementation of FAC (MedChemExpress, HY-B1645). After the treatment, MDSCs were collected and stained with BioTracker far-red labile Fe^2+^ live-cell dye (MilliporeSigma, SCT037) for 1 h at 37 °C. The signal of labile Fe^2+^ was detected using a BD FACSymphony A5 flow cytometer, followed by analysis using FlowJo version 10.10.

### CHX chase assay

To determine the protein stability of ERα, 50 mg ml^−1^ CHX (MilliporeSigma, 239763-M) was added to the medium at a 1:1,000 ratio for culturing MDSCs with or without GAL pretreatment. The cells were scraped at different time points and lysed to extract proteins. The ERα expression was determined by western blotting.

### Immunofluorescence

Fresh brain tumor samples were isolated from GBM mouse models and subjected to cryosectioning. The mouse or human tumor slides were blocked with 5% goat serum at room temperature for 30 min and then incubated with primary antibodies (1:100 dilution), including CD69 (Santa Cruz, sc-373799), CD11b (Abcam, ab8878), F4/80 (Cell Signaling, 30325S), ERα (Invitrogen, MA1-80216), ERβ (Invitrogen, PA1-311), 4-HNE (Invitrogen, MA5-27570), CD14 (Abcam, ab181470) or CD66b (NOVUS, NB100-77808) overnight at 4 °C. The secondary antibody cocktail was then applied to slides for 1 h at room temperature. Coverslips were mounted to the slides using DAPI and antifade mounting medium (Vector Laboratories, H-1200-10). The signals in tumor tissues were captured using a Nikon AX/AX R confocal microscope.

### RT–qPCR

RNA of MDSCs was isolated using the RNeasy Mini Kit (Qiagen, 74106). The concentration of RNA was determined by the NanoDrop spectrophotometers. cDNA was synthesized with all-in-one 5× RT master mix (Applied Biological Materials, G592) in a T100 thermal cycler (Bio-Rad). qPCR reactions were performed using a CFX Connect real-time PCR detection system (Bio-Rad) and SYBR green PCR master mix (Bio-Rad, 1725275). For quantifying mRNA expression levels, the threshold cycle values for targeted genes were normalized to the expression levels of *Actb*. RT–qPCR primers (from Sigma) are listed in Supplementary Table 2.

### Flow cytometry

To assess myeloid cell infiltration in mouse brain tumor tissues, we conducted flow cytometry analysis using a previously established protocol^6,7^. The cells were incubated with an antibody cocktail (1:100) containing PE/Cy7 anti-mouse/human CD11b, AlexaFluor 700 anti-mouse Ly6C and FITC anti-mouse Ly6G for 1 h on ice. Following this, the surface proteins-labeled cells were fixed in a fixation buffer (BioLegend, 420801) and stored overnight at 4 °C. GALR antibodies (1:100), including GALR1 (G-Biosciences, ITT1844), GALR2 (G-Biosciences, ITT1845) and GALR3 (Abcam, ab190694), were added to the antibody cocktail followed by the corresponding AlexaFluor 594-conjugated secondary antibody to evaluate GALR expression in each cell subpopulations. For staining intracellular proteins, cells were incubated in fixation buffer for 20 min at room temperature after surface protein staining as mentioned above. The permeabilization was conducted by incubating cells with perm buffer (0.1% Triton X-100 in PBS) for 5 min. Single-cell suspensions were then washed with perm buffer two times and incubated with PE anti-mouse CD68 and ERα antibody (Invitrogen, MA1-80216; 1:100), followed by corresponding AlexaFluor 594-conjugated secondary antibody. After two washes with perm buffer, myeloid cells were subsequently analyzed using either the BD FACSymphony A5 or BD LSRFortessa and the resulting data were analyzed using FlowJo.

To analyze the splenic T cells in GBM mouse models, we followed a standard protocol to isolate splenic cells^6^. T cells were labeled with the following antibodies (1:100): AlexaFluor 488 anti-mouse CD3 (BioLegend, 100210), PerCP/Cyanine5.5 anti-mouse CD45 (BioLegend, 103132), PE/Cy7 anti-mouse CD69 (BioLegend, 104512), BV711 anti-mouse CD8 (BioLegend, 100747) and BUV395 anti-mouse CD4 (BD Bioscience, 740208). The single-cell suspensions were then analyzed using BD FACSymphony A5 or BD LSRFortessa and the data were analyzed with FlowJo. Gating strategies are shown in Supplementary Fig. 1.

### ELISA

The concentration of GAL in human plasma and supernatant of GBM cell-derived cell culture medium was measured by ELISA using the human GAL ELISA kit (MyBioSource, MBS2505508) according to the manufacturer’s instructions. The Biotek Synergy 2SL microplate reader was used to read the optical density (OD) value of the ELISA plate.

### MDSC–T cell coculture

The MDSCs were preincubated with or without CM derived from CT2A or U87 cells before being cocultured with T cells. Mouse primary CD8^+^ T cells were activated using Dynabeads mouse T-activator CD3/CD28 (Gibco, 11456D). The cancer cell CM-treated MDSCs were then incubated with GAL and SNAP. After washing, the MDSCs were added to a six-well plate to coculture with the activated T cells at a ratio of 1:1, 1:2 or 1:4. At the end of the coculture period, all cells were collected and analyzed for the CD45^+^CD8^+^IFNγ^+^ CD8^+^ T cell and CD11b^−^IFNγ^+^ and CD11b^−^CD69^+^ JURKAT cell populations using the BD FACSymphony A5, following a previously described protocol^6^.

### Cytotoxicity assay

The Cytotox96 nonradioactive cytotoxicity assay kit (Promega, G1780) was used to determine the cytotoxicity of CD8^+^ T cells on GBM cells. Briefly, activated mouse primary CD8^+^ T cells or human JURKAT T cells were first cocultured with matched MDSCs with different pretreatments and CT2A or U87 cell-derived CM. T cells were then collected for further coculturing with CT2A or U87 cells (target cells). T cell-induced cytotoxicity was determined according to the manufacturer’s instructions. The OD in 96-well plates was measured using a Biotek Synergy 2SL microplate reader. Relative lactate dehydrogenase release was calculated as follows: (OD value of coculture − OD value of effector spontaneous − OD value of target spontaneous)/(OD value of target maximum − OD value of target spontaneous).

### RNA-seq

BM-MDSCs were derived from mouse primary BM cells and treated with recombinant GAL protein (10 ng ml^−1^) or CT2A cell-derived CM. RNA was then extracted from the MDSCs and sequenced using a previously established protocol^6^. Oligo-dT-based libraries were prepared and analyzed using the Novaseq 6000 system (paired-end) at The University of Chicago Functional Genomics facility (RRID: SCR_019196). RNA-seq quality control and mapping were performed on the Galaxy server^7^. Raw FASTQ data were trimmed using the Trimmomatic function and the trimmed reads were then mapped to the mouse (mm10) genome using the HISAT2 program. BAM data from two flowcells were merged using SAMtools and gene expressions were calculated using FeatureCounts. Differentially expressed genes were identified using the Limma model and GSEA was conducted on the basis of the differential expression table.

### Co-immunoprecipitation assay

Co-immunoprecipitation was performed using the Invitrogen Dynabeads protein G IP kit (Thermo Fisher Scientific, 10007D)^6,25^. Briefly, MDSCs were isolated from ERα-KO and WT mice in which cells were either transfected with sh*Usp51* or treated or without recombinant GAL protein and SNAP and then subjected to RIPA lysis buffer to extract protein. Next, 1.5 mg of magnetic beads were separated with a magnetic stand and incubated with ERα antibody or IgG isotype control (Cell Signaling Technology, 2729) for 10 min at room temperature. The MDSC lysate was then incubated with the antibody-conjugated magnetic beads overnight at 4 °C with rotation. The immunoprecipitated antibody–antigen complex was eluted from the beads and subjected to immunoblotting to detect ubiquitination by detecting the binding of ubiquitin on purified ERα from MDSC using ubiquitin antibody (Cell Signaling Technology, 43124S).

### Lipidomic analyses

To investigate the role of GAL in early ferroptosis-associated lipid remodeling, we pretreated BM-MDSCs with GAL protein (10 ng ml^−1^) in combination with a 4-h stimulation of RSL3 (20 μM) overnight before conducting a lipidomic analysis. After the treatment, MDSCs were collected and washed with PBS. The fresh cell pellets were then sent to the Metabolomics Platform at the University of Chicago for analysis. Lipid was extracted by a modified liquid–liquid extraction method^65^. Lipid species were separated by chromatography using a Thermo Scientific Accucore C30 (2.1 × 150 mm, 2.6 μm) column coupled to a Vanquish Horizon ultrahigh-performance liquid chromatography system and an IQ-X tribrid mass spectrometer. The Thermo Scientific LipidSearch software (version 5.0) was used to generate the list of identified lipids using the following parameters: precursor tolerance, ±3 ppm; production tolerance, ±5.0 ppm; product threshold, 1.0. The [M + H] adduct was used to identify and quantify hexosylceramide, sphingomyelin, sphingosine, methylPC, coenzyme Q, acylcarnitine, LysoPC, LysoPE, PC and PE species, whereas the [M + NH] adduct was for the TG, diglyceride and cholesteryl ester species. These identified lipid species were quantify using the Compound Discoverer 3.3 and Skyline software.

### Bioinformatic analysis of human GBM datasets

The 63 neuropeptide genes encoding secreted proteins were downloaded from the HGNC database. TCGA GBM dataset or other available datasets from GlioVis and cBioPortal were used to conduct gene correlation analyses, signature score calculations, GSEA (hallmark pathways) and survival analyses, as we previously described^7,25^. The single-cell sequencing data of tumors from individuals with GBM were downloaded and analyzed by using the BBrowser BioTuring under Talk2Data platform. Data from public repositories were integrated and used in this study, including GSE182109 (ref. ^27^) and GSE135045 (ref. ^28^). The single-cell gene expression pattern of GAL in GBM/GSC cells was analyzed. On the basis of GAL expressions in tumor cells, individuals were regrouped into *GAL*-low and *GAL*-high subgroups. MDSCs from each group were selected for GSEA analysis. The brain TIME dataset^66^, generated by RNA-seq of isolated CD45^−^ tumor cells, macrophages, microglia, CD4 and CD8 T cells from tumors of individuals with GBM, was used to assess *GAL* expression. The 38-gene MDSC signature (Supplementary Table 3)^30^ was used for analyzing MDSC content in GBM TCGA tumors and clustering them into MDSC-high and MDSC-low subgroups.

### Statistical analysis

No statistical methods were used to predetermine sample sizes but our sample sizes are similar to those reported in previous publications^5–8,23,64,67^. Data collection and analysis were not performed blind to the conditions of the experiments. Randomization was not relevant for certain mechanistic in vitro experiments because all treatment conditions were applied to genetically and biologically comparable cell populations derived from the same source. For in vivo experiments, randomization was not performed because animals were genetically identical and housed under identical conditions; as such, no baseline differences were expected between groups. No data were excluded from analysis. Statistical analysis for comparisons between groups was performed using GraphPad Prism 10 (GraphPad Software). All the measurement data were presented as the means ± s.e.m. and assumed to be *t*-distributed. Correlation analysis was conducted using the Pearson test to determine the Pearson *R* correlation coefficient and associated *P* value. The survival analysis for animal models was determined by conducting a log-rank (Mantel–Cox) test. Comparisons between two groups were conducted using Student’s *t*-tests and comparisons among multiple groups were evaluated using a one-way analysis of variance (ANOVA) with Tukey’s post hoc test. All experiments shown in the figures represent biological replicates and each sample was analyzed with three technical replicates. The numbers of independent samples were noted in the figure legends. Data distribution was assumed to be normal but this was not formally tested.

### Reporting summary

Further information on research design is available in the Nature Portfolio Reporting Summary linked to this article.

## Supplementary information

Supplementary Information Supplementary Fig. 1.

Reporting Summary

Supplementary Table

## Source data

Source Data Fig. 1 Statistical source data.

Source Data Fig. 1 Unprocessed western blots.

Source Data Fig. 2 Statistical source data.

Source Data Fig. 3 Statistical source data.

Source Data Fig. 4 Statistical source data.

Source Data Fig. 4 Unprocessed western blots.

Source Data Fig. 5 Statistical source data.

Source Data Fig. 6 Statistical source data.

Source Data Fig. 7 Statistical source data.

Source Data Extended Data Fig. 1 Statistical source data.

Source Data Extended Data Fig. 2 Statistical source data.

Source Data Extended Data Fig. 3 Statistical source data.

Source Data Extended Data Fig. 4 Statistical source data.

Source Data Extended Data Fig. 5 Statistical source data.

Source Data Extended Data Fig. 5 Unprocessed western blots.

Source Data Extended Data Fig. 6 Statistical source data.

Source Data Extended Data Fig. 6 Unprocessed western blots.

Source Data Extended Data Fig. 7 Statistical source data.

Source Data Extended Data Fig. 8 Statistical source data.

Source Data Extended Data Fig. 9 Statistical source data.

Source Data Extended Data Fig. 10 Statistical source data.

## Data Availability

The sequencing datasets generated in this study were deposited to the Gene Expression Omnibus (GEO) under accession number GSE284502. The lipidomics data are available through the Metabolomics Workbench under accession number ST004928. The neuropeptide genes encoding secreted proteins are available from the HGNC database (https://www.genenames.org/data/genegroup/#!/group/1902). TCGA GBM data are available through GlioVis (http://gliovis.bioinfo.cnio.es) and cBioPortal (https://www.cbioportal.org). Previously published single-cell sequencing data are available from the GEO, including GSE182109 and GSE GSE135045. The brain TIME dataset used in this study is publicly available online (https://joycelab.shinyapps.io/braintime). Additional data supporting the findings of this study are available within the article and its Supplementary Information or upon request from the corresponding author. Source data are provided with this paper.
