# Supplementary material for: Galanin impairs tumor immunity in glioblastoma by promoting infiltration and ferroptosis resistance of myeloid-derived suppressor cells
Source: Nat Cancer. 2026 Aug 25;7(9):1387–405. doi: 10.1038/s43018-026-01221-3 (PMC13600933; doi:10.1038/s43018-026-01221-3)
Supplement: Supplementary file 2 [file 43018_2026_1221_MOESM1_ESM.pdf]

nature cancer

Supplementary information

https://doi.org/10.1038/s43018-026-01221-3

Galanin impairs tumor immunity in glioblastoma by promoting infiltration and ferroptosis resistance of myeloid-derived suppressor cells

In the format provided by the authors and unedited

Supplementary Fig. 1.

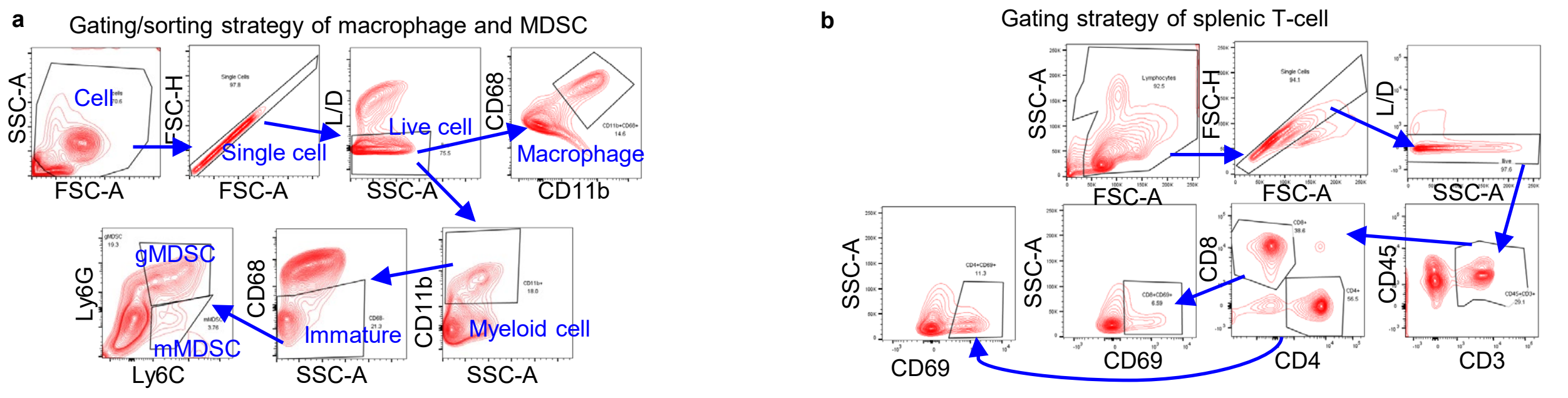

Supplementary Fig. 1. Gating strategies for flow cytometry analyses. a, Contour plots demonstrating the gating/sorting strategy of mouse macrophage, mMDSCs and gMDSCs. The gating panel corresponds to Figs. 2b, 2e, 3a, 3j-l, 4f, 4g, 4i, 4j, 7a, and 7b, and Extended Data Figs. 1r, 3a, 3j-l, 5j-m, 5o-s, 5u, and 5v. **b**, Contour plots demonstrating the gating strategy of splenic T-cells from tumor-bearing mice. The gating panel corresponds to Extended Data Figs. 2b-g, 3c, 4a-h, and 10a-e.
