# Supplementary material for: Galanin impairs tumor immunity in glioblastoma by promoting infiltration and ferroptosis resistance of myeloid-derived suppressor cells
Source: Nat Cancer. 2026 Aug 25;7(9):1387–405. doi: 10.1038/s43018-026-01221-3 (PMC13600933; doi:10.1038/s43018-026-01221-3)

Extended Data Fig. 6a

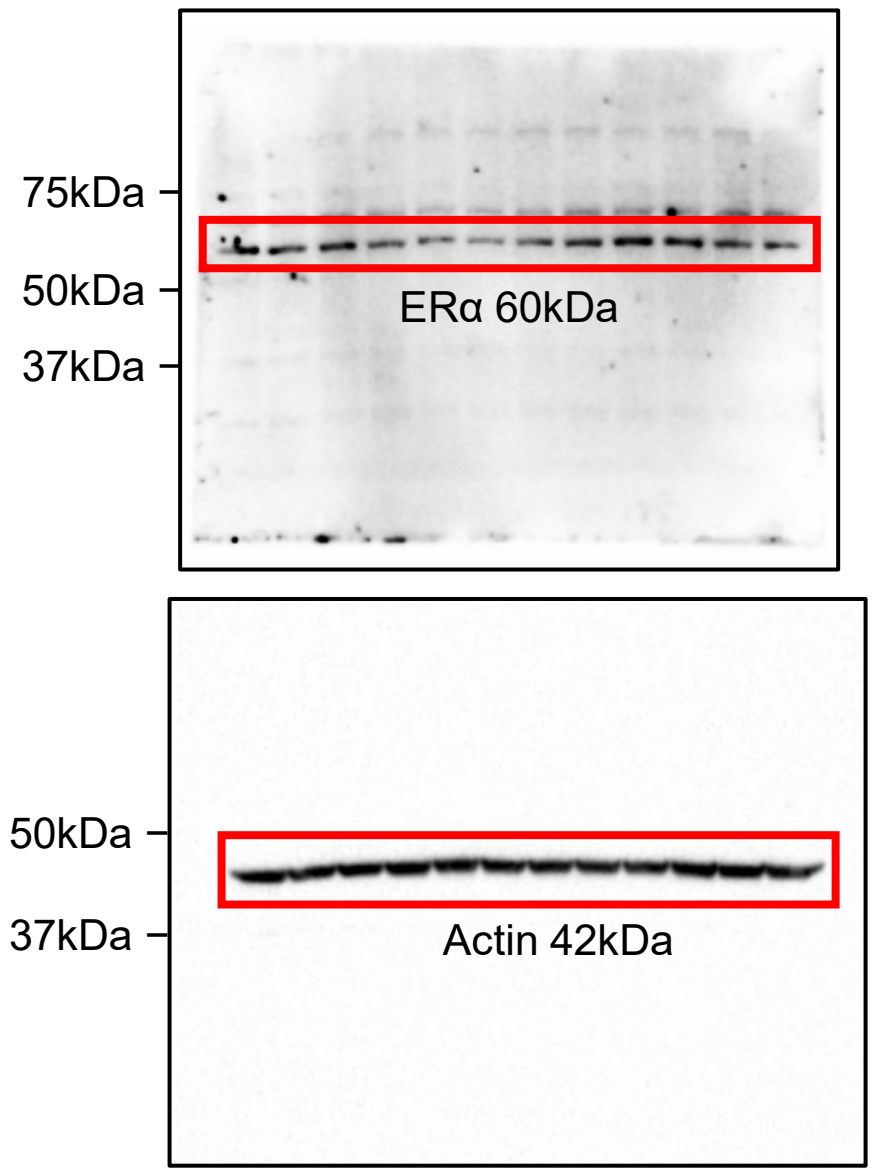

Extended Data Fig. 6b

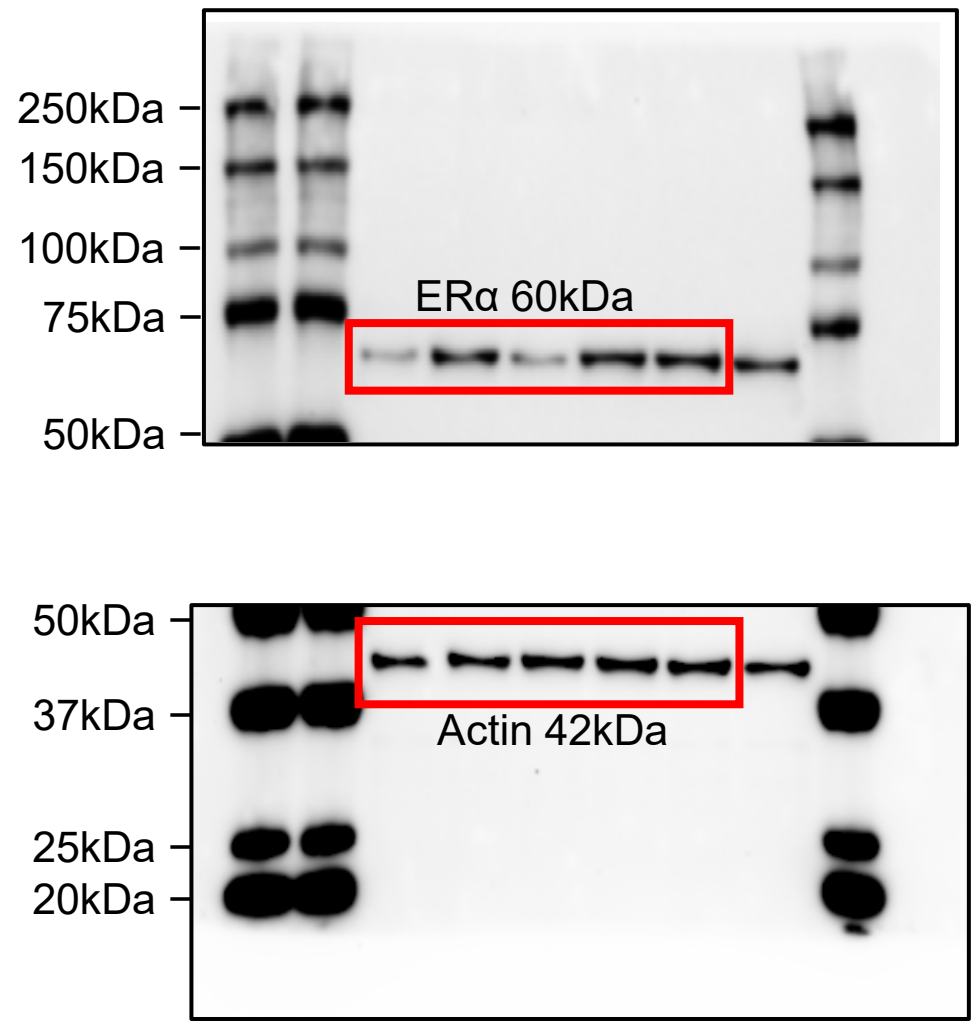

Extended Data Fig. 6c

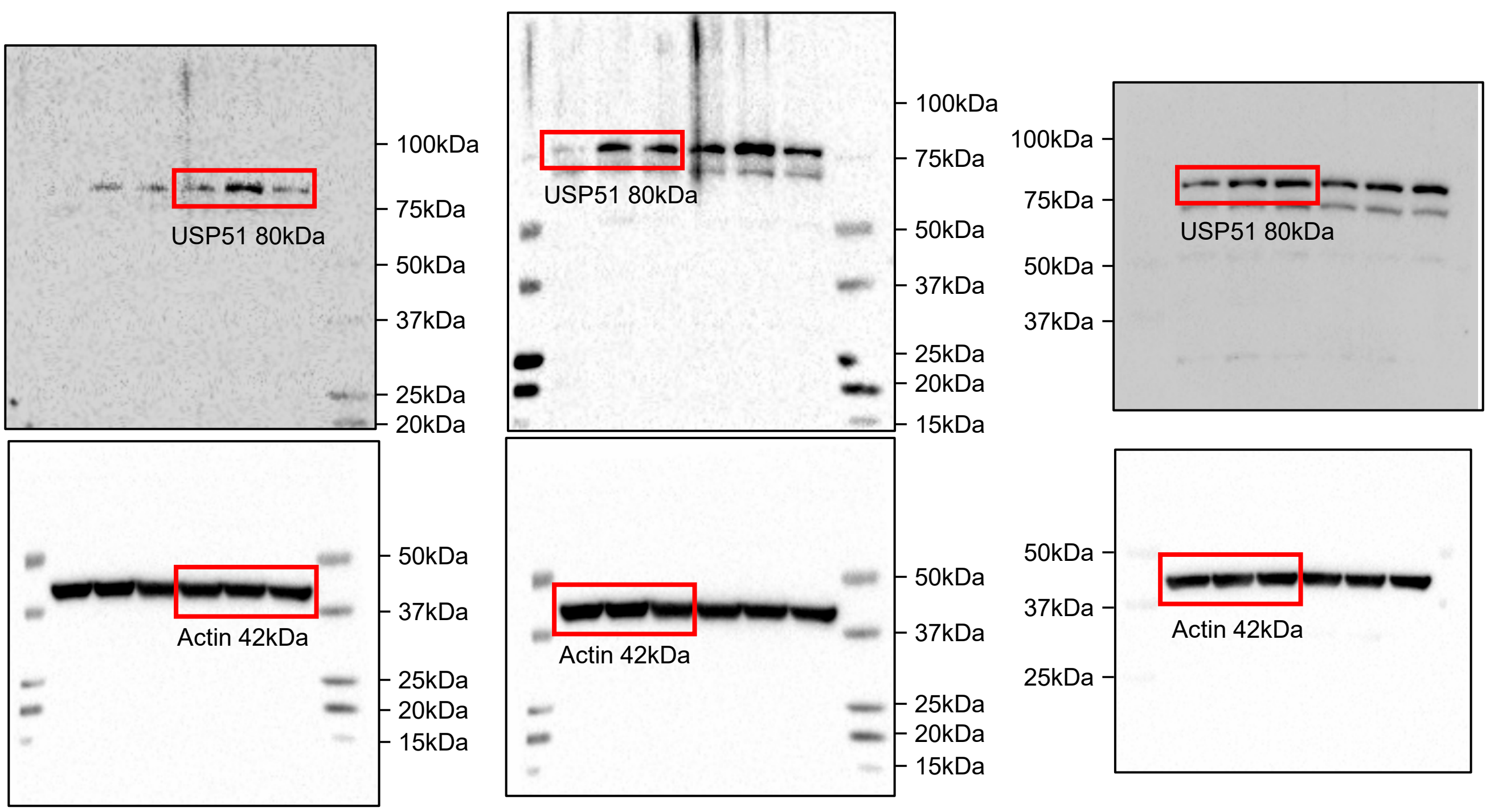

Extended Data Fig. 6d

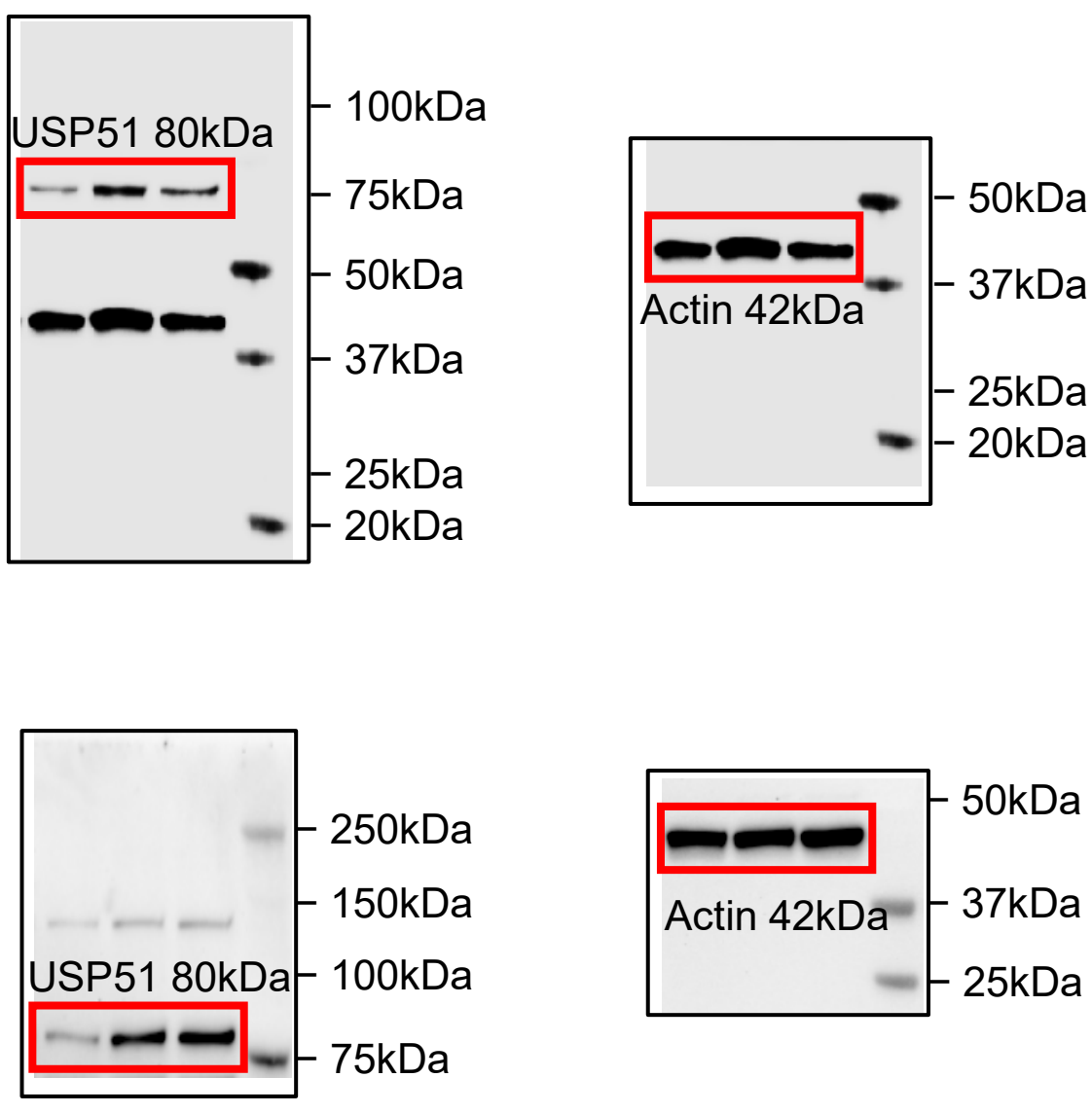

Extended Data Fig. 6e

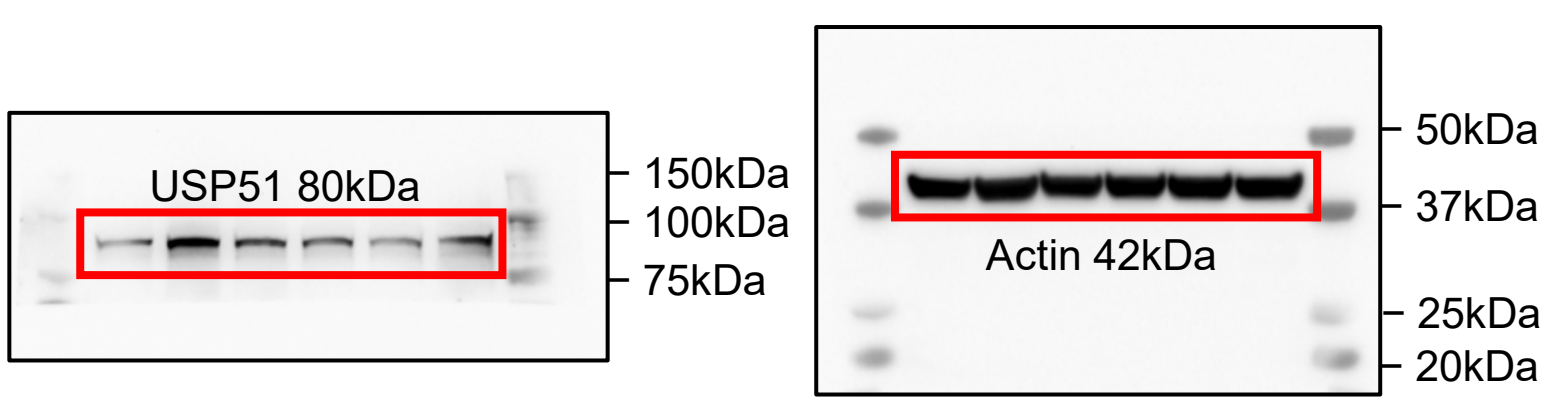

Extended Data Fig. 6f

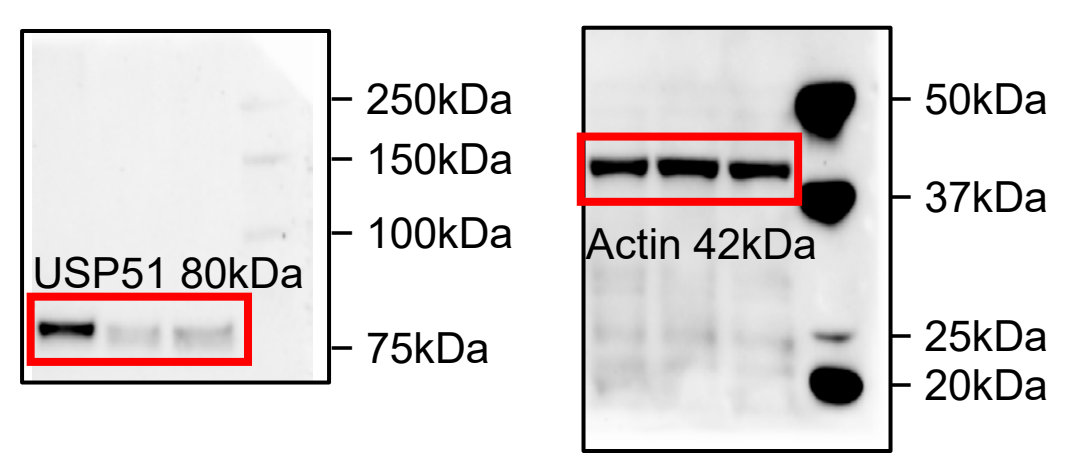

Extended Data Fig. 6g

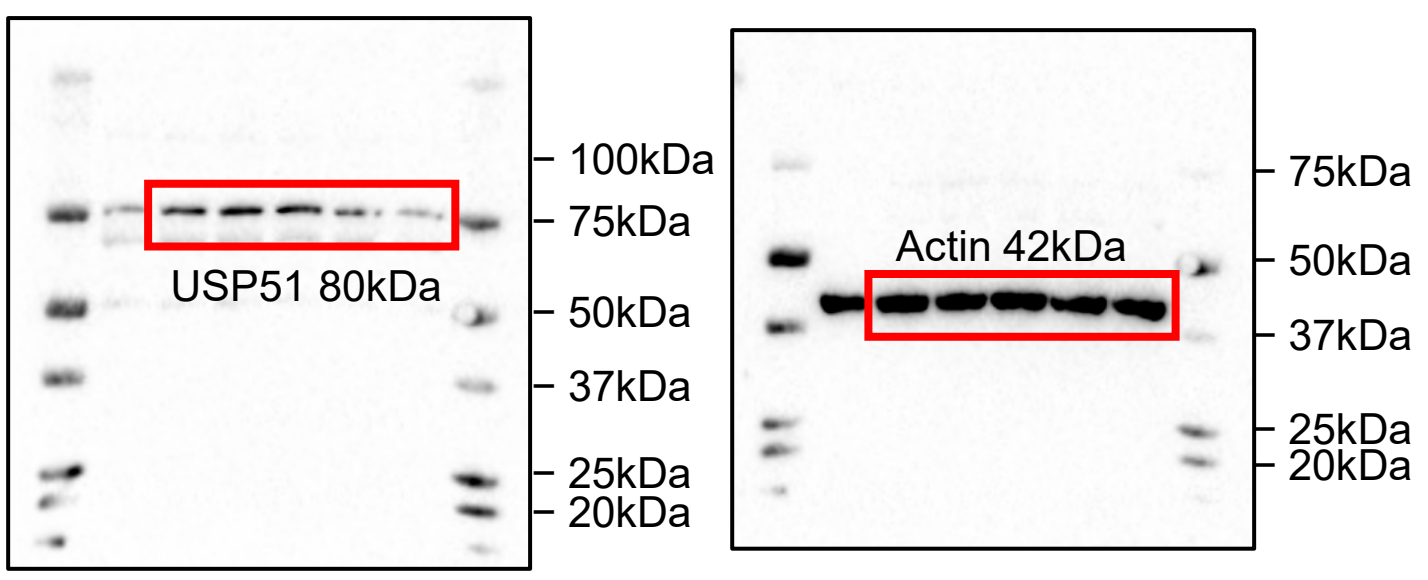

Extended Data Fig. 6h

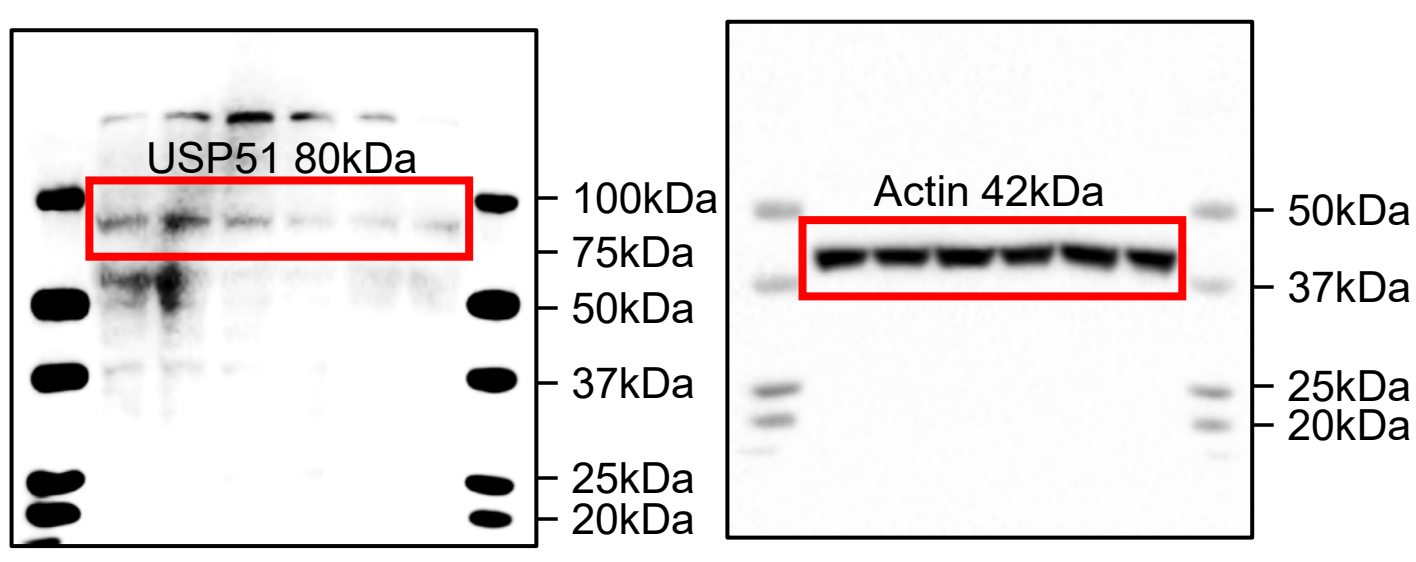

Supplement: Supplementary file 3 [file 43018_2026_1221_MOESM20_ESM.pdf]
