# Supplementary material for: Galanin impairs tumor immunity in glioblastoma by promoting infiltration and ferroptosis resistance of myeloid-derived suppressor cells
Source: Nat Cancer. 2026 Aug 25;7(9):1387–405. doi: 10.1038/s43018-026-01221-3 (PMC13600933; doi:10.1038/s43018-026-01221-3)
Supplement: Supplementary file 4 [file 43018_2026_1221_MOESM2_ESM.pdf]

nature portfolio

Corresponding author(s): Peiwen Chen

Last updated by author(s): Jul 12, 2026

Reporting Summary

Nature Portfolio wishes to improve the reproducibility of the work that we publish. This form provides structure for consistency and transparency in reporting. For further information on Nature Portfolio policies, see our Editorial Policies and the Editorial Policy Checklist.

Statistics

For all statistical analyses, confirm that the following items are present in the figure legend, table legend, main text, or Methods section.

n/a	Confirmed	
☐	☒	The exact sample size (*n*) for each experimental group/condition, given as a discrete number and unit of measurement
☐	☒	A statement on whether measurements were taken from distinct samples or whether the same sample was measured repeatedly
☐	☒	The statistical test(s) used AND whether they are one- or two-sided<br>*Only common tests should be described solely by name; describe more complex techniques in the Methods section.*
☒	☐	A description of all covariates tested
☐	☒	A description of any assumptions or corrections, such as tests of normality and adjustment for multiple comparisons
☐	☒	A full description of the statistical parameters including central tendency (e.g. means) or other basic estimates (e.g. regression coefficient) AND variation (e.g. standard deviation) or associated estimates of uncertainty (e.g. confidence intervals)
☐	☒	For null hypothesis testing, the test statistic (e.g. *F*, *t*, *r*) with confidence intervals, effect sizes, degrees of freedom and *P* value noted<br>*Give P values as exact values whenever suitable.*
☒	☐	For Bayesian analysis, information on the choice of priors and Markov chain Monte Carlo settings
☒	☐	For hierarchical and complex designs, identification of the appropriate level for tests and full reporting of outcomes
☐	☒	Estimates of effect sizes (e.g. Cohen's *d*, Pearson's *r*), indicating how they were calculated

Our web collection on statistics for biologists contains articles on many of the points above.

Software and code

Policy information about availability of computer code

Data collection

Flow cytometry data were collected at Robert H. Lurie Comprehensive Cancer Center Flow Cytometry Core Facility and Cleveland Clinic Lerner Research Institute Flow Cytometry Core using BD FACSymphony A5 Cell Analyzer as specified in MATERIALS AND METHODS.

Western blot and Co-IP bands were imaged under ChemiDoc Touch Imaging System using ImageLab Version 6.1.0 (Bio-Rad).

For GAL protein concentration determination, human plasma and supernatant of GBM cell-derived cell culture medium was measured by ELISA using the Human GAL ELISA Kit (MyBioSource, #MBS2505508) according to the manufacturer's instructions. Biotek Synergy 2 SL Microplate Reader was used to read OD value of the ELISA plate.

For RNA-seq, Oligo-dT-based libraries were prepared and analyzed using the Novaseq 6000 system (paired-end) at The University of Chicago Functional Genomics facility (RRID: SCR_019196).

For lipidomic analyses, lipids in MDSC were were isolated by Metabolomics Platform at the University of Chicago.

For tracking single cell movement, IncuCyte Live Cell Analysis System was used to record cell movement over time.

qRT-PCR was performed using SYBR Green PCR Master Mix (Bio-Rad, #1725275) in CFX Connect Real-Time PCR Detection System with CFX Manger Version 3.1 (Bio-Rad).

Signal of protein with immunofluorescence was captured by using Nikon AX/AX R Confocal Microscope System.

Data analysis

Flow cytometry data were analyzed using FlowJo v10.8.1 as detailed in MATERIALS AND METHODS section.

scRNA-seq data were analyzed using R and Talk2Data.

Bulk RNA-seq: BM-MDSCs were derived from mouse primary bone marrow cells and treated with recombinant GAL protein (10 ng/mL) or CT2A cells-derived CM. RNA was then extracted from the MDSCs and sequenced using a previously established protocol.9,14 Oligo-dT-based libraries were prepared and analyzed using the Novaseq 6000 system (paired-end) at The University of Chicago Functional Genomics facility (RRID: SCR_019196). RNA-seq quality control and mapping were performed on the Galaxy server.7 Raw FASTQ data were trimmed using the Trimmomatic function, and the trimmed reads were then mapped to the mouse (mm10) genome using the HISAT2 program. BAM data from two flowcells were merged using Samtools, and gene expressions were calculated using FeatureCounts. Differential expression (DE) genes were identified using the Limma model, and GSEA analysis was conducted based on the DE table.

IP-LC/MS data analysis: andem mass spectra were extracted. Charge state deconvolution and deisotoping were not performed. All MS/MS samples were analyzed using Mascot (Matrix Science, London, UK; version 2.8.0). Mascot was set up to search database assuming the digestion enzyme trypsin. Mascot was searched with a fragment ion mass tolerance of 0.050 Da and a parent ion tolerance of 10.0 PPM. Carbamidomethyl of cysteine was specified in Mascot as a fixed modification. Deamidated of asparagine and glutamine, oxidation of methionine and acetyl of the n-terminus were specified in Mascot as variable modifications. Scaffold (version Scaffold_5.1.2, Proteome Software Inc., Portland, OR) was used to validate MS/MS based peptide and protein identifications. Peptide identifications were accepted if they could be established at greater than 90.0% probability by the Peptide Prophet algorithm (Keller, A et al Anal. Chem. 2002;74(20):5383-92) with Scaffold delta-mass correction. Protein identifications were accepted if they could be established at greater than 99.0% probability to achieve an FDR less than 1.0% and contained at least 2 identified peptides. Protein probabilities were assigned by the Protein Prophet algorithm (Nesvizhskii, Al et al Anal. Chem. 2003;75(17):4646-58). Proteins that contained similar peptides and could not be differentiated based on MS/MS analysis alone were grouped to satisfy the principles of parsimony.

Lipidomic analyses: After the treatment, MDSCs were collected and washed with PBS. The fresh cell pellets were then sent to the Metabolomics Platform at the University of Chicago for analysis. Lipid was extracted by a modified liquid-liquid extraction method.17 In brief, ice-cold 300 uL methanol +900 uL of methyl-tert-butyl ether was added to the frozen cell pellet followed by sonication (ice-cold water-filled water bath) for 3 mins and mixing the samples at 1000 rpm and 4°C for 15 mins using the Thermomixer. Then ice-cold 300 uL of water was added to induce the phase separation, sonication for 3 mins, mixing the samples for 15 minutes, and centrifuge at 18,000g for 15 mins at 15° C. The upper organic layer (400 uL x2) was dried using Genevac EZ-2.4 elite evaporator and stored at -80 °C. A separate aliquot was used to acquire positive and negative ionization mode data. The dried lipid extract was re-suspended in 60 uL of 3/2/1 isopropanol/acetonitrile/water(V/V/V) at room temperature, sonicated for 3 mins, vortex for 15 mins at 2000 rpm and 15 °C and 30 uL of supernatant was transferred to LC-MS vial. The remaining extract was used to generate the PooledQC sample. Study samples were injected in random order and bracketed in between the pooled samples.Lipid species chromatography separation was performed on a Thermo Scientific Accucore C30 (2.1x150 mm, 2.6 um) column connected to a Vanquish Horizon UHPLC system and IQ-X tribrid mass spectrometer. The mobile phase A(MPA) was 60/40 acetonitrile/water, 10 mM ammonium formate+0.1% formic acid and MPB was 89.1/9.9/0.99 isopropanol/acetonitrile/water, 10 mM ammonium formate +0.1% formic acid. The chromatographic gradient was 0 min: 30% B, 2.00 mins: 43% B, 2.1 mins: 55% B, 12.00 mins: 65% B, 18.00 mins: 85% B, 20.00 mins: 100% B, 25.00 mins:100% B, 25.1 mins: 30% B and 30.00 mins: 30% B. The column temperature, injection volume, and flow rate were 45 °C, 4 uL, and 0.26 mL/min, respectively. MS1 parameters were as follows: spray voltage: 3500 V for positive ionization and 2500 V for negative ionization modes, sheath gas: 40, auxiliary gas: 10, sweep gas: 1, ion transfer tube temperature: 300 °C, vaporizer temperature: 350 °C, orbitrap resolution: 120 K, scan range(m/z): 200-2000 for pos, 200-2000 for neg, RF lens (%): 60, automatic gain control (AGC) target: 50%, and a maxIT of 100 milliseconds (ms). Quadrupole isolation and Internal calibration using Easy IC were enabled. Lipids were identified by performing the MS2 and MS3 experiments in the orbitrap mass analyzer. A comprehensive data-dependent HCD MS2 experiment with conditional CID MS2 and MS3 data-acquisition strategy was applied for the in-depth characterization of lipid species. Lipid fragmentation was obtained by first MS1 data acquisition in full scan mass range (200-2000) followed by the data-dependent (dd) MS2 with the normalized stepped HCD collision energy (%) at 25, 30, 35, OT-30K resolution, maxIT-54 ms. If the HCD fragmentation had the fragment ion- 184.0733 m/z (phosphocholine head group) then the same ions were subjected either to ddMS2 CID (fixed collision energy-32%, activation time-10 ms & Q-0.25, 30K resolution, 100% normalized AGC target and maxIT-54 ms) or CID MS3 scans (fixed collision energy-35%, activation Q-0.25) triggered on the top 3 most intense ions that lost neutral fatty acids plus ammonia (only for triacylglycerols). For the negative ionization mode, only data-dependent stepped HCD(20,30,40) MS2 was performed in orbitrap analyzer at 30 K resolution, AGC target of 100% and max IT of 54 ms. A total of six injections were made to generate fragmentation data using the AcquireX workflow on the pooled QC samples. The Thermo Scientific LipidSearchTM software version 5.0 was used to generate the list of identified lipid (Precursor tolerance ± 3 ppm, production tolerance ±5.0 ppm, product threshold- 1.0). [M+H] adduct was used to identify and quantify HexCer,SM, SPH,MePC, CoQ, AcylCarnitine,LPC,LPE,PC and PE species while [M+NH] was for the TG, DG, cholesteryl ester species. These identified lipid species were quantify using the Compound Discoverer 3.3 and Skyline software.

The number of transferred cells in transwell migration assay and immunofluorescence signal was counted and measured using ImageJ Version 1.53t (NIH, Bethesda, ML).

For tracking single cell movement, the time-lapse images were reconstructed and analyzed using TrackMate. The speed of each individual cell was calculated by processing images with LoG detector and Linear Assignment Problem (LAP) tracker.

Statistical analysis was conducted using GraphPad Prism 9 (GraphPad Software, USA). In vitro and in vivo measurement data were presented as the means ± SEM or SD. Comparison between two groups was evaluated using Student's t-test, and multiple comparisons among groups were evaluated using a one-way ANOVA test in Tukey's method. The survival analysis for animal models was performed using Log-rank (Mantel-Cox) test. Correlation analysis was performed using the Pearson test to determine the R and P values.

Illustrations were created with BioRender.

For manuscripts utilizing custom algorithms or software that are central to the research but not yet described in published literature, software must be made available to editors and reviewers. We strongly encourage code deposition in a community repository (e.g. GitHub). See the Nature Portfolio guidelines for submitting code & software for further information.

Data

Policy information about availability of data

All manuscripts must include a data availability statement. This statement should provide the following information, where applicable:

- Accession codes, unique identifiers, or web links for publicly available datasets
- A description of any restrictions on data availability
- For clinical datasets or third party data, please ensure that the statement adheres to our policy

The sequencing datasets generated in this study have been deposited in the Gene Expression Omnibus (GEO) under the accession number GSE284502 [https://www.ncbi.nlm.nih.gov/geo/query/acc.cgi?acc=GSE284502]. The lipidomics data are available through the Metabolomics Workbench under accession number ST004928 [https://doi.org/http://dx.doi.org/10.21228/M8BS0X]. The neuropeptide genes encoding secreted proteins are available in the HGNC database [https://www.genenames.org/data/genegroup/#!/group/1902]. The TCGA GBM data are available through the Genomic Data Commons (GDC) Data Portal [https://portal.gdc.cancer.gov/] and can also be accessed via GlioVis [http://gliovis.bioinfo.cnio.es] and cBioPortal [https://www.cbioportal.org]. The publicly available single-cell sequencing data of patients with GBM tumors are available in the GEO database under accession codes GSE182109 [https://www.ncbi.nlm.nih.gov/geo/query/acc.cgi?acc=GSE182109] and GSE GSE135045 [https://www.ncbi.nlm.nih.gov/geo/query/acc.cgi?acc=GSE135045]. The brain TIME dataset used in this study is publicly available at [https://joycelab.shinyapps.io/braintime]. Source data are included with this publication. Additional data supporting the findings of this study are available within the article, its supplementary information, Source data, or upon request from the corresponding author.

Human research participants

Policy information about studies involving human research participants and Sex and Gender in Research.

Reporting on sex and gender	Samples from 81 individuals have been used in this study. Patient information is listed in Supplementary Table S1.
Population characteristics	57 new diagnosed GBM patients and 24 recurrent GBM patients.
Recruitment	GBM patient peripheral blood plasma samples (n=64) were collected from the Northwestern Central Nervous System Tissue Bank (NSTB). Patient tumor samples (n=20) from surgically-resected IDH-WT GBMs were collected at the NSTB. All patients were diagnosed according to the WHO diagnostic criteria by neuropathologist Dr. Craig Horbinski.
Ethics oversight	According to The George Washington University Institutional Review Board and based on the guidelines from the Office of Human Research Protection, the conducted research meets the criteria for exemption #4 (45 CFR 46.101(b) Categories of Exempt Human Subjects Research) and does not constitute human research.

Note that full information on the approval of the study protocol must also be provided in the manuscript.

Field-specific reporting

Please select the one below that is the best fit for your research. If you are not sure, read the appropriate sections before making your selection.

☒ Life sciences ☐ Behavioural & social sciences ☐ Ecological, evolutionary & environmental sciences

For a reference copy of the document with all sections, see nature.com/documents/nr-reporting-summary-flat.pdf

Life sciences study design

All studies must disclose on these points even when the disclosure is negative.

Sample size	No statstical method was used to predetermine sample size. Sample size was chosen based on prior experience and prior published studies with similar layout (see detailed information in the Method section with cited references, including 1. Chen, P. et al. Circadian Regulator CLOCK Recruits Immune-Suppressive Microglia into the GBM Tumor Microenvironment. Cancer Discovery 10, 371-381 (2020). 2. Xuan, W. et al. Circadian Regulator CLOCK Drives Immunosuppression in Glioblastoma. Cancer Immunology Research 10, 770-784 (2022). 3. Chen, P. et al. Symbiotic Macrophage-Glioma Cell Interactions Reveal Synthetic Lethality in PTEN-Null Glioma. Cancer Cell 35, 868-884.e866 (2019).
Data exclusions	No data were excluded from the analysis. Datasets used for the specific analyses are reported in the Method section.
Replication	The number of replicates for each experiment is indicated in figure legends. Within each experimental group the reproducibility was successful, although a degree of variability was detected due to inter- individual diversity.
Randomization	We did not perform randomization of study participants or samples within each group because not relevant /needed for this study. The intracranial xenograft tumor model was used throughout this study. Briefly, the animals were assigned into different groups after a week of intracranial injection and received treatments. Since no sign can be observed for mice in the first week after intracranial injection and the animals are genetically identical, housed under identical conditions, and no baseline differences were expected between groups, randomization is not performed.

Blinding

Investigators were not blinded to group allocation because not relevant /needed for this study. Data reported for mouse experiments and all other experiments were not subjective but rather based on quantitative analyses.

Reporting for specific materials, systems and methods

We require information from authors about some types of materials, experimental systems and methods used in many studies. Here, indicate whether each material, system or method listed is relevant to your study. If you are not sure if a list item applies to your research, read the appropriate section before selecting a response.

Materials & experimental systems

n/a	Involved in the study	
☐	☒	Antibodies
☐	☒	Eukaryotic cell lines
☒	☐	Palaeontology and archaeology
☐	☒	Animals and other organisms
☒	☐	Clinical data
☒	☐	Dual use research of concern

Methods

n/a	Involved in the study	
☒	☐	ChIP-seq
☐	☒	Flow cytometry
☒	☐	MRI-based neuroimaging

Antibodies

Antibodies used

GAL; Invitrogen; Cat#PA5-95500; RRID: AB_2807302
β-ACTIN; Cell Signaling; Cat#3700S; RRID:AB_2242334
ERa; Invitrogen; Cat#MA1-80216; RRID: AB_930763
ERb; Invitrogen; Cat#PA1-311; RRID: AB_325597
Histone H3; Cell Signaling; Cat#9715S; RRID: AB_331563
USP51; Invitrogen; Cat#PA5-68358; RRID: AB_2691902
Ubiquitin; Cell Signaling; Cat#43124S; RRID: AB_2799235
CD69 (for Immunofluorescence analysis); Santa Cruz; Cat#sc-373799; RRID: AB_10915746
CD11b (for Immunofluorescence analysis); Abcam; Cat#ab8878; RRID: AB_306831
F4/80 (for Immunofluorescence analysis); Cell Signaling; Cat#30325S; RRID: AB_2798990
CD14 (for Immunofluorescence analysis); Abcam; Cat#ab181470; RRID: AB_2725781
CD66b (for Immunofluorescence analysis); , NOVUS, Cat#NB100-77808; RRID: RRID:AB_2077866
4-HNE (for Immunofluorescence analysis); Invitrogen; Cat#MA5-27570; RRID: AB_2735095
FITC-conjugated CD4; Cell Signaling; Cat#96127; RRID: AB_2943473
FITC-conjugated CD8; Cell Signaling; Cat#35467; RRID: AB_2799078
Anti-CD16/CD32 cocktail; BioLegend; Cat#101320; RRID: AB_1574975
PE/Cy7 anti-mouse/human CD11b; BioLegend; Cat#101216; RRID: AB_312799
Alexa Fluor 700 anti-mouse Ly6C; BioLegend; Cat#128024; RRID: AB_10643270
PE anti-mouse CD68; BD Bioscience; Cat#566386; RRID: AB_2744448
FITC anti-mouse Ly6G; BioLegend; Cat#127606; RRID: AB_1236494
GALR1; G-Biosciences; Cat#ITT1844; RRID: AB_3662131
GALR2; G-Biosciences; Cat#ITT1845; RRID: AB_3662132
GALR3; Abcam; Cat#ab190694; RRID: AB_3662133
AF488 anti-mouse CD3; BioLegend; Cat#100210; RRID: AB_389301
PerCP/Cyanine5.5 anti-mouse CD45 Antibody; BioLegend; Cat#103132; RRID: AB_893340
PE/Cy7 anti-mouse CD69; BioLegend ;Cat#104512; RRID: AB_493564
BV711 anti-mouse CD8; BioLegend ;Cat#100747; RRID: AB_11219594
BUV395 anti-mouse CD4; BD Bioscience; Cat#740208; RRID: AB_2734761
IgG isotype control; Cell Signaling; Cat#2729; RRID: AB_1031062
Anti-mouse IgG, HRP-Linked; Cell Signaling; Cat#7076S; RRID:AB_330924
Anti-rabbit IgG, HRP-linked; Cell Signaling; Cat#7074S; RRID:AB_2099233
Anti-rabbit IgG, Alexa Fluor® 594 Conjugate; Cell Signaling; Cat#8889S; RRID:AB_2716249
Anti-rabbit IgG, Alexa Fluor ® 488 Conjugate; Cell Signaling; Cat#4412S; RRID:AB_1904025
Anti-mouse IgG, Alexa Fluor® 594 Conjugate; Cell Signaling; Cat#8890S; RRID:AB_2714182
Anti-mouse IgG, Alexa Fluor® 488 Conjugate; Cell Signaling; Cat#4408S; RRID:AB_10694704
Anti-rat IgG, Alexa Fluor 555 conjugate; Invitrogen; Cat#A48270; RRID: AB_2896336
Anti-rat IgG, Alexa Fluor 647 conjugate; Invitrogen; Cat#A21247; RRID: AB_141778
Anti–PD-1; BioXCell; Cat#BE0146; RRID: AB_10949053
Anti–Gr1; BioXCell; Cat#BE0075; RRID: AB_10312146
Anti–IgG; BioXCell; Cat#BE0091; RRID: AB_1107773

Validation

All antibodies used in this study were purchased from BD bioscience, Biolegend, Cell Signaling Technology, Abcam, Invitrogen, or Millipore. All antibodies used are characterized and validated by providers and are suitable for flow-cytometric analyses, western blot, immunofluorescence analysis, or Co-IP.

From BioLegend: Specificity testing of 1-3 target cell types with either single- or multi-color analysis (including positive and negative cell types). Once specificity is confirmed, each new lot must perform with similar intensity to the in-date reference lot. Brightness (MFI) is evaluated from both positive and negative populations. Each lot product is validated by QC testing with a series of titration

dilutions. Those listed under "verified" reactivity indicate species that have been tested and confirmed to react in-house by BioLegend.

From BD Biosciences: The production process underwent stringent testing and validation to assure that it generates a high-quality conjugate with consistent performance and specific binding activity. However, verification testing has not been performed on all conjugate lots. The specificity is confirmed using multiple methodologies that may include a combination of flow cytometry, immunofluorescence, immunohistochemistry or western blot to test staining on a combination of primary cells, cell lines or transfectant models.

From Abcam: Knock-out validation has been performed on primary antibodies.

From Cell Signaling Technology: Hallmarks of Antibody Validation™, six complementary strategies were used to determine the functionality, specificity, and sensitivity of an antibody in any given assay. The validation was adapted the work by Uhlen, et. al., ("A Proposal for Validation of Antibodies." Nature Methods (2016)).

Eukaryotic cell lines

Policy information about cell lines and Sex and Gender in Research

Cell line source(s)	CD8+ primary mouse cell was derived from the spleen of male and female 8-weeks old mice.<br><br>Mouse primary BM-MDSC was derived from mouse bone marrow.<br><br>Human primary MDSC was derived from human PBMC. Health human whole peripheral blood was obtained from STEMCELL Technologies (# 70508.1).<br><br>The CT2A cell line was derived from a sub-cutaneous, non-metastatic murine glioma. CT2A cells were purchased from MilliporeSigma (#SCC194).<br><br>Mouse 005 GSCs were provided by Dr. Samuel D. Rabkin (Massachusetts General Hospital), respectively.<br><br>U87 (#HTB-14) cell line was purchased from the American Type Culture Collection (ATCC).<br><br>U251 cell line was purchased from MilliporeSigma (#09063001).<br><br>293T cells were purchased from ATCC (#CRL-3216). 293T is an epithelial-like cell that was isolated from the kidney of a patient.<br><br>The JURKAT cell line was established from the peripheral blood of a 14 year old boy by Schneider et al., and was originally designated JM.<br><br>Raw264.7 cells were purchased from ATCC (#TIB-71). RAW 264.7 is an adherent cell line isolated from a mouse tumor that was induced by Abelson murine leukemia virus.
Authentication	All human cell lines have been validated and authenticated through fingerprinting by the MD Anderson Cell Line Core Facility. Mouse primary cells and GSCs were not authenticated.
Mycoplasma contamination	All cell lines are tested negative with mycoplasma contamination.
Commonly misidentified lines (See ICLAC register)	No commonly misidentified cells were used.

Animals and other research organisms

Policy information about studies involving animals; ARRIVE guidelines recommended for reporting animal research, and Sex and Gender in Research

Laboratory animals	C57BL/6 (#0000664), nude (#007850) and ERa-KO (#026176) at 6 weeks of age were purchased from the Jackson Laboratory.<br><br>GALR2 KO mice were generated through a targeted mutation in the intron of the Galr2 gene, resulting in premature termination of translation. The original mouse line was obtained from Lexicon Genetics (USA)9 ref and provided by Marina Picciotto's lab at Yale University to the laboratory of Barbara Kofler. The line, initially on a C57BL/6J genetic background was backcrossed to the C57BL/6N background in the laboratory of Prof. Barbara Kofler. GALR3 KO mice were originally obtained from the European Mouse Mutant Archive (EMMA) and generated via homologous recombination, targeting both coding exons of the Galr3 gene. The line was backcrossed to the C57BL/6N background for at least 7 additional generations. To maintain genetic stability and ensure reproducibility in experiments, KO animals used in this study were bred from homozygous parents. Additionally, the genetic background was refreshed by crossing back to C57BL/6N (Charles River, Germany) every 8–10 generations. The genotype was confirmed by PCR analysis of tail biopsies.<br><br>Mice were grouped by 5 animals in large plastic cages and were maintained under pathogen-free conditions. Animals were housed in temperature and humidity controlled rooms with 12:12 light/dark cycles with Teklad LM-485 sterilizable diet.
Wild animals	No wild animals were used in the study.

Reporting on sex	Both male and female animals were used in this study. Sex was not considered as an experimental factor in this study.
Field-collected samples	No field collected samples were used in the study.
Ethics oversight	All animal experiments were performed with the approval of the Institutional Animal Care and Use Committee (IACUC) at Cleveland Clinic and Northwestern University<br>Animals are under protocols 00003354, IS00017004, and IS00015772.

Note that full information on the approval of the study protocol must also be provided in the manuscript.

Flow Cytometry

Plots

Confirm that:

☒ The axis labels state the marker and fluorochrome used (e.g. CD4-FITC).

☒ The axis scales are clearly visible. Include numbers along axes only for bottom left plot of group (a 'group' is an analysis of identical markers).

☒ All plots are contour plots with outliers or pseudocolor plots.

☒ A numerical value for number of cells or percentage (with statistics) is provided.

Methodology

Sample preparation	For membrane protein staining, the single-cell suspensions were washed with PBS and then incubated with fixable viability dye (Invitrogen, #5211229035) on ice for 10 min. Following washing with PBS, cells were incubated with the anti-CD16/CD32 cocktail (BioLegend, #103132) in 2% BSA PBS to block Fc receptors for 30 min on ice. Different antibody combinations were added to cell suspensions for 30 min on ice. ERa antibody or GALR1/2/3 antibody was added to cell suspension with corresponding antibody combinations for 30 min on ice, followed by incubation of goat anti-rabbit IgG cross-adsorbed secondary antibody (AF594) for 30 min. After washing in PBS, cells were fixed in fixation buffer (BioLegend, #420801) overnight.<br><br>Intracellular protein staining was performed separately or followed by cell surface marker staining. Cells were fixed in fixation buffer at room temperature for 20 min. After washing in permeabilization buffer (0.1% Triton X-100 in PBS) for two times, cells were incubated with antibodies for 20 min at room temperature. Then cell suspensions were rewashed again in permeabilization buffer before testing.
Instrument	Samples were read in BD FACSymphony flow cytometer.
Software	Samples were analyzed in FlowJo v10.8.1.
Cell population abundance	Fluorescence-activated cell sorting (FACS) was used to subtype mouse BM-MDSCs.<br><br>Magnetic-activated cell sorting (MACS) system was used to generate MDSCs from mouse bone marrow (BM), Raw264.7 monocytes, and human peripheral blood
Gating strategy	CD11b+CD68-Ly6GlowLy6Chigh mMDSCs<br>CD11b+CD68-Ly6GhighLy6Clow gMDSCs<br>CD45+CD3+ splenic T cells<br>CD45+CD3+CD8+ CD8 T cells<br>CD45+CD3+CD8+CD69+ activated CD8 T cells<br>CD45+CD3+CD4+CD69+ activated CD4 T cells<br>CD45+CD3+CD4+ CD4 T cells

☒ Tick this box to confirm that a figure exemplifying the gating strategy is provided in the Supplementary Information.
