# Supplementary material for: Galanin impairs tumor immunity in glioblastoma by promoting infiltration and ferroptosis resistance of myeloid-derived suppressor cells
Source: Nat Cancer. 2026 Aug 25;7(9):1387–405. doi: 10.1038/s43018-026-01221-3 (PMC13600933; doi:10.1038/s43018-026-01221-3)
Supplement: Supplementary file 5 [file 43018_2026_1221_MOESM5_ESM.pdf]

**Fig. 1j**

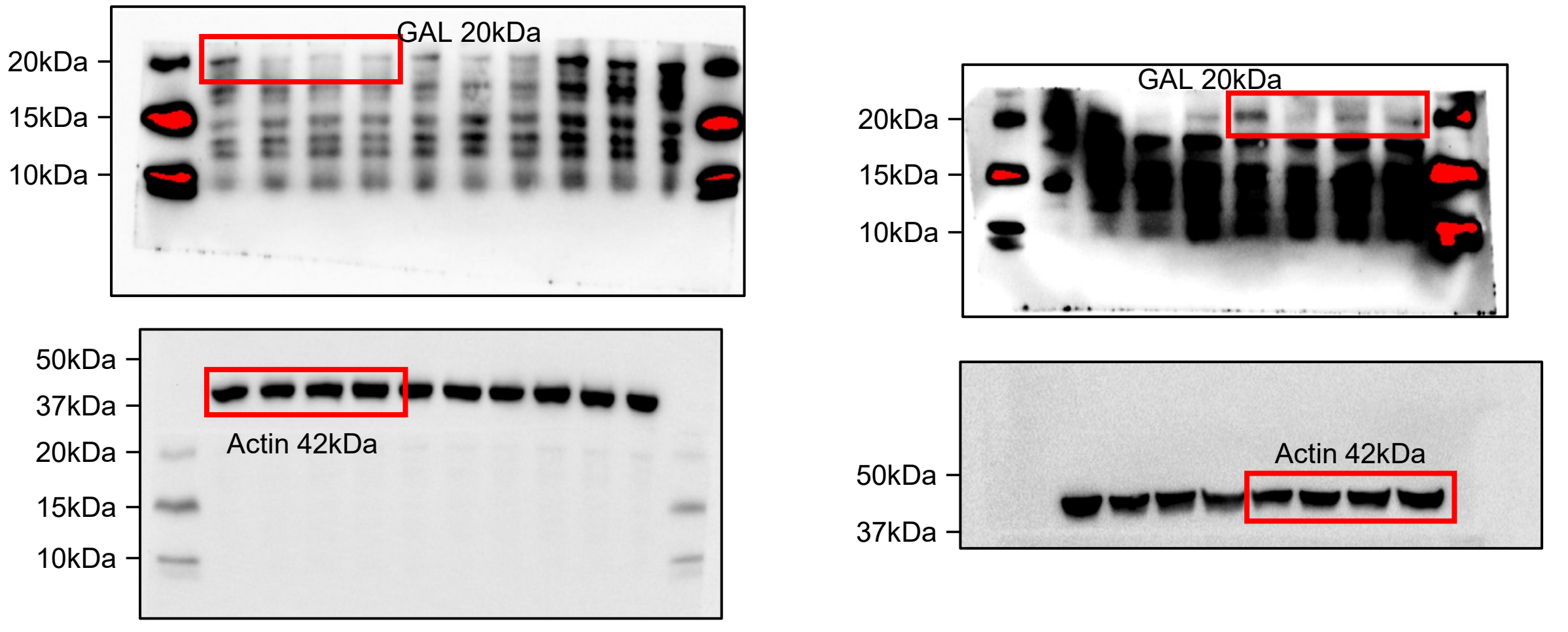
GAL 20kDa
20kDa
15kDa
10kDa
50kDa
37kDa
20kDa
15kDa
10kDa
Actin 42kDa
GAL 20kDa
20kDa
15kDa
10kDa
Actin 42kDa
50kDa
37kDa

**Fig. 1l**

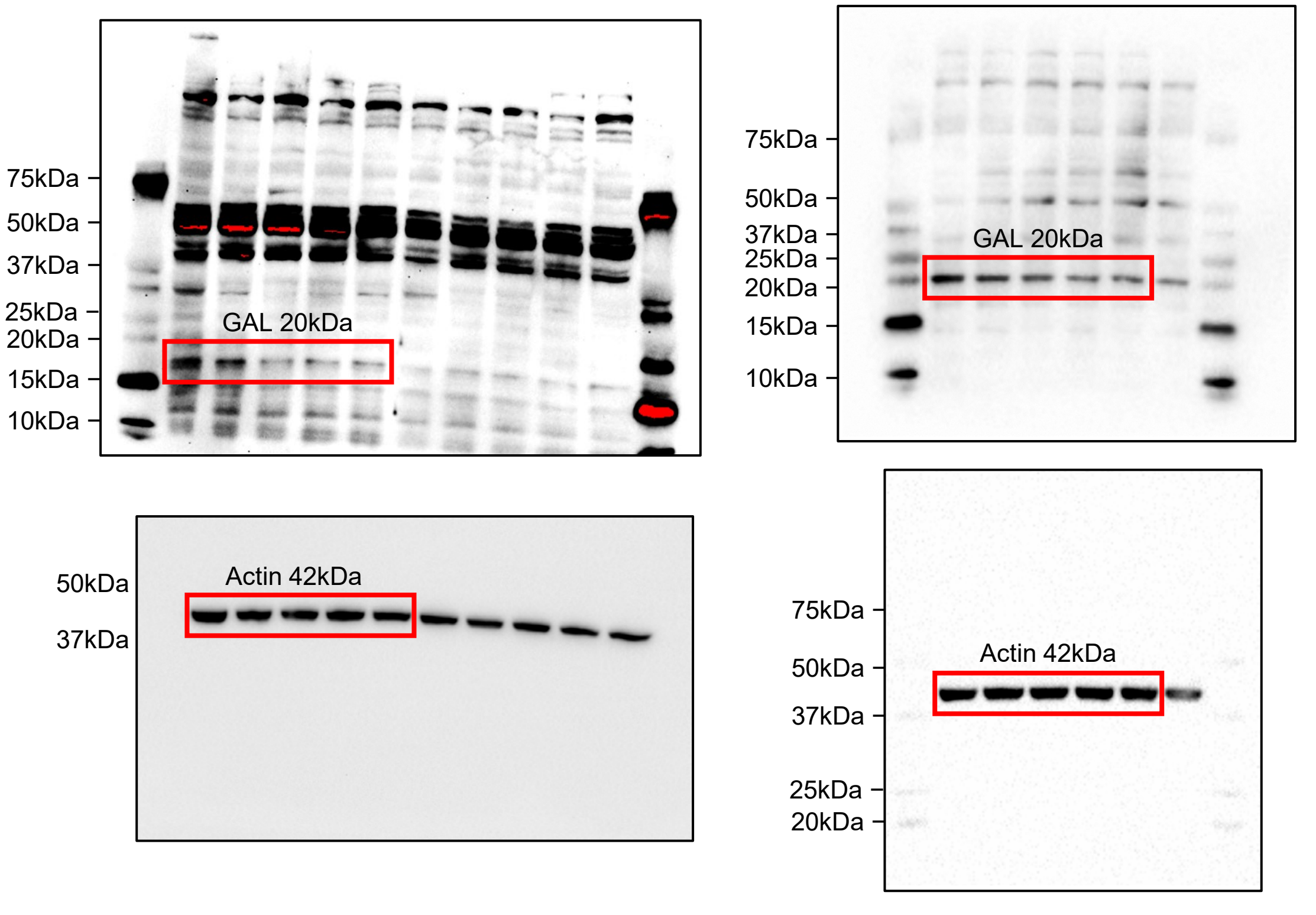
75kDa
50kDa
37kDa
25kDa
20kDa
15kDa
10kDa
GAL 20kDa
75kDa
50kDa
37kDa
25kDa
20kDa
15kDa
10kDa
GAL 20kDa
Actin 42kDa
50kDa
37kDa
75kDa
50kDa
37kDa
25kDa
20kDa
Actin 42kDa
