# Supplementary material for: Galanin impairs tumor immunity in glioblastoma by promoting infiltration and ferroptosis resistance of myeloid-derived suppressor cells
Source: Nat Cancer. 2026 Aug 25;7(9):1387–405. doi: 10.1038/s43018-026-01221-3 (PMC13600933; doi:10.1038/s43018-026-01221-3)

Fig. 4b

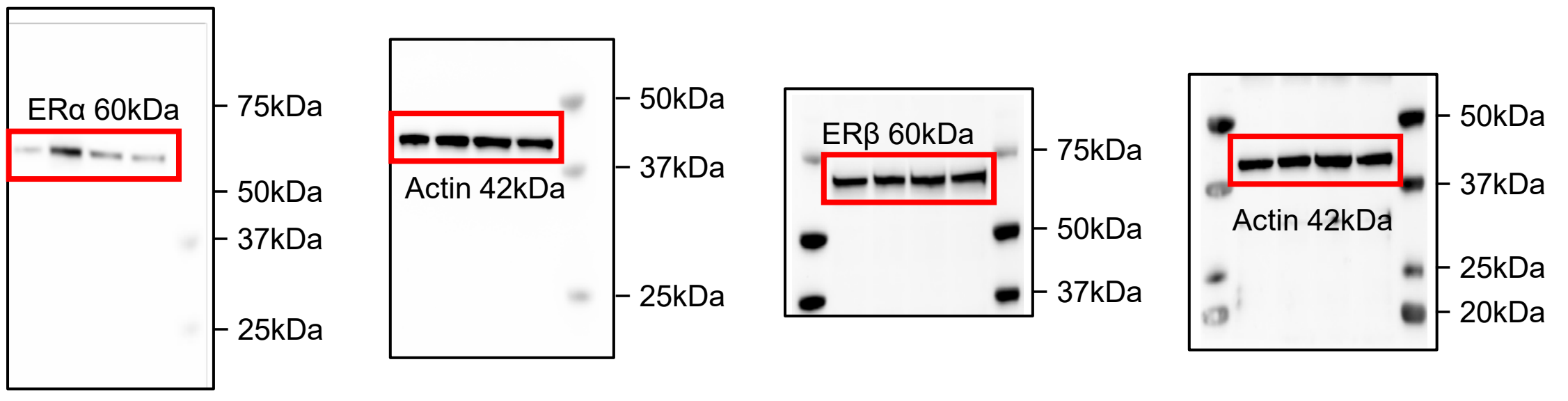

Fig. 4c

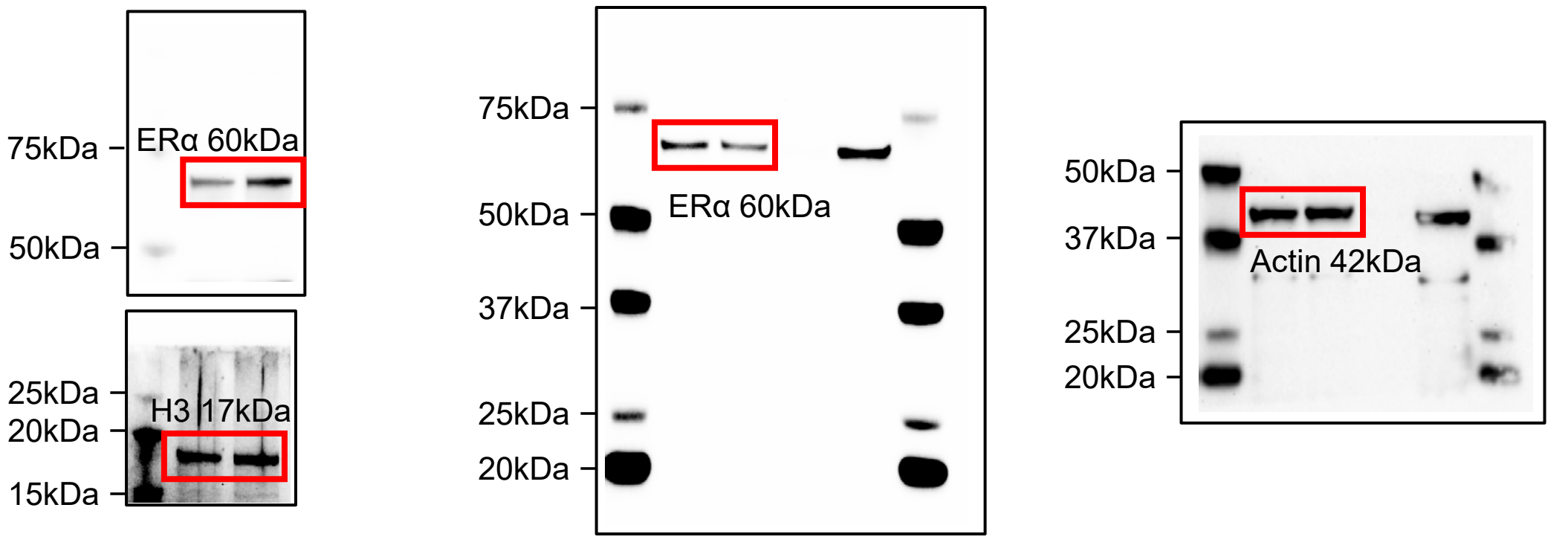

Fig. 4d

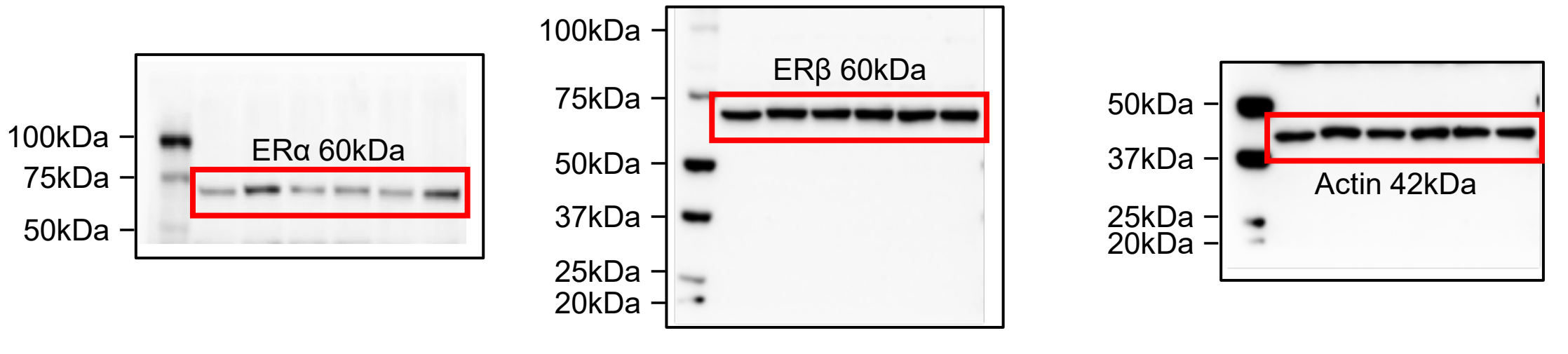

Fig. 4e

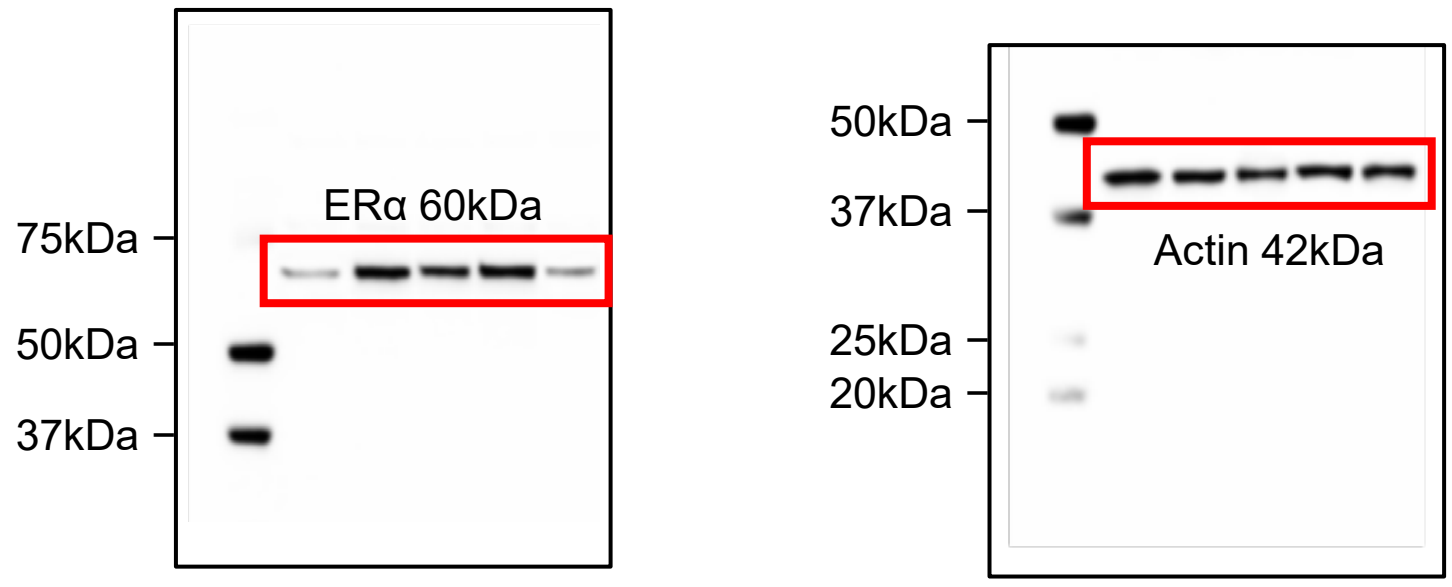

Fig. 4m

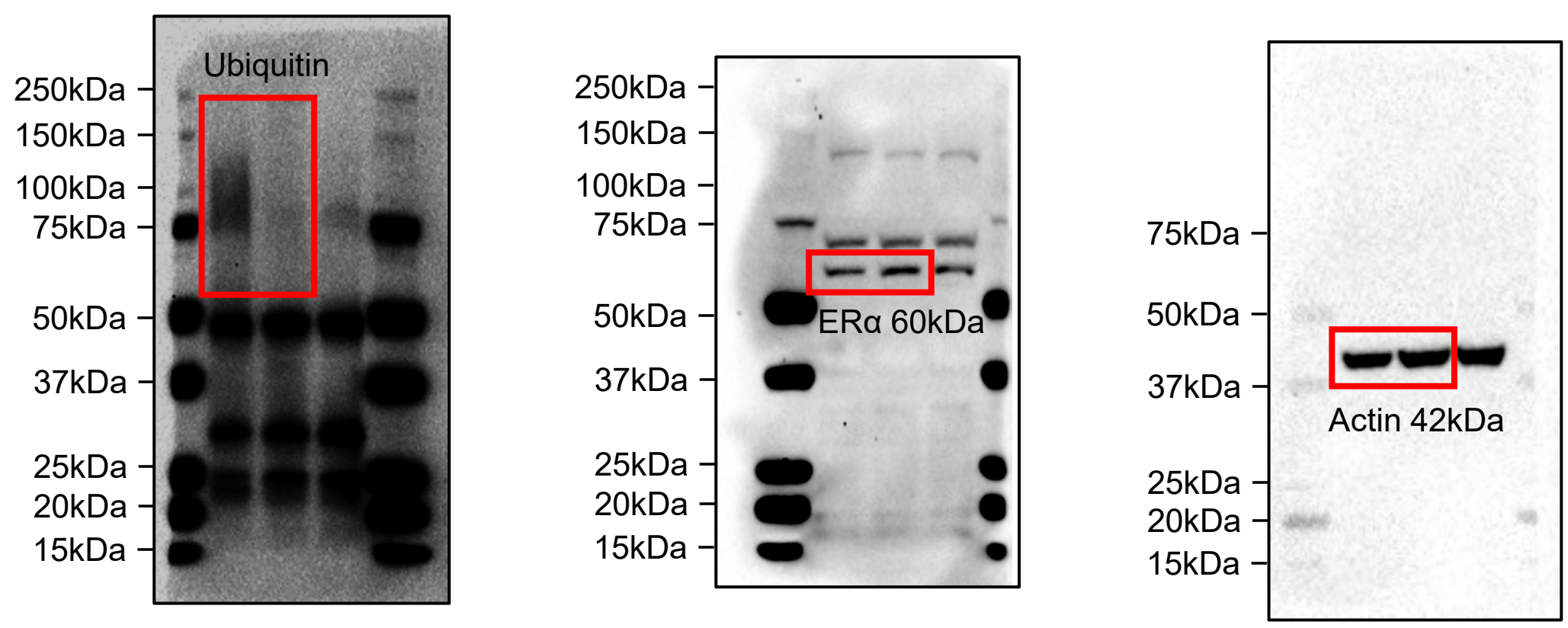

Fig. 4n

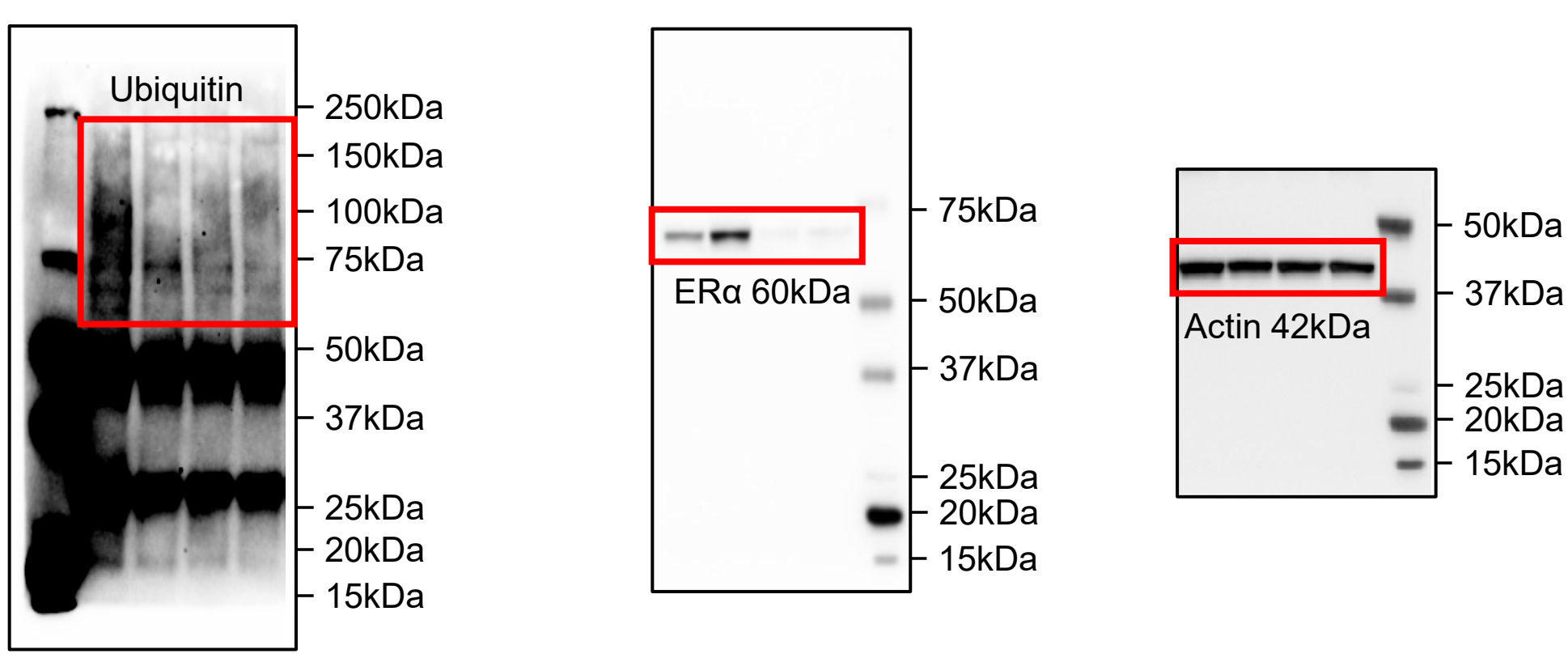

Fig. 4p

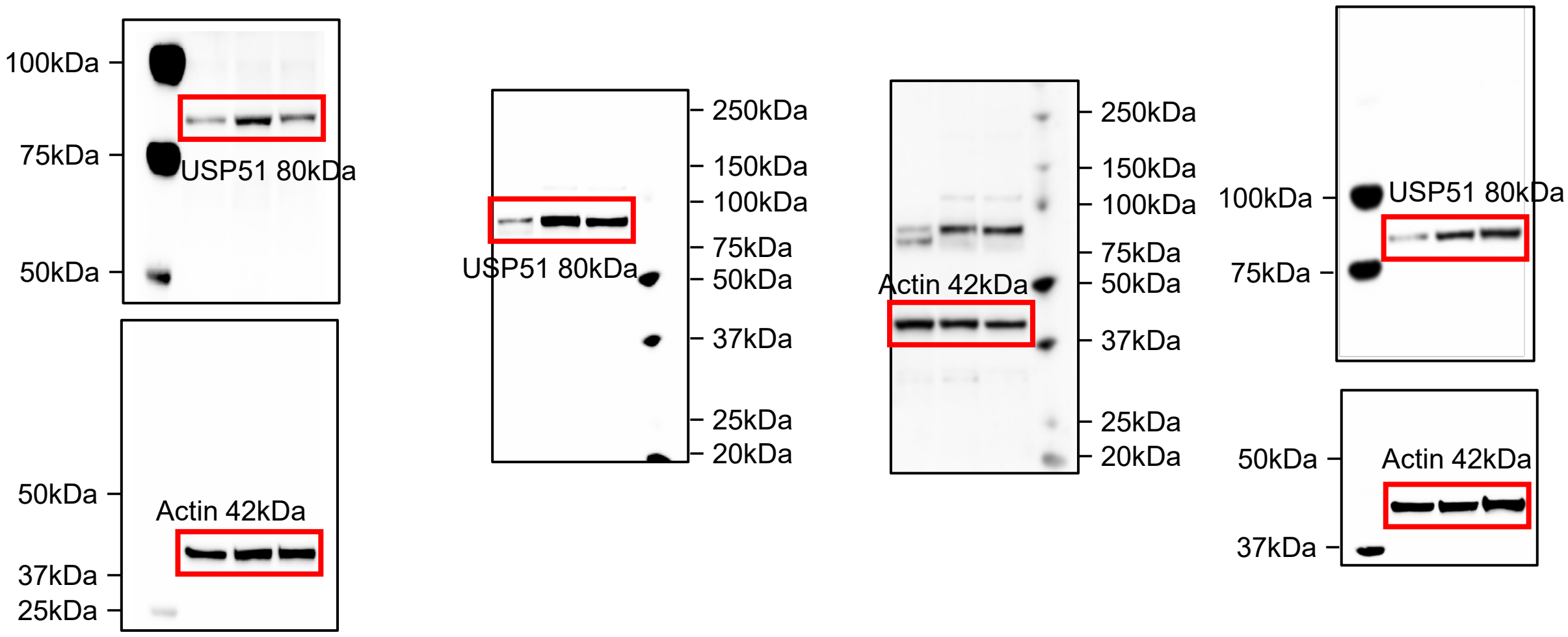

Fig. 4q

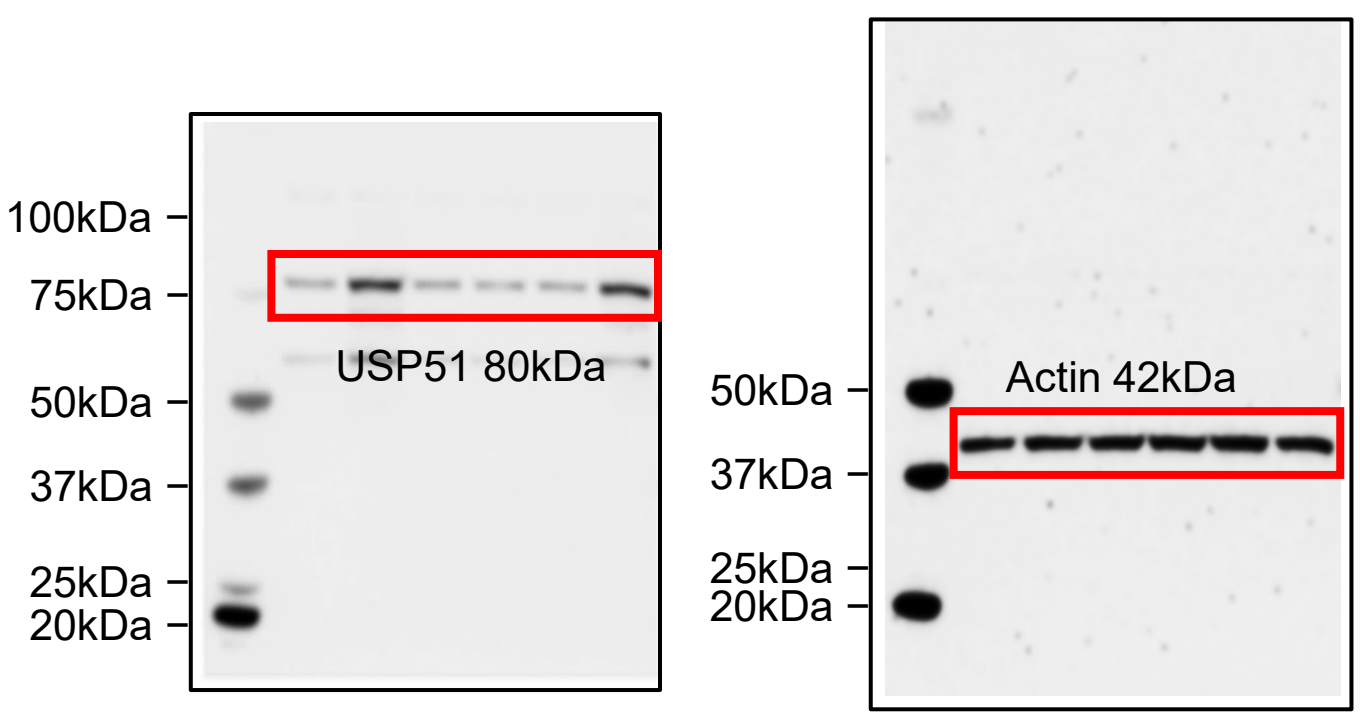

Fig. 4r

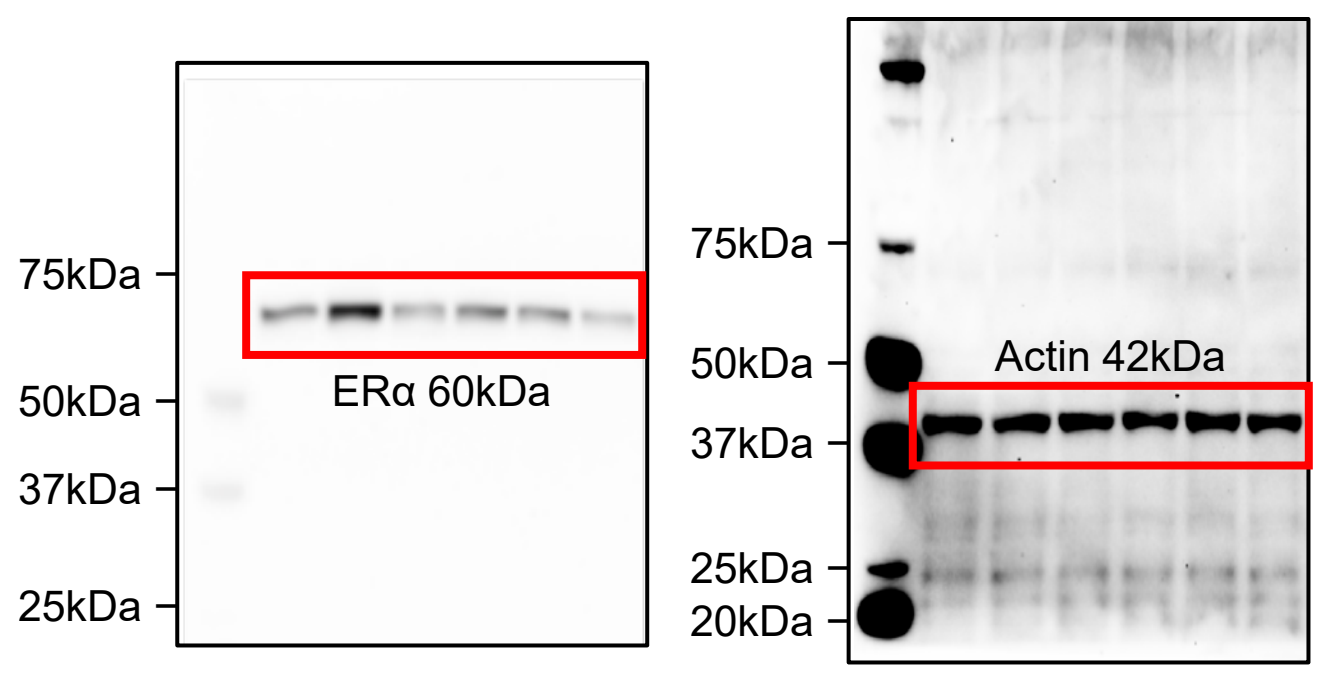

Fig. 4s

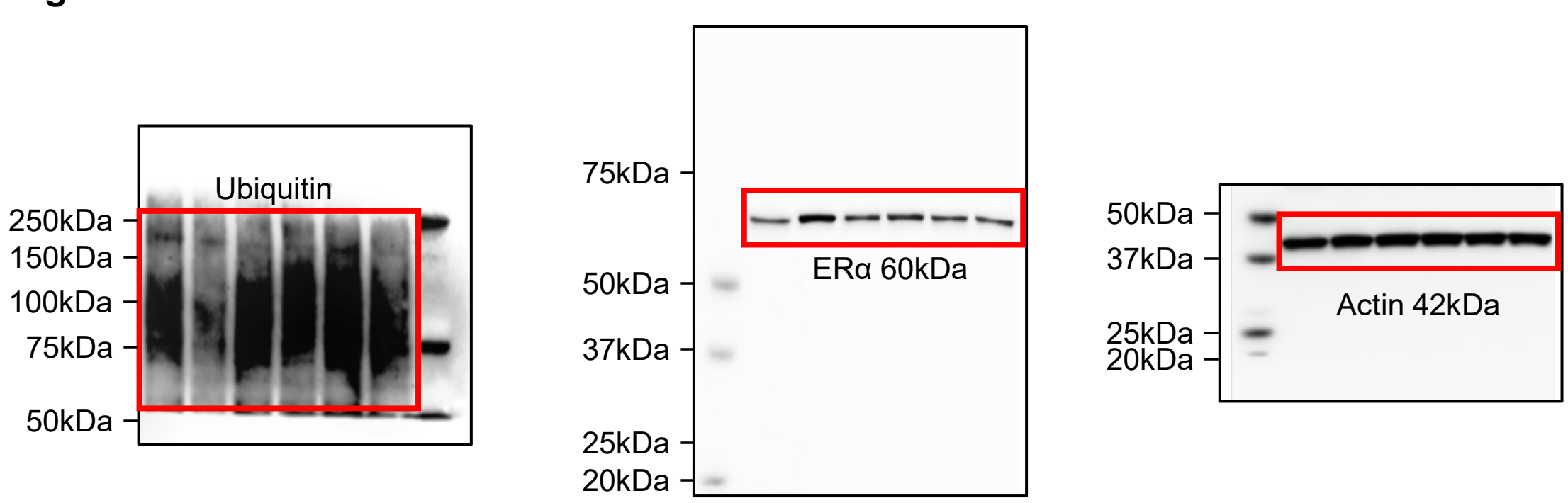

Supplement: Supplementary file 6 [file 43018_2026_1221_MOESM9_ESM.pdf]
